# RADAR-AD: assessment of multiple remote monitoring technologies for early detection of Alzheimer’s disease

**DOI:** 10.1186/s13195-025-01675-0

**Published:** 2025-01-27

**Authors:** Manuel Lentzen, Srinivasan Vairavan, Marijn Muurling, Vasilis Alepopoulos, Alankar Atreya, Merce Boada, Casper de Boer, Pauline Conde, Jelena Curcic, Giovanni Frisoni, Samantha Galluzzi, Martha Therese Gjestsen, Mara Gkioka, Margarita Grammatikopoulou, Lucrezia Hausner, Chris Hinds, Ioulietta Lazarou, Alexandre de Mendonça, Spiros Nikolopoulos, Dorota Religa, Gaetano Scebba, Pieter Jelle Visser, Gayle Wittenberg, Vaibhav A. Narayan, Neva Coello, Anna-Katharine Brem, Dag Aarsland, Holger Fröhlich, on behalf of RADAR-AD

**Affiliations:** 1 Fraunhofer Institute for Algorithms and Scientific Computing SCAI, Sankt Augustin, Germany; 2 Bonn-Aachen International Center for IT (b-it), University of Bonn, Bonn, Germany; 3 Janssen Research and Development LLC, Titusville, NJ United States; 4 Alzheimer Center Amsterdam, Neurology, Vrije Universiteit Amsterdam, Amsterdam UMC location VUmc, Amsterdam, The Netherlands; 5 Neurodegeneration, Amsterdam Neuroscience, Amsterdam, The Netherlands; 6 Centre for Research & Technology Hellas, Information Technologies Institute, Thessaloniki, Greece; 7 University of Oxford, Oxford, UK; 8 Ace Alzheimer Center Barcelona, Universitat Internacional de Catalunya, Barcelona, Spain; 9 Networking Research Center on Neurodegenerative Diseases (CIBERNED), Instituto de Salud Carlos III, Seville, Spain; 10 King’s College London, London, UK; 11 Biomedical Research, Novartis, Basel, Switzerland; 12 Memory Center, Geneva University and University Hospital, Geneva, Switzerland; 13 Laboratory Alzheimer’s Neuroimaging & Epidemiology, IRCCS Istituto Centro San Giovanni di Dio Fatebenefratelli, Brescia, Italy; 14 Centre for Age-related Medicine, Stavanger University Hospital, Stavanger, Norway; 15 Department of Clinical Medicine, University of Bergen, Bergen, Norway; 16 Alzheimer Hellas and Laboratory of Neurodegenerative Diseases, Aristotle University of Thessaloniki, Thessaloniki, Greece; 17 Central Institute of Mental Health, Medical Faculty Mannheim, University of Heidelberg, Mannheim, Germany; 18 Faculty of Medicine, University of Lisbon, Lisbon, Portugal; 19 Center for Alzheimer Research, Department of Neurobiology, Care Sciences and Society (NVS), Karolinska Institutet, Stockholm, Sweden; 20 Davos Alzheimer’s Collaborative, Geneva, Switzerland; 21 Novartis Pharma AG, Basel, Switzerland; 22 University Hospital of Old Age Psychiatry, University of Bern, Bern, Switzerland; 23 Stavanger University Hospital, Stavanger, Norway; 24 Department of Psychiatry and Neuropsychology, School for Mental Health and Neuroscience, Maastricht University, Maastricht, The Netherlands

**Keywords:** Alzheimer’s disease, Remote monitoring technologies, Wearables, Mobile applications, Discriminative capacity

## Abstract

**Background:**

Alzheimer’s disease (AD) is a progressive neurodegenerative disorder affecting millions worldwide, leading to cognitive and functional decline. Early detection and intervention are crucial for enhancing the quality of life of patients and their families. Remote Monitoring Technologies (RMTs) offer a promising solution for early detection by tracking changes in behavioral and cognitive functions, such as memory, language, and problem-solving skills. Timely detection of these symptoms can facilitate early intervention, potentially slowing disease progression and enabling appropriate treatment and care.

**Methods:**

The RADAR-AD study was designed to evaluate the accuracy and validity of multiple RMTs in detecting functional decline across various stages of AD in a real-world setting, compared to standard clinical rating scales. Our approach involved a univariate analysis using Analysis of Covariance (ANCOVA) to analyze individual features of six RMTs while adjusting for variables such as age, sex, years of education, clinical site, BMI and season. Additionally, we employed four machine learning classifiers – Logistic Regression, Decision Tree, Random Forest, and XGBoost – using a nested cross-validation approach to assess the discriminatory capabilities of the RMTs.

**Results:**

The ANCOVA results indicated significant differences between healthy and AD subjects regarding reduced physical activity, less REM sleep, altered gait patterns, and decreased cognitive functioning. The machine-learning-based analysis demonstrated that RMT-based models could identify subjects in the prodromal stage with an Area Under the ROC Curve of 73.0 %. In addition, our findings show that the Amsterdam iADL questionnaire has high discriminatory abilities.

**Conclusions:**

RMTs show promise in AD detection already in the prodromal stage. Using them could allow for earlier detection and intervention, thereby improving patients’ quality of life. Furthermore, the Amsterdam iADL questionnaire holds high potential when employed remotely.

**Supplementary Information:**

The online version contains supplementary material available at 10.1186/s13195-025-01675-0.

## Background

Alzheimer’s disease (AD) is a progressive neurodegenerative disease that affects memory, thinking, and behavior and is the leading cause of dementia in older adults [1]. Neuropathological characteristics of Alzheimer’s disease emerge 15 to 20 years prior to the onset of noticeable cognitive symptoms [2–4], making early detection crucial to provide the best possible care as anatomical and physiological changes become increasingly irreversible as the disease progresses. Identifying patients at a preclinical stage is particularly challenging, as they do not seek medical care due to the absence of cognitive decline [5], and the necessary tests to reveal these early changes can be costly and not widely accessible. This is particularly relevant given the recent availability of new disease-modifying drugs for AD, such as Leqembi [6] and Kisnula [7], which have the potential to slow down disease progression when administered in the early stages of the disease. Additionally, there are approved drugs that are used to treat patients symptomatically, such as donepezil or memantine, which can help manage symptoms [8]. Furthermore, combination therapy of these two medications could also delay nursing home placement [9]. An early diagnosis enables patients and their families to plan for the future, including critical considerations regarding care, financial, and legal matters.

Traditional neuropsychological assessments, primarily focused on cognitive and behavioral symptoms, are often the first step in the diagnostic process. Complementing these are more specific diagnostic procedures, such as lumbar puncture and brain molecular imaging, which are expensive, time-consuming, and not widely accessible due to their invasive nature. These procedures are typically limited to specialized memory clinics, making early disease diagnosis challenging for many individuals. In general, it is estimated that the economic burden of Alzheimer’s disease and related dementias will increase a lot during the following decades [10]. Therefore, developing cost-effective and user-friendly tools to support the existing diagnostic procedures is essential. We need diagnostic tools that can accurately and sensitively detect the functional deficits of AD pathology at the earliest possible disease stage and monitor the effects of intervention strategies in AD. In this study, we investigate the potential of Remote Monitoring Technologies (RMTs) for this purpose.

RMTs can help detect early symptoms of Alzheimer’s disease through a noninvasive, cost-effective method for collecting data on various functional parameters and digital biomarkers, including movement patterns, sleep quality, gait parameters, social engagement, and cognitive function, providing a comprehensive view of an individual’s health status [11, 12]. These technologies include smartphone apps, wearable sensors, smart home technology, and other remote monitoring devices that can be made widely accessible to people in low or middle-income countries. Currently, the field of respective technologies is rapidly developing, and several studies have shown their effectiveness in detecting symptoms of neurodegenerative diseases. For example, Zhan et al. proposed a smartphone app to detect Parkinson’s disease symptoms [13]. Bayat et al. used the driver’s age and derived metrics from an in-vehicle GPS logger to identify patients with preclinical AD [14]. In another study, Berron et al. utilized a smartphone application that employs tasks such as an object-in-room recall, complex scene recognition, and mnemonic discrimination for objects and scenes to effectively discriminate between different diagnostic stages, identifying MCI with good accuracy [15]. In general, the number of studies using RMTs in healthy and AD-affected populations dramatically increased in recent years [16]. However, many studies face strong limitations: some fail to incorporate data from evaluations conducted in home-based and real-life settings, others cover only short-term measurements, and few compare multiple RMTs [16]. Therefore, it is currently unclear which RMTs are the most suitable to detect functional decline symptoms robustly and accurately in patients with early-stage AD.

The **Remote Assessment of Disease and Relapse - Alzheimer’s Disease** (RADAR-AD; https://www.radar-ad.org/) study aimed to evaluate the potential of RMTs to accurately measure cognitive and motor functioning for individuals in different stages of AD [17]. It addressed the limitations of prior research by evaluating a comprehensive set of RMTs in a real-world environment over eight weeks. In this current paper, we employed a two-pronged analytical approach to assess their potential. First, we used statistical univariate analyses to identify RMT-derived features that significantly differentiate between study groups. Then, we deployed a machine-learning pipeline to evaluate how well these RMTs distinguish between the groups.

By employing this dual methodology, we aimed to answer critical questions: a) Can RMTs detect functional deficits at early-stage AD? b) Which specific RMT features are the most indicative? c) Furthermore, to what extent can machine-learning models accurately distinguish between different diagnostic groups by utilizing features acquired from RMTs? Collectively, this study contributes to clarifying the possibilities of RMTs in healthcare systems, either for earlier diagnosis or ongoing monitoring of AD progression.

## Methods

### RADAR-AD study cohort

The RADAR-AD study is an observational, cross-sectional study conducted across 13 European countries. It primarily aims to evaluate the effectiveness and reliability of RMTs in tracking functional decline in AD from preclinical to moderate stages compared to traditional clinical rating scales. Data collection took place between 2020 and 2023.

To be eligible for study participation, participants were required to meet the following inclusion criteria: being at least 50 years of age, maintaining a relatively good health status, or having a mild chronic disorder that was controlled by therapy or did not impair function. A prerequisite was having a partner willing to participate in the study, who could be a spouse, a relative, a caregiver, or a friend. Both the participant and their partner needed to converse in the language of the recruitment center and actively participate in various tests and questionnaires, with access to a smartphone being a prerequisite for both. Exclusion criteria included having a concurrent neurological or psychiatric condition that might interfere with their daily life activities or social interactions, or any other health conditions that could substantially affect their mobility, daily activities, or social engagements, such as, inflammatory disorders caused by the immune system, or recovery from recent trauma or stroke.

Neuropsychological assessments and in-clinic technologies (Altoida MD, Amsterdam iADL and Physiolog) were conducted during the initial visit, with other RMT data collected subsequently. The study comprises four distinct groups: healthy controls, preclinical AD, prodromal AD, and mild-to-moderate AD. To ensure that all AD patients had underlying AD disease, all participants had evidence of supra-threshold A$\upbeta$ burden based on amyloid PET and CSF analysis before inclusion. The control participants were cognitively unimpaired and matched the age and sex distributions as participants of the AD groups, preferably with confirmation of negative AD biomarkers. The study groups were defined according to FDA guidelines and assessed using the Mini-Mental State Examination (MMSE) [18] and the Clinical Dementia Rating (CDR) [19, 20].

The study received ethical approval in each participating country and aligned to the principles of the Helsinki Declaration of 1975, as revised in 2008. All participants and their informants provided written informed consent before any study procedures. For a schematic representation of the recruitment process, readers are referred to the appendix, which contains the flowchart in Supplementary Figure C.1. For a more comprehensive understanding of the RADAR-AD study, we refer to the papers by Owens et al. [21] and Muurling et al. [17, 22], as well as the research protocol available at the RADAR-AD website (https://www.radar-ad.org/our-research/project-deliverables). For details on the RADAR-AD cohort itself, please refer to Table 2 in the Results section.

### Evaluated RMTs

The RADAR-AD study included multiple tiers of RMTs. For this work, we focused on six RMTs from the main study and the Amsterdam iADL questionnaire for further analysis. We begin by providing details on the RMTs and their derived features. For a comprehensive list of the RMT-derived features, please refer to Supplementary Table A.3.

#### Fitbit Charge 3

The Fitbit Charge 3 is a fitness tracker that utilizes an accelerometer and a heart rate monitor and is equipped with sleep-tracking and activity-monitoring functions. The device features an interactive display for user engagement. For our study, participants were instructed to wear the device on their non-dominant wrist for eight weeks. Following the study’s conclusion, the collected data was analyzed and processed.

The extracted data was segmented into heart rate, sleep, and activity data. Heart rate information was calculated using the average, minimum, and maximum values recorded throughout the study. Additionally, we also examined hourly averages of the heart rate. Similarly, step data were analyzed by calculating average steps per day, as well as per hour of the day. Regarding sleep-derived features, we looked at the average number of hours spent asleep, awake, and in the various sleep stages while also considering the mean bedtime.

#### Axivity AX3

The Axivity AX3 is an additional accelerometer-based tracking device in our study for physical activity and sleep tracking. Participants were instructed to wear the device on their dominant wrist for eight weeks. While the Axivity AX3 does not include a display or heart rate monitor, it has a significantly longer battery life than the Fitbit Charge 3. As the device provides data at a more granular level, particularly raw 3D acceleration data, it was incorporated into our study. Previous research has already demonstrated the device’s suitability for this type of research [23].

In our study, we extracted a range of features from the Axivity AX3 device, including the acceleration magnitude, wear time, time spent in sedentary, light, and moderate-to-vigorous activity, as well as sleep-derived features. The average values of these features were reported separately for weekdays and weekends. Furthermore, we examined the average values per hour of the day for the weekdays.

#### Mezurio app

The Mezurio app is a smartphone application compatible with iOS and Android operating systems. The app is designed to evaluate an individual’s cognitive abilities through gamified cognitive tasks. The tasks included in the app are the Gallery Game, which requires the memorization of photo and swipe direction pairings; the Story Time game, which involves narrating and recalling a short comic from memory immediately and after a thirty-minute delay; and the tilt task measuring executive function, which requires moving a cursor to a target by tilting the device. Participants in our study were directed to use the app for at most 10 minutes per day. Previous research has demonstrated the benefits of the Mezurio app [24].

Out of the various tasks within the Mezurio app, this paper utilized the Story Time task. As briefly mentioned above, this task contains verbal learning of comic strips to form a story (similar to the picture description task predominantly used as a speech collection method) and immediate and delayed recall from the memory. In this paper, we only analyzed speech-derived features from the learning subtask. These features include speech fluency features, including the number of syllables and pauses, average pause duration, speech rate, articulation rates, and hesitation ratio. In addition, other prosodic and voice quality features were extracted using the *openSMILE* python package with the *GeMAPS* feature set as a parameter [25].

#### Altoida Medical Device (Altoida MD)

The Altoida MD application is developed for smartphones and tablets and combines augmented reality and motor-cognitive tasks. This app aims to simulate complex activities of daily living to detect early signs of cognitive decline in individuals. Notably, prior research has highlighted the application’s effectiveness, reporting a balanced accuracy of 93 % in forecasting cognitive decline within five years among amyloid-positive individuals transitioning from mild cognitive impairment (MCI) to Alzheimer’s disease (AD) [26]. The study participants engaged with the Altoida MD app during their initial clinic visit.

The application uses the device’s embedded sensors to collect data based on the user’s interaction with various tasks. These sensors capture intricate details of participants’ behavior, including hand micromovements, screen touch pressures, walking speed, navigation trajectory, and cognitive processing speed, thereby painting a comprehensive behavioral profile. The collected sensor data is subsequently processed by the Altoida application for further analysis.

For our study, we focused on two main feature categories. The first comprises cognitive domain scores (CDS). These scores assess the participant’s cognitive abilities in various areas, such as perceptual-motor coordination, complex attention, cognitive processing speed, inhibition, flexibility, visual perception, planning, prospective memory, spatial memory, fine motor skills, and gait. These scores are derived from raw sensor data. For each of these features, a percentile score is determined by comparing it to a control group of individuals who are healthy and share similar characteristics in terms of age and gender. The second prominent feature is the Digital Neuro Signature (DNS) score. This score personalizes the test performance in relation to the participant’s demographic group considering factors such as age, sex, and years of education. Serving as a primary measure for differentiating between normal cognition and Mild Cognitive Impairment (MCI), the DNS score is meant as an approach to detect cognitive decline effectively.

#### Amsterdam iADL

The A-iADL is a questionnaire designed to evaluate instrumented activities of daily living (iADLs). This tool can be administered by a caregiver using a personal computer or smartphone. The efficacy and validity of this questionnaire were established in a previous study that employed a cohort of memory clinic patients [27]. Further research has also illustrated its capacity to detect functional decline [28]. In this study, the questionnaire (short version) was administered during the initial in-clinical visit.

In our experiments that explored the discriminative abilities of A-iADL, we utilized the calculated $\Theta$ score from the short version of the questionnaire as the solitary feature. This score is derived from the questionnaire and represents the underlying attribute of “daily functioning”. Parts of the A-iADL from the short version of the questionnaire were also utilized to create a composite score, which is detailed further in the supplementary information (see Supplementary Table B.1).

#### Physilog sensors

The Physilog Gait Sensors are wearable devices equipped with accelerometers and gyroscopes, which measure triaxial acceleration and angular velocities. Gait spatiotemporal descriptors such as speed, symmetry, and variability can be accurately derived with measurement agreement comparable to that obtained from optical motion capture systems or electronic walkways [29]. These sensors have been validated in numerous patient groups, with normative data available for cognitively healthy older adults [30]. Their user-friendly nature and ability to provide detailed gait analysis make them a valuable tool for healthcare professionals and researchers working in the field of mobility and gait analysis.

In RADAR-AD study the Physilog gait sensors were placed on both feet and the hip to measure gait parameters while study participants performed two tests. The first test was the Dual-Task assessment, in which participants were instructed to walk for one minute and then to repeat the same action while counting aloud backwards from 100. The second test was a timed up-and-go (TUG) test, where participants initially sat, stood up, and walked for three meters before turning around to sit down again. The sensors collected data on angular velocities and accelerations, which was subsequently processed by proprietary algorithms [30] to derive gait speed, cadence, duration, angles, and other features. In the Dual-Task assessment the Dual-Task-Effect (DTE) was calculated. It is defined by $\text {DTE}_{x} = 100 \times \frac{\text {x}_{\text {DUAL}} - \text {x}_{\text {SINGLE}}}{\text {x}_{\text {SINGLE}}}$ and assesses the difference between these two tasks.

#### Banking application

The Banking application is a smartphone application that simulates a bank withdrawal process. Individuals are required to enter a pin, specify the amount they wish to withdraw, and confirm their selection. During this process, data is collected on factors such as duration and errors. Originally developed as part of the multisensor assessment and monitoring system in the Dem@Care project [31], the app was designed to evaluate functional abilities related to financial management. For this study, the application has been expanded and improved, with added support for multi-lingual use.

The application’s features are specifically designed to measure the time taken for various stages of the withdrawal process and the number of attempts required by participants to complete the task successfully. Data is reported on the number of correct attempts and their duration for the individual stages, thus providing valuable insights into the participant’s financial management abilities.

### Assessment of RMTs’ discriminative abilities

**Fig. 1:**
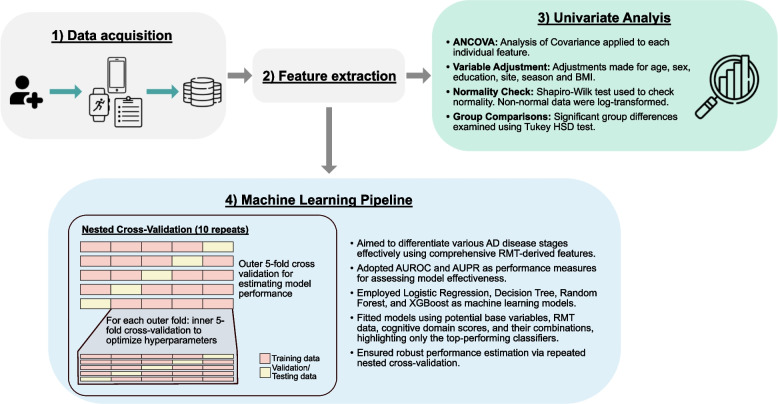
Overview of the study workflow to evaluate the discriminative abilities of RMTs in AD progression. The process involves 1) data acquisition and 2) feature extraction from RMTs, followed by 3) univariate analyses (ANCOVA and Tukey HSD tests) to detect significant feature differences across study groups, and 4) a machine-learning pipeline, including Logistic Regression, Decision Tree, Random Forest, and XGBoost models, to quantify how well stages of AD can be distinguished using RMT-derived data

We structured our approach into two distinct segments to thoroughly assess the discriminative potential of the selected RMTs in differentiating between all pairwise combinations of healthy controls, preclinical, prodromal, and mild-to-moderate AD stages. The first part involves univariate analysis for identifying significant feature differences, while the second employs a machine learning pipeline to capture complex interactions and robustly quantify RMT performance. An outline of this methodology is provided in Fig. 1.

#### Univariate statistical analysis of RMTs

In the first segment of our study, we applied univariate analyses using Analysis of Covariance (ANCOVA) for each individual RMT feature. Here, each RMT feature was used separately as the dependent variable, while the group acted as the independent variable. Features generated on an hourly basis (e.g., steps and heart rate per hour of the day) were not considered during this analysis. Based on established literature, we adjusted the analyses for age, sex, and years of education, as these demographic factors have been consistently associated with AD risk and progression [32–34]. We also included the site as a covariate. For features derived from Fitbit, Axivity, and Physilog, the models incorporated Body Mass Index (BMI) as an additional adjustment. For the wearables Fitbit and Axvitiy, we also included the season to account for differences in activity patterns that are based on climatic or seasonal effects. In the case of Altoida features, the models were corrected exclusively for the site as these features, defined by Altoida developers, already considered age, sex, and years of education in their initial model. We applied the Shapiro-Wilk test to determine the normality of feature distribution, and if found to be non-normal, we log-transformed the data. Finally, if the ANCOVA yielded a significant group difference, we conducted a Tukey Honestly Significant Difference (HSD) posthoc test for group-wise comparisons. *P*-values were subsequently adjusted for multiple testing across all features using the Holm method.

#### ML-based assessment of RMTs’ discriminative abilities

The second segment of our study utilized a machine learning methodology to quantify the effectiveness of various RMTs in distinguishing different stages of Alzheimer’s Disease and healthy controls while using all features derived from a particular RMT jointly. Our focus was on binary classification tasks, such as differentiating between healthy controls and preclinical AD or between preclinical and mild-to-moderate AD. These tasks were integral to assessing the discriminative capabilities of the RMTs.

Our evaluation process involved four machine learning models - Logistic Regression with elastic net penalization [35, 36], Decision Tree [36, 37], Random Forest [36, 38], and XGBoost [39]. We chose these classical machine-learning algorithms due to the small amount of available data (229 participants) and their reputation to work well even in such scenarios. With respect to the small sample size, we implemented repeated Nested Cross-Validation (NCV) with ten repeats and five folds to the dataset extracted from each RMT. This approach allowed us to optimize hyperparameters effectively while providing a reliable performance estimation, particularly crucial for models like XGBoost that are sensitive to hyperparameter selection. For this step, we utilized the Optuna library [40], and we present the parameter options and their ranges in Table 1. To understand which features contribute most to the model predictions, we employed the approach suggested by Scheda and Diciotti to calculate the Shapley additive explanations (SHAP) [41, 42] in a repeated NCV setting [43]. SHAP values quantify the influence that each feature has on a model’s prediction for a given instance. The magnitude of these values reflects the degree of influence, while the sign shows the direction of its influence. In each fold, these values are calculated for both the train and test sets. Subsequently, these values are average per participant across the repetitions, enabling assessment of both local and global feature importance.

**Table 1:** Hyperparameter ranges for each classifier

Classifier	Hyperparameter	Range
Logistic Regression with elastic net penalty	C	[0.01, 2]
	L1-ratio	[0.01, 0.99]
Decision Tree	max_depth	[10, 100, step=10]
	min_samples_leaf	(1, 2, 4)
	min_samples_split	(2, 5, 10)
Random Forest	n_estimators	[600, 1400, step=200]
	max_depth	[10, 100, step=10]
	min_samples_leaf	(1, 2, 4)
	min_samples_split	(2, 5, 10)
XGBoost	eta	[0.01, 0.7]
	gamma	[0, 0.5, step=0.1]
	max_depth	[1, 22, step=1]
	n_estimators	[50, 400, step=25]

To assess performance, we employed two metrics: the Area Under the Receiver Operating Characteristic (AUROC) and the Area Under the Precision-Recall Curve (AUPR). The AUROC is a widely used metric that measures a model’s ability to discriminate between two group, with values ranging from 0.5 (equivalent to random change) to 1 (indicating perfect discrimination). In clinical studies, AUROC values can be interpreted as follows: values $\ge$ 0.9 indicate excellent performance, values $\ge$ 0.8 indicate considerable performance, values $\ge$ 0.7 indicate fair performance, values $\ge$ 0.6 indicate poor performance, and values $\ge$ 0.5 indicate failure [44]. However, the AUROC can be overly optimistic in scenarios with imbalanced data. Therefore, we also utilized the AUPR, which provides a more robust measure in such scenarios. Like the AUROC, the AUPR ranges from 0 to 1, with higher values indicating better performance. To ensure the robustness of our results, we reported the average AUROC and AUPR values from the outer folds for each of the NCV repeats. This approach resulted in ten values for each metric, enabling us to account for any potential variability in the performance of our models.

Notably, our dataset contained missing values as not all participants used each and every RMT. For instance, Altoida (N=135), Physilog (Dual=176, TUG=168), and Mezurio (N=163) were underrepresented compared to others. The overlap between the different study groups and the RMTs is further emphasized in Table 2 and Supplementary Figure A.3. To address the issue of missing data, we employed a k-Nearest Neighbors-based imputation method [36, 45] to impute missing values as part of the LR, DT, and RF training pipeline. The imputation model was fitted on the training data and predicted missing values in the testing data. However, we did not use this imputation method for the XGBoost model since this method can handle missing values implicitly.

**Table 2:** Characteristics of the RADAR-AD study cohort

		HC	PreAD	ProAD	MildAD	Overall
**a) Cohort-specific**	N	69	39	65	56	229
	Female	55.1 %	59.0 %	41.5 %	44.6 %	49.3 %
	Age	67.3 (7.5)	70.7 (5.8)	69.7 (7.7)	70.0 (8.9)	69.2 (7.7)
	Education Years	14.5 (3.6)	15.6 (2.8)	14.6 (4.6)	13.7 (4.2)	14.5 (4.0)
	BMI	25.7 (3.1)	24.6 (3.9)	25.1 (3.6)	24.0 (3.6)	24.9 (3.6)
	APOE E4 status^a^	13/4/52	6/14/19	11/24/30	5/25/26	35/67/127
	CDR	0.0 (0.0)	0.0 (0.1)	0.5 (0.1)	1.1 (0.4)	0.4 (0.5)
	CDR Range	0.0	0.0–0.5	0–0.5	0.5–2.0	0.0–2.0
	MMSE	29.2 (0.9)	29.1 (1.0)	26.5 (2.3)	21.7 (3.3)	26.6 (3.7)
	MMSE Range	27–30	26–30	19–30	16–29	16–30
	A-iADL	68.2 (2.6)	66.1 (3.9)	58.6 (7.1)	46.8 (8.0)	59.8 (10.2)
**b) RMT-specific**	Altoida	57	28	38	12	135
	Axivity	60	32	56	44	192
	Banking	62	34	57	50	203
	Fitbit	68	39	56	52	215
	Mezurio	66	27	44	26	163
	Physilog (Dual)	56	31	49	40	176
	Physilog (TUG)	52	31	47	38	168
	iADL	67	37	63	55	222

Shown are the characteristics of the study groups Healthy Control (HC), Preclinical AD (PreAD), Prodromal AD (ProAD), and Mild-to-Moderate AD (MildAD), as well as the overall study cohort. a) Descriptive statistics per group, including mean values and standard deviation (where applicable), the number of participants carrying or not carrying APOE E4 alleles, and MMSE and CDR ranges.. b) Number of participants for which RMT/Questionnaire data was obtained

^a^Order: participants without APOE E4 alleles, participants with APOE E4 alleles, participants with unknown APOE E4 status

In this study, we evaluated different scenarios to understand the capacity of our models to distinguish between diagnostic groups based on a set of base variables, RMT-derived data, and also questionnaires and clinical tests. The diverse training approaches used in our study are detailed below:

**Base:** This model employs only base variables, such as sex, age, study site, and years of education, to evaluate the model’s performance to distinguish between groups based purely on demographic factors.
Base*: This augmented model incorporates two additional variables: Body-Mass-Index (BMI) and the season of the year. The Base* Model aims to account for both fitness-related parameters and the potential seasonal climate variations, which may influence the performance of specific RMTs.
**RMT:** The RMT model merges variables from the base model with features derived from RMTs, exploring the additional contribution of RMTs to enhance classification performance.
**FDS:** The FDS model integrates base variables with a series of composite scores, denoted as Functional Domain Score (FDS) throughout this paper. The purpose of this model is to discern disease stages using these traditional measures associated with each cognitive domain. For details on its calculation, refer to Supplementary Section B.1.
**RMT+FDS:** Expanding on the RMT model, the RMT+FDS model additionally incorporates FDS. This model investigates the potential advantages of combining these scores with RMT-derived features and base data.

## Results

This study used two distinct yet complementary analyses to evaluate the efficacy of RMTs in distinguishing between various stages of AD, as outlined in Fig. 1. In the first stage of our study, we performed an univariate analysis. This allowed us to isolate and investigate each feature and consequently identify those that held significant potential for differentiating between the four study groups. Recognizing that this approach does not capture complex interactions or patterns that could be important to distinguish between the different stages, we leveraged the entire set of features from each RMT in the subsequent stage. We used a machine learning pipeline to further investigate the capabilities of each individual RMT and quantify discrimination performance. Before discussing the results, a brief overview of the dataset obtained from the RADAR-AD study will be provided.

### Characteristics of study population

The RADAR-AD study population comprised 229 participants from 13 European sites, categorized into four groups: healthy controls (HC), preclinical AD (PreAD), prodromal AD (ProAD), and mild-to-moderate (MildAD). Preclinical participants exhibit amyloid pathology but are cognitively unimpaired, whereas those in the prodromal stage display minor cognitive impairment. Participants from the mild-to-moderate AD group experienced more extensive cognitive deficits. The groups were closely matched in demographic and health-related variables that could influence the study results. Specifically, there were no statistically significant differences in age (ANOVA, p=0.26), education years (ANOVA, p=0.30), and BMI (ANOVA, p = 0.18) between the four groups. Similarly, the sex distribution across groups exhibited no significant differences (Chi-squared test, p=0.30). All *p*-values were corrected with the Holm method for multiple testing. The distributions of these variables are depicted in Supplementary Figure A.2 and additional descriptive statistics on the cohort and the number of participants for each RMT are provided in Table 2.

### Univariate assessment of RMT features for Alzheimer’s disease discrimination

**Fig. 2:**
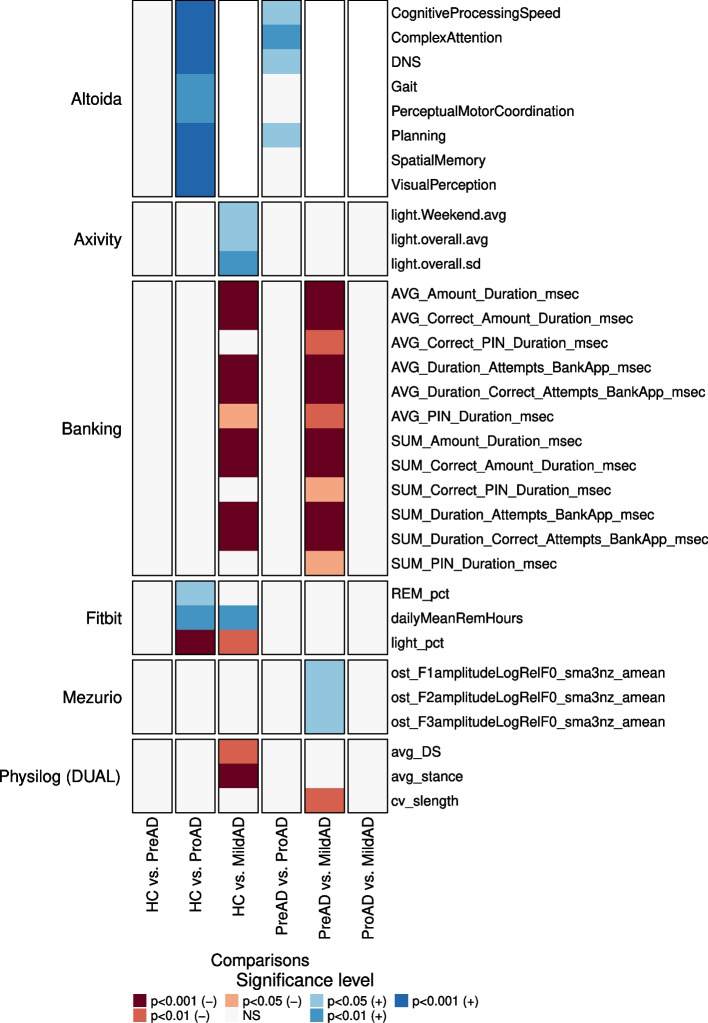
Statistical Analysis of RMT Features. The figure illustrates a comprehensive univariate statistical analysis of the RMTs’ features. Each row depicts the results obtained for the six comparisons, while the label on the left indicates the corresponding RMT affiliation. Statistical outcomes of the Tukey HSD test (conducted upon significant ANCOVA) are presented in the heatmap itself. The *p*-values are expressed through a color-coded system, with the specific coding shown in the provided legend. The symbols “-” or “+” are used to denote the direction of the test statistic. The *p*-values have been adjusted for multiple testing

During the initial research phase, we examined the potential of specific features to effectively distinguish between various diagnostic stages. A sample of these features is shown in Fig. 2, where both the ANCOVA-derived *p*-values and at least one Tukey HSD test *p*-value were statistically significant in differentiating between diagnostic groups. It is important to clarify that in this section, “significance” refers to statistical significance, with *p*-values indicating differences determined by our statistical analysis. We have complemented these *p*-values with effect size values to provide a more comprehensive understanding of the differences. indicating notable differences determined by our statistical analysis. Furthermore, the *p*-values in each group comparison have been adjusted for multiple testing. For a complete list of each feature and its corresponding outcomes, including test statistics and effect size estimates, see Supplementary Table C.1.

Our analysis identified significant features for the following RMTs: Altoida, Axivity, the Banking app, Fitbit, Mezurio, and the Physilog DUAL task. However, no significant feature was detected for the Physilog TUG task. Furthermore, we did not uncover any significantly differentiated features between healthy controls and preclinical AD and prodromal and mild AD.

A subset of features significantly separated healthy controls from prodromal AD subjects. However, these features were derived exclusively from Altoida and Fitbit. For Altoida, several cognitive domain scores, such as cognitive processing speed, complex attention, gait, perceptual motor coordination, planning, spatial memory and visual perception, and the DNS score were significantly lower for prodromal AD participants. For Fitbit, less time spent in the Rapid Eye Movement (REM) sleep stage and more time spent in the light sleep stage were associated with prodromal AD.

In contrast, a greater proportion of the examined features demonstrated statistical significance when comparing healthy controls with participants in the mild to moderate AD stage. This demonstrates that the explored RMTs effectively distinguish between these groups with greater variation in behaviors and functional abilities based on the disease stage. Significant features were identified in Axivity, the Banking app, Fitbit, and Physilog (Dual). The average time spent in light physical activity over the eight-week study period, a feature derived from the Axivity measurements, was significantly higher in healthy controls. Similar to the comparison with the participants in the prodromal stage, the amount of REM sleep was also significantly lower for participants with mild AD. The significant features derived from the Phsyilog Dual task indicate that mild AD participants exhibited an altered gait cycle, spending more time in both the double support phase and the single support phase compared to healthy individuals. Moreover, several Banking app features differed significantly between these groups. For example, mild AD participants took longer to enter their PIN and transaction amount, signaling declining financial capabilities with disease progression.

Importantly, no Altoida data from the mild AD cohort were used in our experiments. The RMT was discontinued for this subject population after initial testing with a small group of participants with mild AD (N=12). The tests revealed difficulties with fine motor tasks like drawing a circle on a smartphone, suggesting even greater challenges when encountering the more complex tasks of this application. As a result, the data from these 12 participants were excluded from our analyses.

When distinguishing between individuals in the preclinical and prodromal stages, it was observed that only Altoida provided features that varied significantly, specifically in the scores for Cognitive Processing Speed, Complex Attention, and DNS.

Several features from the Banking app, Physilog (Dual), and Mezurio presented significance when distinguishing preclinical from mild AD. As previously, in the healthy controls versus mild AD cases, mild AD patients took longer to enter, such as their PIN and transaction amounts. Amongst the Physilog (Dual) features, only the coefficient of variation for the stride length was significant, indicating that the length of strides is less consistent among the mild AD group. Finally, two features derived from the Mezurio data were found to be significant: the duration of the voiced and unvoiced segment lengths.

### Comprehensive examination: machine learning assessment of RMT capabilities

In the next phase, we investigated the RMTs’ capabilities in differentiating AD stages by applying four machine learning models in different scenarios. Specifically, we fitted Base models with variables such as age, years of education and sex , RMT-based models with the RMT-derived features, and FDS-based models with a series of composite scores derived from traditional assessments and questionnaires. Notably, we chose to use all available features rather than only the significant ones from the univariate analysis. Several factors drove this decision: Firstly, the univariate analysis served as an exploratory step aiming at finding out which features differ between groups. It was conducted on the entire dataset and thus, limiting the features to the significant ones would introduce information leakage into the ML pipeline. Second, multivariate machine learning algorithms can identify complex interactions between features that could be missed if only the significant features were selected, potentially introducing additional bias. For further details on our approach, we refer to the Methods section.

**Fig. 3:**
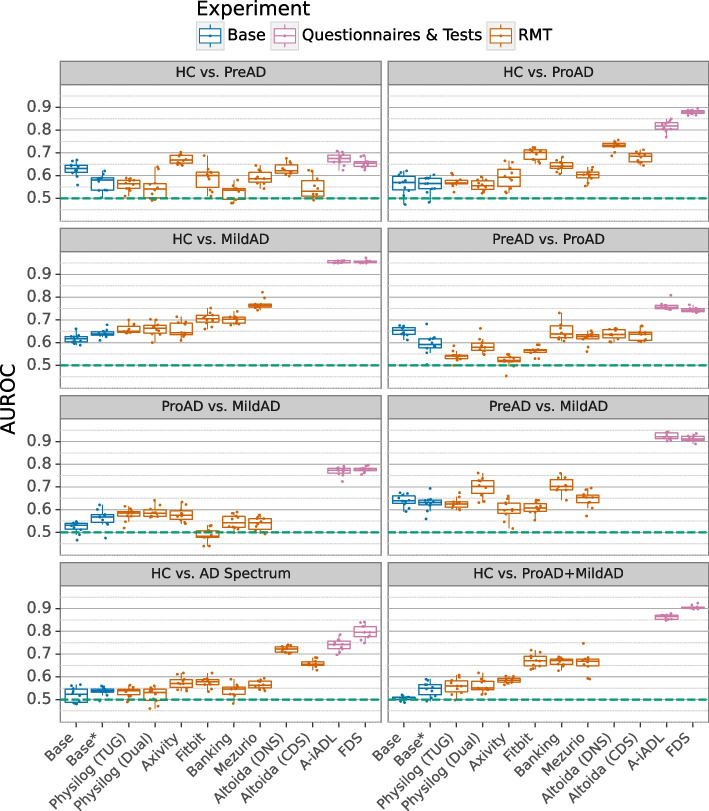
Discriminative abilities of different RMTs and their performance comparison across different disease stages. The figure depicts the Area Under the Receiver Operator Characteristic (AUROC). As we focused on optimal performance rather than specific classifiers to emphasize the highest discrimination ability, we show the AUROC only for the best-performing machine learning model in each experiment. The red and green boxes represent the base models, while the purple boxes illustrate the models trained solely on RMT data. The blue boxes depict the performance achieved for the questionnaire and test-based assessments (A-iADL and the functional domain scores derived from multiple questionnaires)

Figure 3 shows the results of the best classifier from these variants, with each subplot comparing two diagnostic groups. The boxplots show the AUROCs obtained through a repeated NCV. More detailed results, including the RMT+FDS variant and the results per ML classifier, further feature importance plots, as well as the AUPR metrics can be found in the supplementary materials (Supplementary Figures C.1 to C.17, Supplementary Table C.2). In the following sections, we elaborate on the performance of the various models and provide information on the most important features when a model significantly outperforms its corresponding Base model.

#### Base Model Performance

We discovered that the Base and Base* models failed or performed relatively poorly in every scenario with average AUROCs ranging between 50.4 and 65.1 % across the six comparisons. This indicates that demographic and study-related data alone cannot be used to distinguish study groups accurately. This aligns with our analysis of the features age, sex, BMI, and years of education, which showed no significant differences between groups and suggests that the benefits observed for the other models shown in Fig. 3 are primarily attributed to the inclusion of additional features.

#### HC vs. PreAD

Neither the RMT nor FDS-based models demonstrated fair discrimination abilities in the HC vs. preclinical AD comparison, with the average AUROCs ranging between 52.4 % and 67.2 %. For most RMTs, we matched the Base model scores closely. Only for Axivity and Altoida (DNS)-based models, the discrimination was comparable (67.1 and 62.9 %, respectively) to that of A-iADL and FDS (67.2 % and 65.4 %, respectively).

#### HC vs. ProAD

The FDS and A-iADL-based models demonstrated considerable discrimination ability in the HC vs. prodromal AD comparison, achieving average AUROCs of 87.9 and 81.6 %, respectively. Similarly, some RMTs, such as Altoida (CDS=68.1 %, DNS=73.0 %) and Fitbit (69.3 %) performed fair, although the performance was significantly lower. In contrast, the Axivity, Physilog, and Mezurio-based models performed poorly. Particularly, the Physilog-based Dual and TUG models performed the worst, with AUROCs of 55.7 and 57.1 %, respectively, indicating that the discriminant abilities with these RMTs are closer to a random guess. Both models performed at the same level or worse than the respective Base model.

**Fig. 4:**
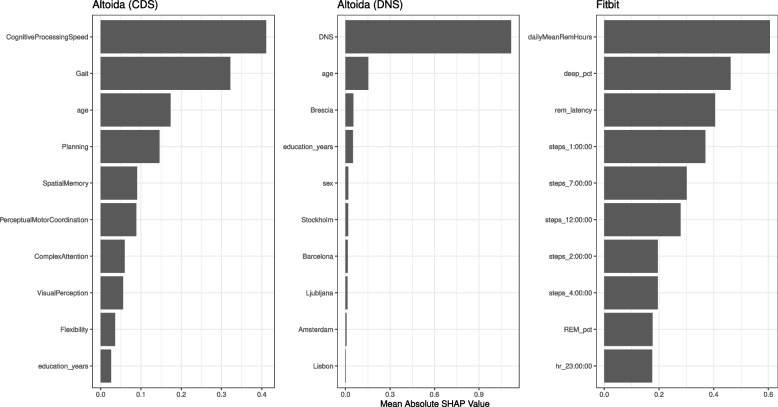
Distinguishing Features for HC vs. ProAD: This plot depicts the top ten features with the highest mean absolute SHAP values, offering insights into their significance in the Altoida and Fitbit-based models

Figure 4 highlights the features that drive the model’s decision with respect to Altoida and Fitbit. Depicted are the ten features with the highest mean absolute SHAP values. A higher value indicates a more substantial impact on the models’ prediction. In the Altoida (CDS) model, Cognitive Processing Speed, Gait, age, and Planning stood out as the most influential features. For the Altoida (DNS) model, the DNS feature is, as expected, the most important feature. In the Fitbit-based model, factors such as the average hours spent in REM, the ratio of deep sleep, and the REM latency were most important, followed by the number of steps recorded at several time points throughout the day.

#### HC vs. MildAD

The performance of the FDS and A-iADL-based models was excellent in discriminating between HC and mild AD groups, with average AUROCs of approximately 96 % in both cases. Among the RMT-based models, the Mezurio-based model performed best with an average AUROC of 76.9 %. Following that, the Fitbit-based model achieved an AUROC of 70.4 %. In contrast, the Physilog and Axivity-based models showed only marginal improvement compared to the respective Base model.

**Fig. 5:**
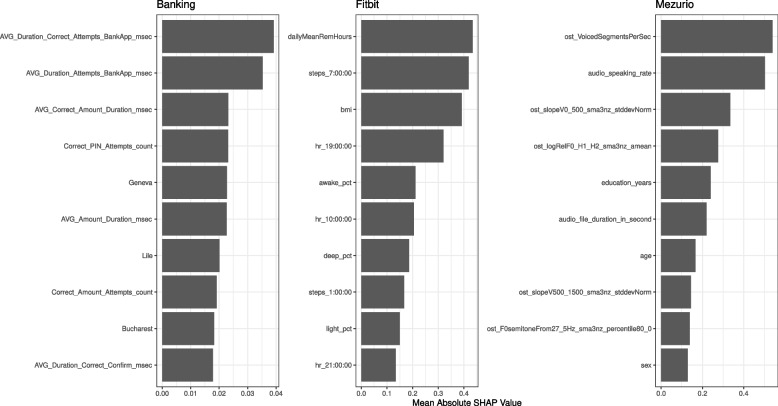
Distinguishing Features for HC vs. MildAD: This plot depicts the top ten features with the highest mean absolute SHAP values, offering insights into their significance in the Fitbit and Mezurio-based models

In the Mezurio-based model, key features included the number of voiced segments per second and the speaking rate (Fig. 5). Complementing these were complex voice-derived features such as the standard deviation of the spectral envelope between 0 and 500 Hz, and the log ratio of harmonic components. Furthermore, demographic attributes like years of education, age, and sex were among the top ten features. For the Banking app, the most important features were the duration spent at various stages within the app and the number of attempts to enter the correct PIN. Conversely, in the Fitbit-based model, BMI was among the most significant features, alongside several activity and sleep-related features, including the proportions of deep, light, and REM sleep stages.

#### PreAD vs. ProAD

In this comparison, the FDS-based model demonstrated fair performance with an average AUROC of 74.4 %, while the A-iADL-based model was equal with 76.1 %. Most RMT-based models achieved AUROCs comparable to the respective Base models. Only the Banking app-based model achieved, on average, slightly better results (64.9 %). The activity trackers Axivity and Fitbit performed the worst, with average AUROCs of 51.9 and 56.3 %, respectively, even lower than the AUROC of the Base* model (59.4 %).

#### ProAD vs. MildAD

In comparing prodromal and mild AD, A-iADL and FDS-based models achieved fair AUROCs of 76.9 % and 77.7 % respectively. Nonetheless, all evaluated RMTs failed in this assessment, with none surpassing an average AUROC of 59 %. Their performance was equivalent to that of the Base models.

#### PreAD vs. MildAD

Similar to the discrimination of healthy control and mild AD groups, the FDS and A-iADL-based models performed remarkably well in distinguishing mild AD participants from healthy controls. Both achieved very similar AUROC values (FDS=91.3 %, A-iADL=92.4 %). Among the RMTs, the Banking app and Physilog (Dual) based models achieved the best performances with AUROCs of 70.6 % and 69.9 % respectively.

#### HC vs. AD Spectrum

When comparing healthy controls to the entire AD spectrum, the FDS and A-iADL-based models demonstrated fair performance, with AUROCs of 79.8 % and 74.0 %, respectively. Apart from Altoida, which performed best among the RMTs (CDS=65.8 %, DNS=72.1 %), all other RMTs failed, achieving AUROCs of less than 58 %.

#### HC vs. ProAD + MildAD

Finally, in comparing healthy controls with prodromal and mild-to-moderate AD, the FDS and A-iADL-based methods showed good to excellent performance with AUROCs of 90.5 % and 86.3 %, respectively. Among the RMTs, the Banking app, Fitbit, and Mezurio performed best with AUROCs between 66.0 % and 67.1 %. Notably, Altoida data was excluded from this evaluation as it was the same as in the healthy controls versus prodromal AD comparison, with the mild-to-moderate group excluded.

#### Pairwise Combinations of RMT and FDS Data

Lastly, the results of the pairwise combination of the RMT and FDS data are shown in Supplementary Figure C.1. The objective was to assess the potential for further improvement by combining conventional questionnaires and RMTs. We observed that the performance of the models fitted on the paired data of the respective RMTs and FDS slightly exceeded the performance of the FDS-only model in some cases (HC vs. MildAD and PreAD vs. MildAD). However, the improvement was minimal, suggesting that simply combining information from both data sources does not provide significant benefits.

## Discussion

In this study, we conducted an extensive analysis to investigate the efficacy of RMTs in detecting different stages of Alzheimer’s disease. Our univariate analysis revealed only a few features with significant discriminatory potential across all RMTs. No significant differences were found between healthy controls and subjects with preclinical AD, highlighting the challenge of detecting functional differences in early stages. Similarly, no significant differences were found between the two later stages: prodromal and mild-to-moderate AD. Nevertheless, our findings suggest that RMTs help identify individuals in either the prodromal or more advanced mild-to-moderate stages of AD compared to healthy controls. For these stages, we found several differences compared to healthy subjects related to reduced physical activity (Axivity), less REM sleep (Fitbit), altered gait patterns (Physilog Dual), and decreased cognitive functioning (Altoida, Banking app).

The subsequent machine learning analysis demonstrated that using univariate analysis alone for assessing discrimination ability is limited. In some cases, the univariate analysis did not show significant differences, but the respective RMT-based ML models performed well in distinguishing between study groups. For example, Axivity was the best RMT for healthy controls versus preclinical AD, while Mezurio excelled for healthy controls versus mild-to-moderate AD, despite identifying no distinct features. This suggests that using a combination of all features helps to effectively distinguish the stages of AD and healthy controls and capture complex patterns that could be missed in a univariate analysis.

While some RMTs exhibited good performance levels in discriminating prodromal and mild-to-moderate AD stages from healthy controls, their effectiveness was more limited in other comparisons. For differentiating healthy controls versus preclinical AD, preclinical versus prodromal AD, and prodromal versus mild-to-moderate AD, the best RMTs only marginally outperformed or were on par with baseline models.

However, RMTs like Altoida, Mezurio, and Fitbit yielded encouraging results in distinguishing healthy individuals from those in prodromal and mild-to-moderate AD stages, suggesting their potential use in these specific stages. Unfortunately, these same RMTs demonstrated poor performance in distinguishing between prodromal and mild-to-moderate AD stages. Furthermore, participants with mild-to-moderate AD had difficulties using the Altoida app. These findings indicate that while they may be helpful for initial differentiation from healthy controls, they may not be suitable for tracking disease progression throughout these stages.

In addition to individual RMT analysis, the effectiveness of combining RMT and FDS data was assessed. While models trained on RMT and FDS data only occasionally performed better than FDS-only models, there was little or no benefit in most of the comparisons. Nevertheless, exploring a multimodal approach in which features from different RMTs or questionnaires are combined should be investigated in future studies, as this was not investigated in this study and could perform better than models trained on data from single RMTs.

Despite the many strengths of the RADAR-AD study, which entails a multicentre, multinational cohort including a range of AD stages from the preclinical to moderate dementia, with the diagnosis supported by biomarkers and the use of several RMTs, it is essential to recognize certain limitations: although the recruitment of 229 participants exceeded the target of 220, preclinical AD participants are slightly under-represented (PreAD=39) compared to the other groups (HC=69, ProAD=65, MildAD=56). Furthermore, it should be noted that not all RADAR-AD study participants used every RMT. For example, the number of data available for the Altoida app (N=135), the Physilog tasks (Dual=176, TUG=168), and the Mezurio app (N=163) were lower than that of other RMTs such as Axivity (N=192), the Banking app (N=203), or Fitbit (N=215) (Table 2 and Supplementary Figure A.3 provide additional information). This discrepancy necessitates a greater dependence on data imputation techniques and could be an additional source of error. Moreover, some RMTs, such as the Physilog devices, were only used once; therefore, these measures’ test-retest reliability cannot be assessed. Finally, the cross-sectional design precludes the longitudinal assessment of the change over time in these RMTs.

Nevertheless, our research findings contribute substantially to the field since, to the best of our understanding, apart from RADAR-AD, no other study has gathered cross-sectional data for a sizable cohort and multiple RMTs. Our findings suggest that Altoida, Mezurio, and Fitbit-based models have the potential to distinguish individuals in the prodromal stage from healthy controls. The findings related to Altoida align with previous studies, demonstrating the app’s ability to produce scores comparable to traditional neuropsychological tests [46]. Furthermore, machine learning models trained on these features exhibited impressive predictive capabilities, accurately forecasting the transition from Mild Cognitive Impairment to Alzheimer’s Disease [47]. In a similar vein, a remote digital memory composite (RDMC) that integrated three different memory tests achieved a high diagnostic accuracy in distinguishing cognitively impaired individuals from those who are unimpaired [15]. Besides these specified examples, there is a growing body of evidence that supports the use of digital technologies in measuring cognitive decline. For instance, systematic reviews on virtual reality have concluded that these technologies may assess crucial aspects such as spatial navigation and memory impairments [48, 49], which are often early signs of cognitive decline in at-risk populations [50, 51]. However, challenges related to achieving a balanced difficulty of the tests exist [49]. Our study mirrored these observations; specifically, we had to exclude data from Altoida for mild AD participants, who faced difficulties with fine motor tasks like drawing a circle on a smartphone, indicating even greater challenges when engaging with the more complex tasks offered by the application.

Another significant insight from our study is the effectiveness of the A-iADL questionnaire across various contexts. Although it was completed by the caregiver in this study, its potential for use in remote locations highlights its promise as a time-efficient and cost-effective supplement to traditional clinical evaluations, similar to other promising RMTs. These tools are widely accessible and could help identify and monitor functional decline in individuals with AD.

Recognizing AD patients in the pre-dementia phase is particularly important, as it may allow for early interventions with treatments like Leqembi or Kisunla, which may slow disease progression and enhance patient outcomes. Furthermore, RMTs can contribute significantly to clinical trials, aiding in diagnosis, patient stratification, and outcome assessments. Overall, the capacity of these technologies to improve the detection of earlier stages of Alzheimer’s Disease holds substantial potential, which could dramatically influence disease management strategies.

## Conclusion

The present study suggests that remote measurement technologies hold promise for detecting Alzheimer’s disease in its prodromal stage. While not discriminative enough for preclinical AD detection, the Altoida app, Mezurio app, Fitbit, and A-iADL questionnaire demonstrated potential for identifying individuals at the prodromal and mild-to-moderate AD stages. Although our study had limitations, these results show the potential of RMTs to enhance early AD detection. RMTs offer significant advantages – they are cost-effective, convenient, and accessible for at-home use. The ability to detect AD at earlier stages could enable earlier interventions and improve outcomes for patients, making the potential impact of RMTs on managing this debilitating disease significant. Overall, our findings highlight the importance of continued research and development in the field of RMTs for detecting AD and improving patient outcomes.

## Supplementary Information

Supplementary Material 1.

## Data Availability

The datasets analyzed during the current study are available from the corresponding author upon reasonable request. Furthermore, we make our code publicly available on https://github.com/SCAI-BIO/radar-ad-rmt-analysis.

## Abbreviations

AD: Alzheimer’s disease
ANCOVA: Analysis of Covariance
AUPR: Area Under the Precision-Recall Curve
AUROC: Area Under the Receiver Operating Characteristic
BMI: Body-Mass-Index
DNS: Digital Neuro Signature
FDS: Functional Domain Score
ML: Machine Learning
NCV: Nested Cross-Validation
REM: Rapid Eye Movement
RMT: Remote Monitoring Technology
SHAP: Shapley additive explanations
