# Supplementary material for: RADAR-AD: assessment of multiple remote monitoring technologies for early detection of Alzheimer’s disease
Source: Alzheimers Res Ther. 2025 Jan 27;17:29. doi: 10.1186/s13195-025-01675-0 (PMC11771057; doi:10.1186/s13195-025-01675-0)
Supplement: Supplementary file 1 — Supplementary Material 1. [file 13195_2025_1675_MOESM1_ESM.pdf]

Appendix A Additional Dataset Information

A.1 RADAR-AD Recruitment Process

Supplementary Figure A.1 illustrates the recruitment process. Initially, the memory clinics screened for AD biomarkers, MMSE, and CDR availability. After this screening, they contacted potential participants to explain the study details and the reasons for their eligibility. They also ensured that none of the exclusion criteria were met before proceeding. Participants who provided consent were then included in the study.

For more information on the RADAR-AD study and the recruitment process, please refer to the study protocol (https://www.radar-ad.org/our-research/project-deliverables) and other RADAR-AD publications (Muurling et al., 2021; Owens et al., 2020; Muurling et al., 2024).

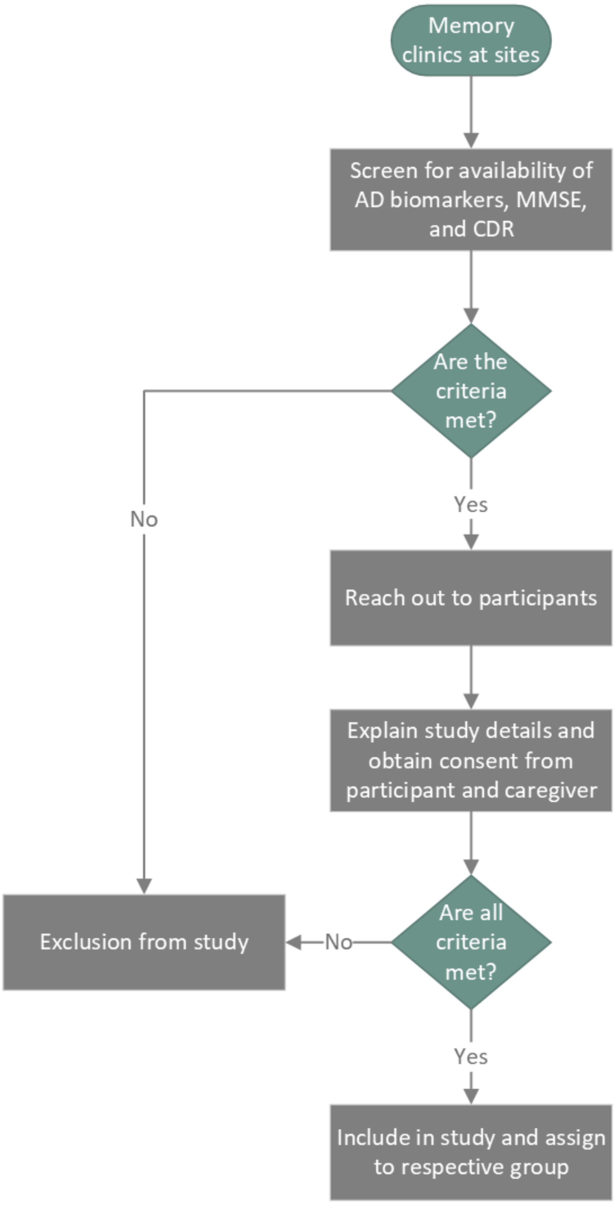

Supplementary Figure A.1: Schematic illustration of the RADAR-AD recruitment process

A.2 Analysis of Potentially Confounding Variables

In the Results section, it is demonstrated that the overall study groups are comparable in terms of demographic characteristics, with no significant differences observed in age, sex, years of education, and BMI. The distributions of these variables are illustrated in Supplementary Figure A.2. Additionally, Supplementary Table A.1 provides a detailed account of participant recruitment numbers, categorized by site and season, for each group.

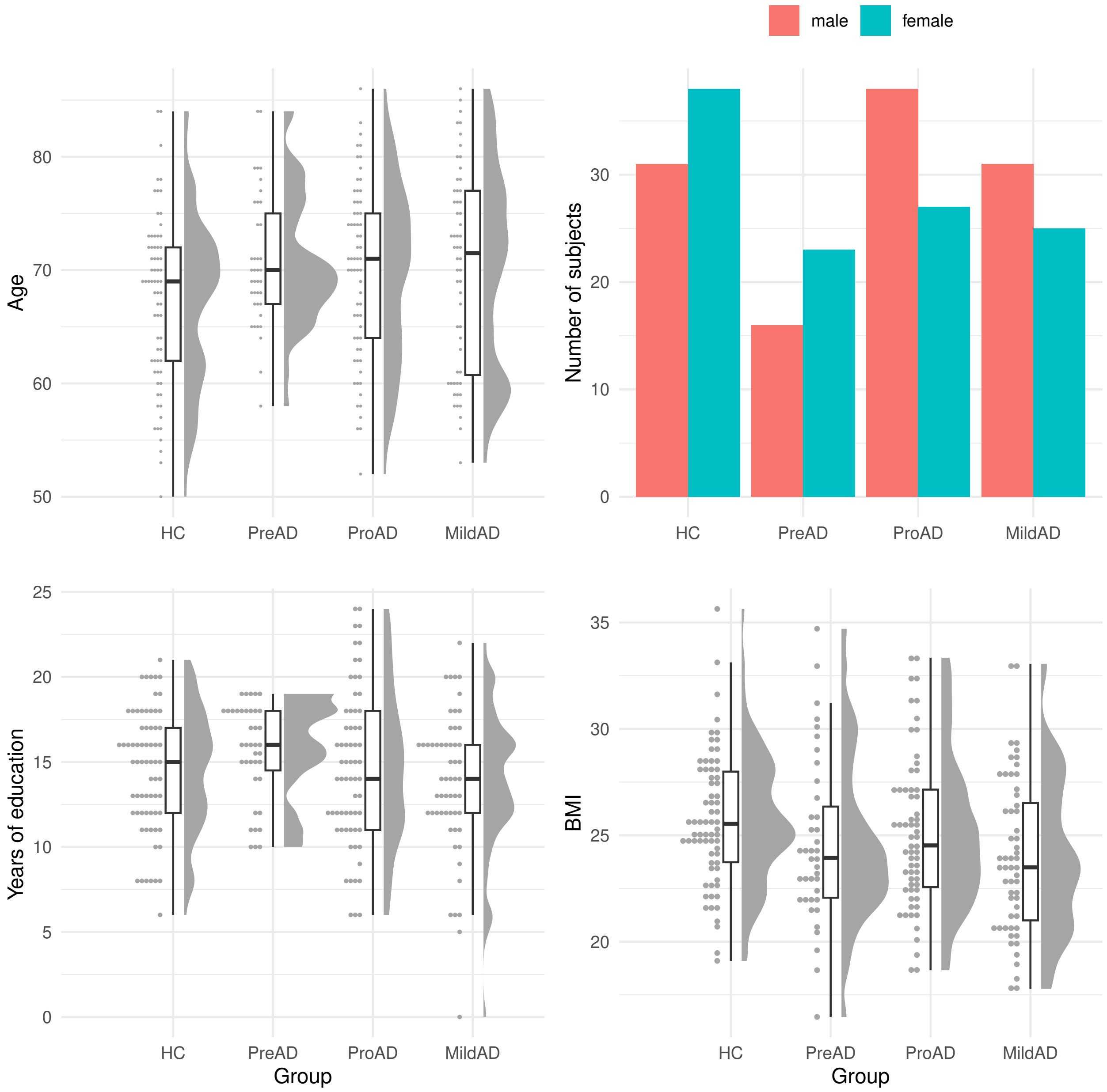

Supplementary Figure A.2: **Demographic and Social Characteristics of the Four Study Groups.** The box plots represent age, education years, and BMI distribution, while the bar graph illustrates the number of female and male participants across groups.

To further assess potential confounding variables, we conducted statistical tests per subset to evaluate whether any variables (age, sex, education years, site, season, and BMI) showed a statistically significant association

Supplementary Table A.1: **Study Characteristics of the Four Study Groups.** This table presents the distribution of participants across different sites and the season during which they were recruited for each study group.

		HC	PreAD	ProAD	MildAD
**Site**	Amsterdam	20	14	12	15
	Barcelona	8	3	6	3
	Brescia	6	0	4	1
	Bucharest	0	1	1	0
	Geneva	2	5	3	2
	Lisbon	7	0	8	5
	Ljubljana	4	2	2	2
	London	5	0	4	8
	Mannheim	0	1	5	3
	Oxford	6	3	2	0
	Stavanger	5	7	5	8
	Stockholm	0	3	7	1
	Thessaloniki	6	0	6	8
**Season at recruitment**	Autumn	15	16	13	17
	Spring	28	7	18	10
	Summer	16	10	20	17
	Winter	10	6	14	12

with the study groups. Categorical variables such as site, season, and sex were analyzed using the χ^2-test. In contrast, continuous variables were assessed using ANOVA or the Kruskal-Wallis test, depending on normality as determined by the Shapiro-Wilk test. P-values were adjusted for multiple comparisons using the Holm correction method. The results, presented in Table A.2a, indicate that the site was the only variable with significant results for the Altoida subset. As a confounder affects both the predictors and the dependent variable, we also examined the impact of these variables on the predictors. For cases where both the predictor and potentially confounding variables were continuous, we conducted Pearson correlation analysis in addition to ANOVA or the Kruskal-Wallis test. As before, significant results were adjusted for multiple testing. Table A.2b details these findings, revealing significant results for site, education years, and age. None of the variables appeared in both tables for a subset, indicating that none are confounding variables in this study.

A.3 Analysis of RMT-Data Availability

As mentioned in the main text and shown in Table 2, we did not obtain data from all RMTs for all study participants. This is because participants did not use the device/application or because the data itself was excluded in the initial data curation step. Supplementary Figure A.3 displays UpSet plots illustrating the overlap in participant counts between different data sources for different disease stages (a-d) and overall (e). As shown in Figure A.3e, only a limited number of participants (N=74) had complete data from all sources. This limitation is consistent across all disease stages. To mitigate this issue, we imputed missing values in our ML-based approach by fitting a KNN imputer to the training data and applying it to the test set, as described in the Methods section. However, it is crucial to acknowledge that this imputation step could potentially compromise the performance of individual classifiers.

Supplementary Table (A.2) **Analysis of Potential Confounding Variables.** The tables below offer a comprehensive statistical evaluation of potential confounding variables concerning study groups and features. They include test statistics, adjusted p-values, and effect sizes, specifying the statistical tests used. For χ^2 tests, Cramer's V is reported, and for Pearson correlation, the correlation coefficient is provided.

RMT	Variable	Test	Statistic	Adjusted p-value	Effect size
Altoida (CDS)	site	χ^2	67.7352	0.0448	0.409 (0.400, 0.551)

(a) Statistical Association Between Study Groups and Potential Confounders

RMT	Variable	Feature	Test	Statistic	Adjusted p-value	Effect size
FDS	site	self care	χ^2	768.5135	0.0000	0.529 (0.514, 0.608)
Axivity	site	acc-Weekday-avg	χ^2	1216.2946	0.0000	0.727 (0.648, 0.786)
Axivity	site	sedentary-overall-hour	χ^2	1288.1413	0.0000	0.748 (0.665, 0.804)
Axivity	age	acc-overall-avg	Pearson	-0.4264	0.0000	None
Fitbit	age	steps 17:00:00	Pearson	-0.3984	0.0000	None
Fitbit	age	steps 18:00:00	Pearson	-0.3793	0.0000	None
Axivity	age	acc-hourOfWeekday-17-avg	Pearson	-0.4214	0.0000	None
Axivity	age	acc-Weekend-avg	Pearson	-0.3799	0.0001	None
Axivity	age	acc-hourOfWeekday-18-avg	Pearson	-0.3875	0.0001	None
Fitbit	age	steps 20:00:00	Pearson	-0.3558	0.0002	None
FDS	education years	dysnomia	Pearson	0.3409	0.0003	None
Fitbit	age	light pct	Pearson	0.3565	0.0005	None
Axivity	age	acc-hourOfWeekday-12-avg	Pearson	-0.3548	0.0009	None
Fitbit	age	REM pct	Pearson	-0.3466	0.0010	None
Axivity	age	acc-hourOfWeekday-19-avg	Pearson	-0.3543	0.0010	None
Axivity	age	acc-hourOfWeekday-20-avg	Pearson	-0.3520	0.0012	None
Fitbit	age	steps 19:00:00	Pearson	-0.3342	0.0012	None
Axivity	age	acc-hourOfWeekday-16-avg	Pearson	-0.3469	0.0017	None
Fitbit	age	deep pct	Pearson	-0.3319	0.0033	None
Fitbit	age	steps 16:00:00	Pearson	-0.3109	0.0070	None
Axivity	age	light-hourOfWeekday-18-avg	Pearson	-0.3281	0.0071	None
Physilog (TUG)	site	NGaitCycles	χ^2	217.0723	0.0092	0.345 (0.319, 0.448)
Axivity	age	acc-overall-sd	Pearson	-0.3225	0.0106	None
FDS	site	motivation	χ^2	1101.5485	0.0110	0.678 (0.634, 0.754)
Axivity	education years	sleep-hourOfWeekday-16-avg	Pearson	-0.3218	0.0111	None
Axivity	age	sedentary-overall-avg	Pearson	0.3195	0.0131	None
Axivity	age	sedentary-hourOfWeekday-12-avg	Pearson	0.3193	0.0133	None
Axivity	site	sleep-hourOfWeekday-5-avg	χ^2	720.9144	0.0165	0.559 (0.555, 0.691)
Fitbit	site	hypersomnia	χ^2	541.9480	0.0196	0.475 (0.345, 0.516)
Axivity	age	acc-hourOfWeekday-21-avg	Pearson	-0.3069	0.0311	None
Fitbit	age	dailyMeanRem-Hours	Pearson	-0.2935	0.0336	None
Axivity	age	light-hourOfWeekday-17-avg	Pearson	-0.3009	0.0465	None
Axivity	education years	sleep-hourOfWeekday-17-avg	Pearson	-0.3002	0.0486	None

(b) Impact of Potential Confounders on Features

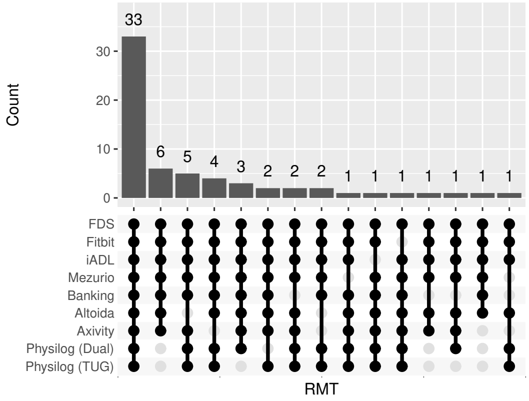

(a) Overlap between RMTs for healthy controls

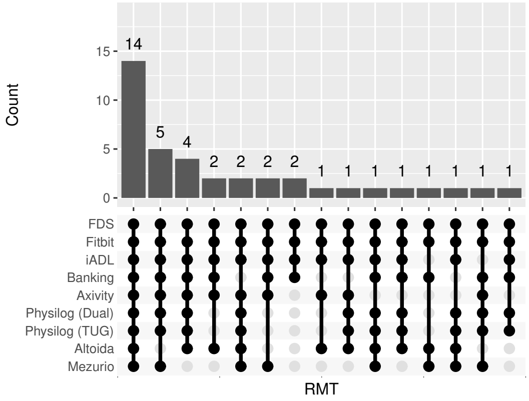

(b) Overlap between RMTs for preclinical AD participants

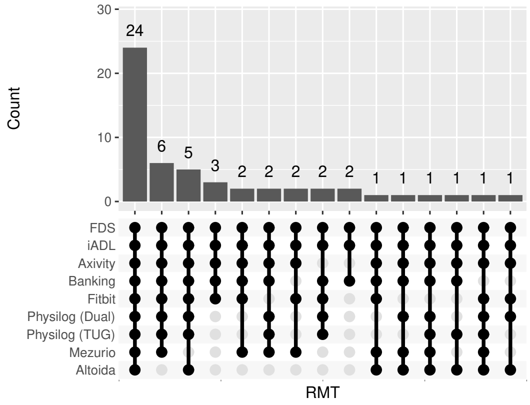

(c) Overlap between RMTs for prodromal AD participants

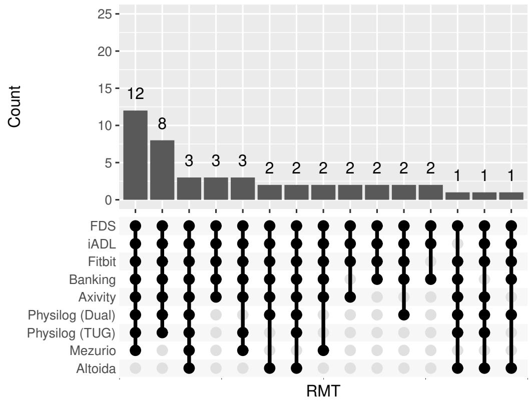

(d) Overlap between RMTs for mild-to-moderate AD participants

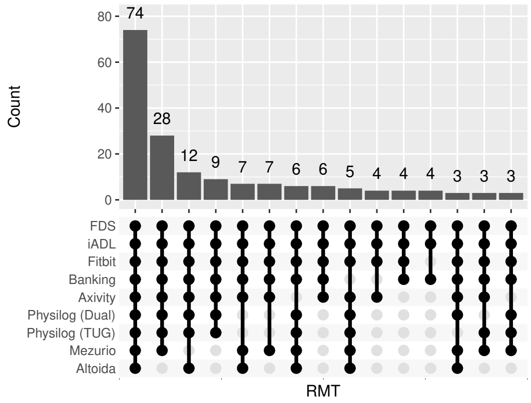

(e) Overlap between RMTs amongst all groups

Supplementary Figure A.3: **Visualizing RMT Data Overlap with Upset Plots.** The plot features a bar chart on top, quantifying the extent of each intersection, and a matrix plot below, where dots signify the data sources contributing to each intersection. Intersections are ranked by frequency and only the top 15 are displayed for clarity.

A.4 Features available per RMT

Supplementary Table A.3 shows a list of all available features for each of the six RMTs. Besides the feature name, a short description of its meaning is provided.

Supplementary Table A.3: **Table of Available Features for Each RMT:** The table below comprises two primary attributes: the feature name and a brief description. In the *Feature* column, we present the unique identifier for each feature, while in the *Description* column, we offer a concise outline of what each feature represents.

RMT	Feature	Description
Altoida	PerceptualMotorCoordination	Motor coordination in response to perceived input.
Altoida	ComplexAttention	The capacity to choose what to pay attention to and what to ignore.
Altoida	CognitiveProcessingSpeed	The speed and accuracy of information processing.
Altoida	Inhibition	The ability to tune out stimuli that are irrelevant to the task.
Altoida	Flexibility	The ability to switch between thinking about two different concepts.
Altoida	VisualPerception	The visual search speed, visual perception and efficiency.
Altoida	Planning	The process of thinking about the activities required to achieve a desired goal.
Altoida	ProspectiveMemory	The ability to remember to carry out intended actions in the future.
Altoida	SpatialMemory	The ability to recognize items that previously appeared in physical space.
Altoida	FineMotorSkills	The ability to perform tasks that require fine motor skills.
Altoida	Gait	A score for the quality of walking.
Altoida	DNS	Primary score for recognizing mild cognitive impairment (MCI) and Alzheimer's disease (AD)
Altoida	CN vs CI probability 0	Raw classification score used for the DNS score.
Axivity	acc-overall-avg	Average acceleration magnitude in mg (miligravity) over the complete study length.
Axivity	acc-overall-sd	Standard deviation of acceleration magnitude in mg (miligravity) over the complete study length.
Axivity	wearTime-overall(days)	Total wear time of both Axivity devices in days.
Axivity	sedentary-overall-avg	Average sedentary time: Average fraction of time spent in sedentary activity over the study length.
Axivity	sedentary-overall-sd	SD sedentary time: Standard deviation of the fraction of time spent in sedentary activity over the study length.
Axivity	light-overall-avg	Average light activity time: Average fraction of time spent in light activity over the study length.
Axivity	light-overall-sd	SD light activity time: Standard deviation of the fraction of time spent in light activity over the study length.
Axivity	MVPA-overall-avg	Average MVPA time: Average fraction of time spent in moderate-to-vigorous physical activity (MVPA) over the study length.
Axivity	MVPA-overall-sd	SD MVPA time: Standard deviation of the fraction of time spent in moderate-to-vigorous physical activity (MVPA) over the study length.
Axivity	sleep-overall-avg	Average sleep time: Average fraction of time spent sleeping over the study length.
Axivity	sleep-overall-sd	SD sleep time: Standard deviation of the fraction of time spent sleeping over the study length.
Axivity	nonWearTime-overall(days)	Total non-wear time: Total non-wear time of both Axivity devices in days.
Axivity	acc-Weekday-avg	Average weekday acceleration magnitude: Average acceleration magnitude over the study length, considering weekdays only.
Axivity	acc-Weekend-avg	Average weekend acceleration magnitude: Average acceleration magnitude over the study length, considering weekend days only.
Axivity	MVPA-Weekday-avg	Average weekday MVPA time: Average fraction of time spent in MVPA over the study length, considering weekdays only.

Continued on next page

RMT	Feature	Description
Axivity	MVPA-Weekend-avg	Average weekend MVPA time: Average fraction of time spent in MVPA over the study length, considering weekend days only.
Axivity	light-Weekday-avg	Average weekday light activity time: Average fraction of time spent in light activity over the study length, considering weekdays only.
Axivity	light-Weekend-avg	Average weekend light activity time: Average fraction of time spent in light activity over the study length, considering weekend days only.
Axivity	sedentary-Weekday-avg	Average weekday sedentary time: Average fraction of time spent in sedentary activity over the study length, considering weekdays only.
Axivity	sedentary-Weekend-avg	Average weekend sedentary time: Average fraction of time spent in sedentary activity over the study length, considering weekend days only.
Axivity	sleep-Weekday-avg	Average weekday sleep time: Average fraction of time spent sleeping over the study length, considering weekdays only.
Axivity	sleep-Weekend-avg	Average weekend sleep time: Average fraction of time spent sleeping over the study length, considering weekend days only.
Axivity	wear-Weekday-avg	Average weekday wear time: Average wear time over the study length, considering weekdays only.
Axivity	wear-Weekend-avg	Average weekend wear time: Average wear time over the study length, considering weekend days only.
Axivity	acc-hourOfWeekday-0-avg	Average acceleration magnitude in mg on weekdays from 12:00AM - 12:59AM
Axivity	acc-hourOfWeekday-1-avg	Average acceleration magnitude in mg on weekdays from 1:00AM - 1:59AM
Axivity	acc-hourOfWeekday-2-avg	Average acceleration magnitude in mg on weekdays from 2:00AM - 2:59AM
Axivity	acc-hourOfWeekday-3-avg	Average acceleration magnitude in mg on weekdays from 3:00AM - 3:59AM
Axivity	acc-hourOfWeekday-4-avg	Average acceleration magnitude in mg on weekdays from 4:00AM - 4:59AM
Axivity	acc-hourOfWeekday-5-avg	Average acceleration magnitude in mg on weekdays from 5:00AM - 5:59AM
Axivity	acc-hourOfWeekday-6-avg	Average acceleration magnitude in mg on weekdays from 6:00AM - 6:59AM
Axivity	acc-hourOfWeekday-7-avg	Average acceleration magnitude in mg on weekdays from 7:00AM - 7:59AM
Axivity	acc-hourOfWeekday-8-avg	Average acceleration magnitude in mg on weekdays from 8:00AM - 8:59AM
Axivity	acc-hourOfWeekday-9-avg	Average acceleration magnitude in mg on weekdays from 9:00AM - 9:59AM
Axivity	acc-hourOfWeekday-10-avg	Average acceleration magnitude in mg on weekdays from 10:00AM - 10:59AM
Axivity	acc-hourOfWeekday-11-avg	Average acceleration magnitude in mg on weekdays from 11:00AM - 11:59AM
Axivity	acc-hourOfWeekday-12-avg	Average acceleration magnitude in mg on weekdays from 12:00PM - 12:59PM
Axivity	acc-hourOfWeekday-13-avg	Average acceleration magnitude in mg on weekdays from 1:00PM - 1:59PM
Axivity	acc-hourOfWeekday-14-avg	Average acceleration magnitude in mg on weekdays from 2:00PM - 2:59PM
Axivity	acc-hourOfWeekday-15-avg	Average acceleration magnitude in mg on weekdays from 3:00PM - 3:59PM
Axivity	acc-hourOfWeekday-16-avg	Average acceleration magnitude in mg on weekdays from 4:00PM - 4:59PM
Axivity	acc-hourOfWeekday-17-avg	Average acceleration magnitude in mg on weekdays from 5:00PM - 5:59PM
Axivity	acc-hourOfWeekday-18-avg	Average acceleration magnitude in mg on weekdays from 6:00PM - 6:59PM
Axivity	acc-hourOfWeekday-19-avg	Average acceleration magnitude in mg on weekdays from 7:00PM - 7:59PM
Axivity	acc-hourOfWeekday-20-avg	Average acceleration magnitude in mg on weekdays from 8:00PM - 8:59PM
Axivity	acc-hourOfWeekday-21-avg	Average acceleration magnitude in mg on weekdays from 9:00PM - 9:59PM
Axivity	acc-hourOfWeekday-22-avg	Average acceleration magnitude in mg on weekdays from 10:00PM - 10:59PM
Axivity	acc-hourOfWeekday-23-avg	Average acceleration magnitude in mg on weekdays from 11:00PM - 11:59PM

Continued on next page

RMT	Feature	Description
Axivity	light-hourOfWeekday-0-avg	Proportion of light intensity physical activity tasks from 0:00 AM to 0:59 AM on weekdays.
Axivity	light-hourOfWeekday-1-avg	Proportion of light intensity physical activity tasks from 1:00 AM to 1:59 AM on weekdays.
Axivity	light-hourOfWeekday-2-avg	Proportion of light intensity physical activity tasks from 2:00 AM to 2:59 AM on weekdays.
Axivity	light-hourOfWeekday-3-avg	Proportion of light intensity physical activity tasks from 3:00 AM to 3:59 AM on weekdays.
Axivity	light-hourOfWeekday-4-avg	Proportion of light intensity physical activity tasks from 4:00 AM to 4:59 AM on weekdays.
Axivity	light-hourOfWeekday-5-avg	Proportion of light intensity physical activity tasks from 5:00 AM to 5:59 AM on weekdays.
Axivity	light-hourOfWeekday-6-avg	Proportion of light intensity physical activity tasks from 6:00 AM to 6:59 AM on weekdays.
Axivity	light-hourOfWeekday-7-avg	Proportion of light intensity physical activity tasks from 7:00 AM to 7:59 AM on weekdays.
Axivity	light-hourOfWeekday-8-avg	Proportion of light intensity physical activity tasks from 8:00 AM to 8:59 AM on weekdays.
Axivity	light-hourOfWeekday-9-avg	Proportion of light intensity physical activity tasks from 9:00 AM to 9:59 AM on weekdays.
Axivity	light-hourOfWeekday-10-avg	Proportion of light intensity physical activity tasks from 10:00 AM to 10:59 AM on weekdays.
Axivity	light-hourOfWeekday-11-avg	Proportion of light intensity physical activity tasks from 11:00 AM to 11:59 AM on weekdays.
Axivity	light-hourOfWeekday-12-avg	Proportion of light intensity physical activity tasks from 12:00 PM to 12:59 PM on weekdays.
Axivity	light-hourOfWeekday-13-avg	Proportion of light intensity physical activity tasks from 1:00 PM to 1:59 PM on weekdays.
Axivity	light-hourOfWeekday-14-avg	Proportion of light intensity physical activity tasks from 2:00 PM to 2:59 PM on weekdays.
Axivity	light-hourOfWeekday-15-avg	Proportion of light intensity physical activity tasks from 3:00 PM to 3:59 PM on weekdays.
Axivity	light-hourOfWeekday-16-avg	Proportion of light intensity physical activity tasks from 4:00 PM to 4:59 PM on weekdays.
Axivity	light-hourOfWeekday-17-avg	Proportion of light intensity physical activity tasks from 5:00 PM to 5:59 PM on weekdays.
Axivity	light-hourOfWeekday-18-avg	Proportion of light intensity physical activity tasks from 6:00 PM to 6:59 PM on weekdays.
Axivity	light-hourOfWeekday-19-avg	Proportion of light intensity physical activity tasks from 7:00 PM to 7:59 PM on weekdays.
Axivity	light-hourOfWeekday-20-avg	Proportion of light intensity physical activity tasks from 8:00 PM to 8:59 PM on weekdays.
Axivity	light-hourOfWeekday-21-avg	Proportion of light intensity physical activity tasks from 9:00 PM to 9:59 PM on weekdays.
Axivity	light-hourOfWeekday-22-avg	Proportion of light intensity physical activity tasks from 10:00 PM to 10:59 PM on weekdays.
Axivity	light-hourOfWeekday-23-avg	Proportion of light intensity physical activity tasks from 11:00 PM to 11:59 PM on weekdays.
Axivity	sedentary-hourOfWeekday-0-avg	Proportion of sedentary behavior from 12:00 AM to 12:59 AM on weekdays.
Axivity	sedentary-hourOfWeekday-1-avg	Proportion of sedentary behavior from 1:00 AM to 1:59 AM on weekdays.
Axivity	sedentary-hourOfWeekday-2-avg	Proportion of sedentary behavior from 2:00 AM to 2:59 AM on weekdays.
Axivity	sedentary-hourOfWeekday-3-avg	Proportion of sedentary behavior from 3:00 AM to 3:59 AM on weekdays.
Axivity	sedentary-hourOfWeekday-4-avg	Proportion of sedentary behavior from 4:00 AM to 4:59 AM on weekdays.
Axivity	sedentary-hourOfWeekday-5-avg	Proportion of sedentary behavior from 5:00 AM to 5:59 AM on weekdays.
Axivity	sedentary-hourOfWeekday-6-avg	Proportion of sedentary behavior from 6:00 AM to 6:59 AM on weekdays.
Axivity	sedentary-hourOfWeekday-7-avg	Proportion of sedentary behavior from 7:00 AM to 7:59 AM on weekdays.
Axivity	sedentary-hourOfWeekday-8-avg	Proportion of sedentary behavior from 8:00 AM to 8:59 AM on weekdays.
Axivity	sedentary-hourOfWeekday-9-avg	Proportion of sedentary behavior from 9:00 AM to 9:59 AM on weekdays.
Axivity	sedentary-hourOfWeekday-10-avg	Proportion of sedentary behavior from 10:00 AM to 10:59 AM on weekdays.
Axivity	sedentary-hourOfWeekday-11-avg	Proportion of sedentary behavior from 11:00 AM to 11:59 AM on weekdays.
Axivity	sedentary-hourOfWeekday-12-avg	Proportion of sedentary behavior from 12:00 PM to 12:59 PM on weekdays.

Continued on next page

RMT	Feature	Description
Axivity	sedentary-hourOfWeekday-13-avg	Proportion of sedentary behavior from 1:00 PM to 1:59 PM on weekdays.
Axivity	sedentary-hourOfWeekday-14-avg	Proportion of sedentary behavior from 2:00 PM to 2:59 PM on weekdays.
Axivity	sedentary-hourOfWeekday-15-avg	Proportion of sedentary behavior from 3:00 PM to 3:59 PM on weekdays.
Axivity	sedentary-hourOfWeekday-16-avg	Proportion of sedentary behavior from 4:00 PM to 4:59 PM on weekdays.
Axivity	sedentary-hourOfWeekday-17-avg	Proportion of sedentary behavior from 5:00 PM to 5:59 PM on weekdays.
Axivity	sedentary-hourOfWeekday-18-avg	Proportion of sedentary behavior from 6:00 PM to 6:59 PM on weekdays.
Axivity	sedentary-hourOfWeekday-19-avg	Proportion of sedentary behavior from 7:00 PM to 7:59 PM on weekdays.
Axivity	sedentary-hourOfWeekday-20-avg	Proportion of sedentary behavior from 8:00 PM to 8:59 PM on weekdays.
Axivity	sedentary-hourOfWeekday-21-avg	Proportion of sedentary behavior from 9:00 PM to 9:59 PM on weekdays.
Axivity	sedentary-hourOfWeekday-22-avg	Proportion of sedentary behavior from 10:00 PM to 10:59 PM on weekdays.
Axivity	sedentary-hourOfWeekday-23-avg	Proportion of sedentary behavior from 11:00 PM to 11:59 PM on weekdays.
Axivity	sleep-hourOfWeekday-0-avg	Proportion of sleep from 12:00 AM to 12:59 AM on weekdays.
Axivity	sleep-hourOfWeekday-1-avg	Proportion of sleep from 1:00 AM to 1:59 AM on weekdays.
Axivity	sleep-hourOfWeekday-2-avg	Proportion of sleep from 2:00 AM to 2:59 AM on weekdays.
Axivity	sleep-hourOfWeekday-3-avg	Proportion of sleep from 3:00 AM to 3:59 AM on weekdays.
Axivity	sleep-hourOfWeekday-4-avg	Proportion of sleep from 4:00 AM to 4:59 AM on weekdays.
Axivity	sleep-hourOfWeekday-5-avg	Proportion of sleep from 5:00 AM to 5:59 AM on weekdays.
Axivity	sleep-hourOfWeekday-6-avg	Proportion of sleep from 6:00 AM to 6:59 AM on weekdays.
Axivity	sleep-hourOfWeekday-7-avg	Proportion of sleep from 7:00 AM to 7:59 AM on weekdays.
Axivity	sleep-hourOfWeekday-8-avg	Proportion of sleep from 8:00 AM to 8:59 AM on weekdays.
Axivity	sleep-hourOfWeekday-9-avg	Proportion of sleep from 9:00 AM to 9:59 AM on weekdays.
Axivity	sleep-hourOfWeekday-10-avg	Proportion of sleep from 10:00 AM to 10:59 AM on weekdays.
Axivity	sleep-hourOfWeekday-11-avg	Proportion of sleep from 11:00 AM to 11:59 AM on weekdays.
Axivity	sleep-hourOfWeekday-12-avg	Proportion of sleep from 12:00 PM to 12:59 PM on weekdays.
Axivity	sleep-hourOfWeekday-13-avg	Proportion of sleep from 1:00 PM to 1:59 PM on weekdays.
Axivity	sleep-hourOfWeekday-14-avg	Proportion of sleep from 2:00 PM to 2:59 PM on weekdays.
Axivity	sleep-hourOfWeekday-15-avg	Proportion of sleep from 3:00 PM to 3:59 PM on weekdays.
Axivity	sleep-hourOfWeekday-16-avg	Proportion of sleep from 4:00 PM to 4:59 PM on weekdays.
Axivity	sleep-hourOfWeekday-17-avg	Proportion of sleep from 5:00 PM to 5:59 PM on weekdays.
Axivity	sleep-hourOfWeekday-18-avg	Proportion of sleep from 6:00 PM to 6:59 PM on weekdays.
Axivity	sleep-hourOfWeekday-19-avg	Proportion of sleep from 7:00 PM to 7:59 PM on weekdays.
Axivity	sleep-hourOfWeekday-20-avg	Proportion of sleep from 8:00 PM to 8:59 PM on weekdays.
Axivity	sleep-hourOfWeekday-21-avg	Proportion of sleep from 9:00 PM to 9:59 PM on weekdays.
Axivity	sleep-hourOfWeekday-22-avg	Proportion of sleep from 10:00 PM to 10:59 PM on weekdays.
Axivity	sleep-hourOfWeekday-23-avg	Proportion of sleep from 11:00 PM to 11:59 PM on weekdays.
Axivity	MVPA-hourOfWeekday-0-avg	Proportion of moderate intensity physical activity from 12:00 AM to 12:59 AM on weekdays.
Axivity	MVPA-hourOfWeekday-1-avg	Proportion of moderate intensity physical activity from 1:00 AM to 1:59 AM on weekdays.

Continued on next page

RMT	Feature	Description
Axivity	MVPA-hourOfWeekday-2-avg	Proportion of moderate intensity physical activity from 2:00 AM to 2:59 AM on weekdays.
Axivity	MVPA-hourOfWeekday-3-avg	Proportion of moderate intensity physical activity from 3:00 AM to 3:59 AM on weekdays.
Axivity	MVPA-hourOfWeekday-4-avg	Proportion of moderate intensity physical activity from 4:00 AM to 4:59 AM on weekdays.
Axivity	MVPA-hourOfWeekday-5-avg	Proportion of moderate intensity physical activity from 5:00 AM to 5:59 AM on weekdays.
Axivity	MVPA-hourOfWeekday-6-avg	Proportion of moderate intensity physical activity from 6:00 AM to 6:59 AM on weekdays.
Axivity	MVPA-hourOfWeekday-7-avg	Proportion of moderate intensity physical activity from 7:00 AM to 7:59 AM on weekdays.
Axivity	MVPA-hourOfWeekday-8-avg	Proportion of moderate intensity physical activity from 8:00 AM to 8:59 AM on weekdays.
Axivity	MVPA-hourOfWeekday-9-avg	Proportion of moderate intensity physical activity from 9:00 AM to 9:59 AM on weekdays.
Axivity	MVPA-hourOfWeekday-10-avg	Proportion of moderate intensity physical activity from 10:00 AM to 10:59 AM on weekdays.
Axivity	MVPA-hourOfWeekday-11-avg	Proportion of moderate intensity physical activity from 11:00 AM to 11:59 AM on weekdays.
Axivity	MVPA-hourOfWeekday-12-avg	Proportion of moderate intensity physical activity from 12:00 PM to 12:59 PM on weekdays.
Axivity	MVPA-hourOfWeekday-13-avg	Proportion of moderate intensity physical activity from 1:00 PM to 1:59 PM on weekdays.
Axivity	MVPA-hourOfWeekday-14-avg	Proportion of moderate intensity physical activity from 2:00 PM to 2:59 PM on weekdays.
Axivity	MVPA-hourOfWeekday-15-avg	Proportion of moderate intensity physical activity from 3:00 PM to 3:59 PM on weekdays.
Axivity	MVPA-hourOfWeekday-16-avg	Proportion of moderate intensity physical activity from 4:00 PM to 4:59 PM on weekdays.
Axivity	MVPA-hourOfWeekday-17-avg	Proportion of moderate intensity physical activity from 5:00 PM to 5:59 PM on weekdays.
Axivity	MVPA-hourOfWeekday-18-avg	Proportion of moderate intensity physical activity from 6:00 PM to 6:59 PM on weekdays.
Axivity	MVPA-hourOfWeekday-19-avg	Proportion of moderate intensity physical activity from 7:00 PM to 7:59 PM on weekdays.
Axivity	MVPA-hourOfWeekday-20-avg	Proportion of moderate intensity physical activity from 8:00 PM to 8:59 PM on weekdays.
Axivity	MVPA-hourOfWeekday-21-avg	Proportion of moderate intensity physical activity from 9:00 PM to 9:59 PM on weekdays.
Axivity	MVPA-hourOfWeekday-22-avg	Proportion of moderate intensity physical activity from 10:00 PM to 10:59 PM on weekdays.
Axivity	MVPA-hourOfWeekday-23-avg	Proportion of moderate intensity physical activity from 11:00 PM to 11:59 PM on weekdays.
Axivity	sedentary-overall-hour	Time in hours spent in sedentary activity, averaged over the complete study length (sedentary-overall-avg * 24).
Axivity	light-overall-hour	Time in hours spent in light activity, averaged over the complete study length (light-overall-avg * 24).
Axivity	MVPA-overall-hour	Time in hours spent in moderate intensity physical activity, averaged over the complete study length (MVPA-overall-avg * 24).
Axivity	sleep-overall-hour	Time in hours spent in sleep, averaged over the complete study length (sleep-overall-avg * 24).
Banking	Total Attempts BankApp count	Total number of attempts made by participants in the banking application. An attempt consists of three steps: entering PIN, entering amount, and pressing confirm.
Banking	SUM Duration Attempts BankApp msec	Sum of durations (in milliseconds) for all attempts made in the banking application. An attempt consists of three steps: entering PIN, entering amount, and pressing confirm.
Banking	AVG Duration Attempts BankApp msec	Average duration (in milliseconds) for attempts made in the banking application. An attempt consists of three steps: entering PIN, entering amount, and pressing confirm.
Banking	Correct Attempts BankApp count	Number (count) of correct attempts made in the banking application. An attempt consists of three steps: entering PIN, entering amount, and pressing confirm.
Banking	Wrong Attempts BankApp count	Number (count) of wrong attempts made in the banking application. An attempt consists of three steps: entering PIN, entering amount, and pressing confirm.

Continued on next page

RMT	Feature	Description
Banking	SUM Duration Correct Attempts BankApp msec	Sum of durations (in milliseconds) for all correct attempts made in the banking application. An attempt consists of three steps: entering PIN, entering amount, and pressing confirm.
Banking	AVG Duration Correct Attempts BankApp msec	Average duration (in milliseconds) for correct attempts made in the banking application. An attempt consists of three steps: entering PIN, entering amount, and pressing confirm.
Banking	SUM Duration Wrong Attempts BankApp msec	Sum of durations (in milliseconds) for all wrong attempts made in the banking application. An attempt consists of three steps: entering PIN, entering amount, and pressing confirm.
Banking	AVG Duration Wrong Attempts BankApp msec	Average duration (in milliseconds) for wrong attempts made in the banking application. An attempt consists of three steps: entering PIN, entering amount, and pressing confirm.
Banking	Total Attempts PIN count	Number (count) of attempts made for the step "Enter PIN" in the banking application.
Banking	SUM PIN Duration msec	Sum of durations (in milliseconds) for all attempts made for the step "Enter PIN" in the banking application.
Banking	AVG PIN Duration msec	Average duration (in milliseconds) for attempts made for the step "Enter PIN" in the banking application.
Banking	Correct PIN Attempts count	Number (count) of correct attempts made for the step "Enter PIN" in the banking application.
Banking	SUM Correct PIN Duration msec	Sum of durations (in milliseconds) for all correct attempts made for the step "Enter PIN" in the banking application.
Banking	AVG Correct PIN Duration msec	Average duration (in milliseconds) for correct attempts made for the step "Enter PIN" in the banking application.
Banking	Wrong PIN Attempts count	Number (count) of wrong attempts made for the step "Enter PIN" in the banking application.
Banking	SUM Wrong PIN Duration msec	Sum of durations (in milliseconds) for all wrong attempts made for the step "Enter PIN" in the banking application.
Banking	AVG Wrong PIN Duration msec	Average duration (in milliseconds) for wrong attempts made for the step "Enter PIN" in the banking application.
Banking	Total Attempts Amount count	Number (count) of attempts made for the step "Enter Amount" in the banking application.
Banking	SUM Amount Duration msec	Sum of durations (in milliseconds) for all attempts made for the step "Enter Amount" in the banking application.
Banking	AVG Amount Duration msec	Average duration (in milliseconds) for attempts made for the step "Enter Amount" in the banking application.
Banking	Correct Amount Attempts count	Number (count) of correct attempts made for the step "Enter Amount" in the banking application.
Banking	SUM Correct Amount Duration msec	Sum of durations (in milliseconds) for all correct attempts made for the step "Enter Amount" in the banking application.
Banking	AVG Correct Amount Duration msec	Average duration (in milliseconds) for correct attempts made for the step "Enter Amount" in the banking application.
Banking	Wrong Amount Attempts count	Number (count) of wrong attempts made for the step "Enter Amount" in the banking application.
Banking	SUM Wrong Amount Duration msec	Sum of durations (in milliseconds) for all wrong attempts made for the step "Enter Amount" in the banking application.
Banking	AVG Wrong Amount Duration msec	Average duration (in milliseconds) for wrong attempts made for the step "Enter Amount" in the banking application.
Banking	Total Confirm attempts count	Number (count) of attempts made for the step "Confirm" in the banking application.
Banking	Total Cancel attempts count	Number (count) of attempts made for the step "Cancel" in the banking application.
Banking	Total Correct Confirm attempts count	Number (count) of correct attempts made for the step "Confirm" in the banking application.
Banking	Total Wrong Confirm attempts count	Number (count) of wrong attempts made for the step "Confirm" in the banking application.

Continued on next page

RMT	Feature	Description
Banking	SUM Duration Correct Confirm msec	Sum of durations (in milliseconds) for all correct attempts made for the step "Confirm" in the banking application.
Banking	AVG Duration Correct Confirm msec	Average duration (in milliseconds) for correct attempts made for the step "Confirm" in the banking application.
Banking	SUM Duration Wrong Confirm msec	Sum of durations (in milliseconds) for all wrong attempts made for the step "Confirm" in the banking application.
Banking	AVG Duration Wrong Confirm msec	Average duration (in milliseconds) for wrong attempts made for the step "Confirm" in the banking application.
Banking	Duration Cancel msec	Duration (in milliseconds) of attempts made for the step "Cancel" in the banking application.
Fitbit	hr 00:00:00	Average heart rate data for each hour (12:00AM - 12:59AM)
Fitbit	hr 01:00:00	Average heart rate data for each hour (1:00AM - 1:59AM)
Fitbit	hr 02:00:00	Average heart rate data for each hour (2:00AM - 2:59AM)
Fitbit	hr 03:00:00	Average heart rate data for each hour (3:00AM - 3:59AM)
Fitbit	hr 04:00:00	Average heart rate data for each hour (4:00AM - 4:59AM)
Fitbit	hr 05:00:00	Average heart rate data for each hour (5:00AM - 5:59AM)
Fitbit	hr 06:00:00	Average heart rate data for each hour (6:00AM - 6:59AM)
Fitbit	hr 07:00:00	Average heart rate data for each hour (7:00AM - 7:59AM)
Fitbit	hr 08:00:00	Average heart rate data for each hour (8:00AM - 8:59AM)
Fitbit	hr 09:00:00	Average heart rate data for each hour (9:00AM - 9:59AM)
Fitbit	hr 10:00:00	Average heart rate data for each hour (10:00AM - 10:59AM)
Fitbit	hr 11:00:00	Average heart rate data for each hour (11:00AM - 11:59AM)
Fitbit	hr 12:00:00	Average heart rate data for each hour (12:00PM - 12:59PM)
Fitbit	hr 13:00:00	Average heart rate data for each hour (1:00PM - 1:59PM)
Fitbit	hr 14:00:00	Average heart rate data for each hour (2:00PM - 2:59PM)
Fitbit	hr 15:00:00	Average heart rate data for each hour (3:00PM - 3:59PM)
Fitbit	hr 16:00:00	Average heart rate data for each hour (4:00PM - 4:59PM)
Fitbit	hr 17:00:00	Average heart rate data for each hour (5:00PM - 5:59PM)
Fitbit	hr 18:00:00	Average heart rate data for each hour (6:00PM - 6:59PM)
Fitbit	hr 19:00:00	Average heart rate data for each hour (7:00PM - 7:59PM)
Fitbit	hr 20:00:00	Average heart rate data for each hour (8:00PM - 8:59PM)
Fitbit	hr 21:00:00	Average heart rate data for each hour (9:00PM - 9:59PM)
Fitbit	hr 22:00:00	Average heart rate data for each hour (10:00PM - 10:59PM)
Fitbit	hr 23:00:00	Average heart rate data for each hour (11:00PM - 11:59PM)
Fitbit	dailyMeanHeartRate	Average daily heart rate of the participant in beats per minute over the complete study length
Fitbit	dailyMaxHeartRate	Average maximum daily heart rate of the participant in beats per minute over the complete study length
Fitbit	dailyMinHeartRate	Average minimum daily heart rate of the participant in beats per minute over the complete study length
Fitbit	steps 00:00:00	Average step count per hour (12:00AM - 12:59AM)
Fitbit	steps 01:00:00	Average step count per hour (1:00AM - 1:59AM)
Fitbit	steps 02:00:00	Average step count per hour (2:00AM - 2:59AM)

Continued on next page

RMT	Feature	Description
Fitbit	steps 03:00:00	Average step count per hour (3:00AM - 3:59AM)
Fitbit	steps 04:00:00	Average step count per hour (4:00AM - 4:59AM)
Fitbit	steps 05:00:00	Average step count per hour (5:00AM - 5:59AM)
Fitbit	steps 06:00:00	Average step count per hour (6:00AM - 6:59AM)
Fitbit	steps 07:00:00	Average step count per hour (7:00AM - 7:59AM)
Fitbit	steps 08:00:00	Average step count per hour (8:00AM - 8:59AM)
Fitbit	steps 09:00:00	Average step count per hour (9:00AM - 9:59AM)
Fitbit	steps 10:00:00	Average step count per hour (10:00AM - 10:59AM)
Fitbit	steps 11:00:00	Average step count per hour (11:00AM - 11:59AM)
Fitbit	steps 12:00:00	Average step count per hour (12:00PM - 12:59PM)
Fitbit	steps 13:00:00	Average step count per hour (1:00PM - 1:59PM)
Fitbit	steps 14:00:00	Average step count per hour (2:00PM - 2:59PM)
Fitbit	steps 15:00:00	Average step count per hour (3:00PM - 3:59PM)
Fitbit	steps 16:00:00	Average step count per hour (4:00PM - 4:59PM)
Fitbit	steps 17:00:00	Average step count per hour (5:00PM - 5:59PM)
Fitbit	steps 18:00:00	Average step count per hour (6:00PM - 6:59PM)
Fitbit	steps 19:00:00	Average step count per hour (7:00PM - 7:59PM)
Fitbit	steps 20:00:00	Average step count per hour (8:00PM - 8:59PM)
Fitbit	steps 21:00:00	Average step count per hour (9:00PM - 9:59PM)
Fitbit	steps 22:00:00	Average step count per hour (10:00PM - 10:59PM)
Fitbit	steps 23:00:00	Average step count per hour (11:00PM - 11:59PM)
Fitbit	dailyMeanSteps	Average daily step count over the complete study length
Fitbit	dailyMeanAsleepHours	Average duration of participant's daily sleep in hours, including all sleep stages (REM, Light, Deep) except for the 'Awake' stage, over the complete study length
Fitbit	dailyMeanAwakeHours	Average duration of participant's daily awake time in hours, over the complete study length, using the 'Awake' stage
Fitbit	dailyMeanBedtimeHours	Average duration of participant's daily bedtime in hours, over the complete study length
Fitbit	dailyMeanRemHours	Average duration of participant's daily REM sleep stage in hours, over the complete study length
Fitbit	wearTimeMinutes	Total minutes of wear time
Fitbit	wearTimePercentage	Percentage of wear time
Fitbit	total sleep time	Total sleep time
Fitbit	time in bed	Total time spent in bed
Fitbit	light pct	Percentage of time spent in the light sleep stage
Fitbit	deep pct	Percentage of time spent in the deep sleep stage
Fitbit	REM pct	Percentage of time spent in the REM sleep stage
Fitbit	awake pct	Percentage of time spent awake
Fitbit	NREM pct	Percentage of time spent in non-REM sleep stages (light + deep sleep)
Fitbit	sleep onset	Average sleep onset time
Fitbit	sleep offset	Average sleep offset time
Fitbit	rem latency	Average latency to enter REM sleep
Fitbit	sleep efficiency	Average sleep efficiency
Fitbit	awakenings	Average number of awakenings
Fitbit	insomnia	Insomnia indicator
Fitbit	hypersomnia	Hypersomnia indicator
Mezurio	audio number of syllables	The number of syllables in the recording.
Mezurio	audio number of pauses	The number of pauses in the recording.
Mezurio	audio average pause duration	The average pause duration in the recording (in seconds).
Mezurio	audio file duration in second	The duration of the recording (in seconds).
Mezurio	audio total speech duration	The total speech duration in the recording (in seconds).
Mezurio	audio speaking rate	The speaking rate calculated from the number of syllables.
Mezurio	audio articulation rate	The articulation rate calculated from the number of syllables.
Mezurio	audio average syllable duration	The average duration of a syllable (in seconds).
Mezurio	audio rms energy	The root mean square (RMS) energy of the recording.
Mezurio	audio hesitation ratio	The hesitation ratio calculated as the pause duration divided by the audio duration.
Mezurio	ost F0semitoneFrom27 5Hz sma3nz amean	Average pitch in semitones relative to 27.5Hz.
Mezurio	ost F0semitoneFrom27 5Hz sma3nz stddevNorm	Standard deviation of pitch in semitones relative to 27.5Hz (normalized).
Mezurio	ost F0semitoneFrom27 5Hz sma3nz percentile20 0	20th percentile of pitch in semitones relative to 27.5Hz.
Mezurio	ost F0semitoneFrom27 5Hz sma3nz percentile50 0	50th percentile (median) of pitch in semitones relative to 27.5Hz.

Continued on next page

RMT	Feature	Description
Mezurio	ost F0semitoneFrom27 5Hz sma3nz percentile80 0	80th percentile of pitch in semitones relative to 27.5Hz.
Mezurio	ost F0semitoneFrom27 5Hz sma3nz pctlrange0 2	Range between the 0th and 2nd percentiles of pitch in semitones relative to 27.5Hz.
Mezurio	ost F0semitoneFrom27 5Hz sma3nz meanRisingSlope	Mean slope of pitch rising intervals in semitones relative to 27.5Hz.
Mezurio	ost F0semitoneFrom27 5Hz sma3nz stddevRisingSlope	Standard deviation of slope of pitch rising intervals in semitones relative to 27.5Hz.
Mezurio	ost F0semitoneFrom27 5Hz sma3nz meanFallingSlope	Mean slope of pitch falling intervals in semitones relative to 27.5Hz.
Mezurio	ost F0semitoneFrom27 5Hz sma3nz stddevFallingSlope	Standard deviation of slope of pitch falling intervals in semitones relative to 27.5Hz.
Mezurio	ost loudness sma3 amean	Average loudness.
Mezurio	ost loudness sma3 stddevNorm	Standard deviation of loudness (normalized).
Mezurio	ost loudness sma3 percentile20 0	20th percentile of loudness.
Mezurio	ost loudness sma3 percentile50 0	50th percentile (median) of loudness.
Mezurio	ost loudness sma3 percentile80 0	80th percentile of loudness.
Mezurio	ost loudness sma3 pctlrange0 2	Range between the 0th and 2nd percentiles of loudness.
Mezurio	ost loudness sma3 meanRisingSlope	Mean slope of loudness rising intervals.
Mezurio	ost loudness sma3 stddevRisingSlope	Standard deviation of slope of loudness rising intervals.
Mezurio	ost loudness sma3 meanFallingSlope	Mean slope of loudness falling intervals.
Mezurio	ost loudness sma3 stddevFallingSlope	Standard deviation of slope of loudness falling intervals.
Mezurio	ost spectralFlux sma3 amean	Average spectral flux.
Mezurio	ost spectralFlux sma3 stddevNorm	Standard deviation of spectral flux (normalized).
Mezurio	ost mfcc1 sma3 amean	Average Mel-frequency cepstral coefficient 1.
Mezurio	ost mfcc1 sma3 stddevNorm	Standard deviation of Mel-frequency cepstral coefficient 1 (normalized).
Mezurio	ost mfcc2 sma3 amean	Average Mel-frequency cepstral coefficient 2.
Mezurio	ost mfcc2 sma3 stddevNorm	Standard deviation of Mel-frequency cepstral coefficient 2 (normalized).
Mezurio	ost mfcc3 sma3 amean	Average Mel-frequency cepstral coefficient 3.
Mezurio	ost mfcc3 sma3 stddevNorm	Standard deviation of Mel-frequency cepstral coefficient 3 (normalized).
Mezurio	ost mfcc4 sma3 amean	Average Mel-frequency cepstral coefficient 4.
Mezurio	ost mfcc4 sma3 stddevNorm	Standard deviation of Mel-frequency cepstral coefficient 4 (normalized).
Mezurio	ost jitterLocal sma3nz amean	Average local jitter (normalized).
Mezurio	ost jitterLocal sma3nz stddevNorm	Standard deviation of local jitter (normalized).
Mezurio	ost shimmerLocaldB sma3nz amean	Average local shimmer in dB (normalized).
Mezurio	ost shimmerLocaldB sma3nz stddevNorm	Standard deviation of local shimmer in dB (normalized).
Mezurio	ost HNRdBACF sma3nz amean	Average harmonic-to-noise ratio in dB (ACF method).
Mezurio	ost HNRdBACF sma3nz stddevNorm	Standard deviation of harmonic-to-noise ratio in dB (ACF method, normalized).
Mezurio	ost logRelF0 H1 H2 sma3nz amean	Average log ratio of energy in harmonic components (H1 and H2) to the total energy (normalized).
Mezurio	ost logRelF0 H1 H2 sma3nz stddevNorm	Standard deviation of log ratio of energy in harmonic components (H1 and H2) to the total energy (normalized).
Mezurio	ost logRelF0 H1 A3 sma3nz amean	Average log ratio of energy in harmonic component (H1) to energy in the first formant (A3) (normalized).
Mezurio	ost logRelF0 H1 A3 sma3nz stddevNorm	Standard deviation of log ratio of energy in harmonic component (H1) to energy in the first formant (A3) (normalized).
Mezurio	ost F1frequency sma3nz amean	Average frequency of the first formant.
Mezurio	ost F1frequency sma3nz stddevNorm	Standard deviation of the frequency of the first formant (normalized).
Mezurio	ost F1amplitudeLogRelF0 sma3nz amean	Average log ratio of energy in the first formant (F1) to the total energy (normalized).
Mezurio	ost F1amplitudeLogRelF0 sma3nz stddevNorm	Standard deviation of log ratio of energy in the first formant (F1) to the total energy (normalized).
Mezurio	ost F2frequency sma3nz amean	Average frequency of the second formant.
Mezurio	ost F2frequency sma3nz stddevNorm	Standard deviation of the frequency of the second formant (normalized).
Mezurio	ost F2amplitudeLogRelF0 sma3nz amean	Average log ratio of energy in the second formant (F2) to the total energy (normalized).
Mezurio	ost F2amplitudeLogRelF0 sma3nz stddevNorm	Standard deviation of log ratio of energy in the second formant (F2) to the total energy (normalized).
Mezurio	ost F3frequency sma3nz amean	Average frequency of the third formant.

Continued on next page

RMT	Feature	Description
Mezurio	ost F3frequency sma3nz stddevNorm	Standard deviation of the frequency of the third formant (normalized).
Mezurio	ost F3amplitudeLogRelF0 sma3nz amean	Average log ratio of energy in the third formant (F3) to the total energy (normalized).
Mezurio	ost F3amplitudeLogRelF0 sma3nz stddevNorm	Standard deviation of log ratio of energy in the third formant (F3) to the total energy (normalized).
Mezurio	ost alphaRatioV sma3nz amean	Average ratio of alpha-band energy to vocal tract energy.
Mezurio	ost alphaRatioV sma3nz stddevNorm	Standard deviation of the ratio of alpha-band energy to vocal tract energy (normalized).
Mezurio	ost hammarbergIndexV sma3nz amean	Average Hammarberg index of the vocal tract.
Mezurio	ost hammarbergIndexV sma3nz stddevNorm	Standard deviation of Hammarberg index of the vocal tract (normalized).
Mezurio	ost slopeV0 500 sma3nz amean	Average slope of the spectral envelope between 0 and 500 Hz.
Mezurio	ost slopeV0 500 sma3nz stddevNorm	Standard deviation of the slope of the spectral envelope between 0 and 500 Hz (normalized).
Mezurio	ost slopeV500 1500 sma3nz amean	Average slope of the spectral envelope between 500 and 1500 Hz.
Mezurio	ost slopeV500 1500 sma3nz stddevNorm	Standard deviation of the slope of the spectral envelope between 500 and 1500 Hz (normalized).
Mezurio	ost spectralFluxV sma3nz amean	Average spectral flux of the vocal tract.
Mezurio	ost spectralFluxV sma3nz stddevNorm	Standard deviation of spectral flux of the vocal tract (normalized).
Mezurio	ost mfcc1V sma3nz amean	Average Mel-frequency cepstral coefficient 1 of the vocal tract.
Mezurio	ost mfcc1V sma3nz stddevNorm	Standard deviation of Mel-frequency cepstral coefficient 1 of the vocal tract (normalized).
Mezurio	ost mfcc2V sma3nz amean	Average Mel-frequency cepstral coefficient 2 of the vocal tract.
Mezurio	ost mfcc2V sma3nz stddevNorm	Standard deviation of Mel-frequency cepstral coefficient 2 of the vocal tract (normalized).
Mezurio	ost mfcc3V sma3nz amean	Average Mel-frequency cepstral coefficient 3 of the vocal tract.
Mezurio	ost mfcc3V sma3nz stddevNorm	Standard deviation of Mel-frequency cepstral coefficient 3 of the vocal tract (normalized).
Mezurio	ost mfcc4V sma3nz amean	Average Mel-frequency cepstral coefficient 4 of the vocal tract.
Mezurio	ost mfcc4V sma3nz stddevNorm	Standard deviation of Mel-frequency cepstral coefficient 4 of the vocal tract (normalized).
Mezurio	ost alphaRatioUV sma3nz amean	Average ratio of alpha-band energy to unvoiced vocal tract energy.
Mezurio	ost hammarbergIndexUV sma3nz amean	Average Hammarberg index of the unvoiced vocal tract.
Mezurio	ost slopeUV0 500 sma3nz amean	Average slope of the spectral envelope between 0 and 500 Hz for unvoiced segments.
Mezurio	ost slopeUV500 1500 sma3nz amean	Average slope of the spectral envelope between 500 and 1500 Hz for unvoiced segments.
Mezurio	ost spectralFluxUV sma3nz amean	Average spectral flux of unvoiced segments.
Mezurio	ost loudnessPeaksPerSec	Number of loudness peaks per second.
Mezurio	ost VoicedSegmentsPerSec	Number of voiced segments per second.
Mezurio	ost MeanVoicedSegmentLengthSec	Mean duration of voiced segments in seconds.
Mezurio	ost StddevVoicedSegmentLengthSec	Standard deviation of duration of voiced segments in seconds.
Mezurio	ost MeanUnvoicedSegmentLength	Mean duration of unvoiced segments.
Mezurio	ost StddevUnvoicedSegmentLength	Standard deviation of duration of unvoiced segments.
Mezurio	ost equivalentSoundLevel dBp	Equivalent sound level in dB.
Physilog GaitUp Dual	avg peakswing	Dual Tasking Effect[1] - peak swing: Maximum angular velocity during swing phase.
Physilog GaitUp Dual	cv peakswing	Dual Tasking Effect[1] - peak swing: Coefficient of variation of angular velocity during swing phase.
Physilog GaitUp Dual	avg speed	Dual Tasking Effect[1] - gait speed: Average speed for each gait cycle.
Physilog GaitUp Dual	cv speed	Dual Tasking Effect[1] - gait speed: Coefficient of variation of gait speed.
Physilog GaitUp Dual	avg LDr	Dual Tasking Effect[1] - loading rate: Initial phase of stance where the foot rotates to reach foot flat.

Continued on next page

RMT	Feature	Description
Physilog GaitUp Dual	cv LDr	Dual Tasking Effect[1] - loading rate: Coefficient of variation of loading rate.
Physilog GaitUp Dual	avg DS	Dual Tasking Effect[1] - double support: Percentage of gait cycle when both feet are touching the floor.
Physilog GaitUp Dual	cv DS	Dual Tasking Effect[1] - double support: Coefficient of variation of double support.
Physilog GaitUp Dual	avg swing	Dual Tasking Effect[1] - swing phase: Percentage of gait cycle when the foot is not touching the ground.
Physilog GaitUp Dual	cv swing	Dual Tasking Effect[1] - swing phase: Coefficient of variation of swing phase.
Physilog GaitUp Dual	avg PUr	Dual Tasking Effect[1] - push off rate: Final phase of stance when the heel is raised while the toe still touches the ground.
Physilog GaitUp Dual	cv PUr	Dual Tasking Effect[1] - push off rate: Coefficient of variation of push off rate.
Physilog GaitUp Dual	avg stance	Dual Tasking Effect[1] - stance phase: Percentage of gait cycle when the foot is touching the ground.
Physilog GaitUp Dual	cv stance	Dual Tasking Effect[1] - stance phase: Coefficient of variation of stance phase.
Physilog GaitUp Dual	avg HSP	Dual Tasking Effect[1] - heel strike angle: Angle between the foot and the ground at heel strike.
Physilog GaitUp Dual	cv HSP	Dual Tasking Effect[1] - heel strike angle: Coefficient of variation of heel strike angle.
Physilog GaitUp Dual	avg FFr	Dual Tasking Effect[1] - foot flat rate: Percentage of stance time when the foot is entirely touching the floor.
Physilog GaitUp Dual	cv FFr	Dual Tasking Effect[1] - foot flat rate: Coefficient of variation of foot flat rate.
Physilog GaitUp Dual	avg PathLength	Dual Tasking Effect[1] - 3D path length: Real path of the foot in 3D space during one cycle.
Physilog GaitUp Dual	cv PathLength	Dual Tasking Effect[1] - 3D path length: Coefficient of variation of 3D path length.
Physilog GaitUp Dual	avg cadence	Dual Tasking Effect[1] - cadence: Number of steps per minute.
Physilog GaitUp Dual	cv cadence	Dual Tasking Effect[1] - cadence: Coefficient of variation of cadence.
Physilog GaitUp Dual	avg TOP	Dual Tasking Effect[1] - lift-off angle: Angle of the foot at the end of the push phase, just at take-off.
Physilog GaitUp Dual	cv TOP	Dual Tasking Effect[1] - lift-off angle: Coefficient of variation of lift-off angle.
Physilog GaitUp Dual	avg slength	Dual Tasking Effect[1] - stride length: Horizontal distance from one heel strike to the next of the same foot.
Physilog GaitUp Dual	cv slength	Dual Tasking Effect[1] - stride length: Coefficient of variation of stride length.
Physilog GaitUp Dual	avg gct	Dual Tasking Effect[1] - gait cycle time: Time from one heel strike to the next of the same foot.

Continued on next page

RMT	Feature	Description
Physilog GaitUp Dual	cv gct	Dual Tasking Effect[1] - gait cycle time: Coefficient of variation of gait cycle time.
Physilog GaitUp TUG	total time	Total TUG test time: Time needed to perform the TUG test (seconds).
Physilog GaitUp TUG	sist duration	Sit-to-stand duration: Duration of sit-to-stand movement (seconds).
Physilog GaitUp TUG	sist angle range	Sit-to-stand angle range: Range of angles during sit-to-stand movement (degrees).
Physilog GaitUp TUG	turn duration	Turn duration: Duration of turning movement (seconds).
Physilog GaitUp TUG	turnsi duration	Turn-to-sit duration: Duration of turn-to-sit movement (seconds).
Physilog GaitUp TUG	NGaitCycles	Number of gait cycles.
Physilog GaitUp TUG	cadence	Cadence: Cycles per minute.
Physilog GaitUp TUG	gaitspeed	Gait speed: Meters per second.

[1] The Dual Tasking Effect (DTE) is defined by $\mathrm{DTE}_x = 100 \times \frac{x_{\mathrm{DUAL}} - x_{\mathrm{SINGLE}}}{x_{\mathrm{SINGLE}}}$.

Appendix B Additional Methodology

B.1 Calculation of Functional Domain Scores

Throughout our experiments, we used Functional Domain Scores (FDS) to compare the performance of conventional clinical assessments compared to the RMTs we assessed. We calculated different scores for each functional domain by using the results from the assessments performed for each participant in the RADAR-AD study cohort. In the following, we list the relevant instruments and their references. For a complete overview of all assessments, we refer to the research protocol available on the RADAR-AD website[1].

- Alzheimer's Disease Cooperative Study—Activities of Daily Living scale (ADCS-ADL) (Pedregosa et al., 2011; Galasko et al., 1997)
- Amsterdam-iADL (A-iADL)[2] (Sikkes et al., 2012, 2013)
- 15 item Boston Naming Test (BNT) (Mack et al., 1992)
- Epworth Sleepiness scale (ESS) (Johns, 1991)
- Euro Quality of Life (EQ-5D) (Hurst et al., 1997)
- Everyday Cognition (ECOG) scale (Farias et al., 2008)
- Geriatric Depression Scale (GDSS) (Sheikh and Yesavage, 1986)
- Neuro Psychiatric Inventory (NPI) (Kaufer et al., 2000)
- Pittsburgh Sleep Quality Index (PSI) (Buysse et al., 1989)
- Rey complex figure (RCF) (Cherrier et al., 1999)
- Smartphone proficiency (SP): a test of a patient's smartphone proficiency by a) making a phonecall, b) sending a text message, c) sending a whatsapp message, and d) reading the last received E-mail. Participants are assessed on the following levels: dexterity, self-assurance, and efficiency.
- Socal Functioning Scale (SFS) (Birchwood et al., 1990)
- Verbal fluency (VF) (Henry et al., 2004)
- Word list learning (WLL) (Heun et al., 1998)

The functional domains were assigned to the individual instruments by experts from the RADAR-AD consortium based on their assessment. In several cases, multiple instruments were related to the same functional domain. In this case, z-scores were calculated for each, and then the average of these z-scores was used as the representative metric for the corresponding cognitive domain. Supplementary Table B.1 shows how each functional domain score is calculated.

[1]https://www.radar-ad.org/our-research/project-deliverables
[2]https://www.alzheimercentrum.nl/professionals/amsterdam-iadl/

Supplementary Table B.1: Scores included in the Functional Domain Scores.

**Functional Domain**	**Instrument**	**Calculation**
Difficulties at work	A-iADL	Question 20 about difficulty at work
Spatial navigation & memory	Word list learning	Score for recall
	A-iADL	Score for question 29
	Rey complex figure	Score for recall
	ECOG	Score for memory divided by memory completed
	ECOG	Score for visual spatial divided by visual spatial completed
Planning skills & memory required for task completion	ECOG	Score for planning divided by planning completed
Managing finance	A-iADL	Sum of scores for questions 11, 14, 15, 16, and 17
Self-care	EQ-5D	Score for EQ5D Question 2
	A-iADL	Score for question R30
	ADCS-ADL	10 minus (score for Question 5 + score for Q6A + score for Q6B)
Self-management, e.g., running errands & shopping	A-iADL	Sum of scores for Questions 1, 2, 3, 4, 5, and 6
	ECOG	Score for organization divided by organization completed
Sleep quality & circadian rhythms	PSQI	Sum of scores for seven PSQI components
	ESS	Sum of scores for eight components (sitting reading + watching tv + sitting inactive + passenger + lying down + sitting talking + sitting after lunch + car stopped)
	NPI	Product of NPI nighttime frequency and NPI nighttime severity scores
Use of Technology/Devices	Smartphone Proficiency	60 - (phonecall dex + phonecall selfass + phonecall eff + sms dex + sms selfass + sms eff + whatsapp dex + whatsapp selfass + whatsapp eff + email dex + email selfass + email eff)
	A-iADL	Sum of Questions 7, 8, 9, 10, 22, 23, 24, 25, and 26
	ADCS-ADL	9 - (sum of Questions 7a and 23b)
Dysnomia, Word Finding Difficulties	Verbal fluency	letter1 score + letter2 score + letter3 score + animals
	Boston Naming Test	Total score
Gait	EQ-5D	Question 1
Driving Difficulties	A-iADL	Questions 27 and 28
Interpersonal Interaction and Prosocial Behaviors	SFS	(15 - withdrawal) / 15 + (30 - (sum of interpersonal scores)) / 30 + (78 -prosocial) / 78
Motivation, Signs of Apathy or Withdrawal	GDSS	total score
	NPI	Product of NPI apathy frequency and NPI apathy severity scores
	SFS	15 - withdrawal

The table shows the respective cognitive domain, instrument, and how each subscore was calculated. If multiple subscores exist per cognitive domain, the mean of their z-scores is used as a measure.

Appendix C Additional Results

C.1 Results of the ANCOVA analysis

Supplementary Table C.1 shows the detailed results for each feature. In contrast to Figure 2, this table shows the results even if the ANCOVA was insignificant. Furthermore, it is indicated whether the data has been log-transformed or not and effect size estimates as well as their 95 % confidence interval are provided.

Supplementary Table C.1: The table below presents a detailed statistical analysis of the RMTs features. Column one denotes the RMT, with its corresponding feature given in column two. Column three indicates whether data has been log-transformed or not. The remaining columns show the results of the ANCOVA and statistical results of the Tukey HSD tests (conducted when ANCOVA was significant). In each case, we provide the test statistic and the corrected p-value.

RMT	Feature	Log	Ancova	HC vs. PreAD	HC vs. ProAD	HC vs. MildAD	PreAD vs. ProAD	PreAD vs. MildAD	ProAD vs. MildAD
Altoida	CognitiveProcessingSpeed	×	F=20.224<br>η^2=0.27 [0.16, 1.00]<br>p<0.001	2.23 [-2.38,6.83]<br>d=0.22 [-0.23, 0.67]<br>p=1.0	10.98 [6.80,15.16]<br>d=1.13 [0.69, 1.57]<br>p<0.001	–	8.76 [3.79,13.72]<br>d=1.17 [0.63, 1.69]<br>p=0.013	–	–
Altoida	ComplexAttention	×	F=21.146<br>η^2=0.28 [0.16, 1.00]<br>p<0.001	1.11 [-3.41,5.62]<br>d=0.12 [-0.34, 0.57]<br>p=1.0	10.77 [6.67,14.87]<br>d=1.14 [0.70, 1.58]<br>p<0.001	–	9.66 [4.79,14.54]<br>d=1.26 [0.72, 1.79]<br>p=0.002	–	–
Altoida	DNS	✓	F=24.184<br>η^2=0.31 [0.19, 1.00]<br>p<0.001	0.23 [-0.05,0.50]<br>d=0.47 [0.01, 0.92]<br>p=1.0	0.74 [0.48,0.99]<br>d=1.34 [0.88, 1.79]<br>p<0.001	–	0.51 [0.21,0.81]<br>d=0.88 [0.36, 1.38]<br>p=0.023	–	–
Altoida	DNS @home1	✓	F=5.056<br>η^2=0.13 [0.02, 1.00]<br>p=0.009	0.17 [-0.19,0.54]<br>d=0.33 [-0.22, 0.87]<br>p=1.0	0.45 [0.11,0.80]<br>d=0.80 [0.26, 1.32]<br>p=0.402	–	0.28 [-0.12,0.69]<br>d=0.48 [-0.14, 1.10]<br>p=1.0	–	–
Altoida	DNS @home2	×	F=3.157<br>η^2=0.10 [0.00, 1.00]<br>p=0.05	6.86 [-6.35,20.07]<br>d=0.37 [-0.21, 0.95]<br>p=1.0	13.29 [0.33,26.24]<br>d=0.74 [0.15, 1.31]<br>p=1.0	–	6.42 [-8.83,21.67]<br>d=0.34 [-0.33, 1.00]<br>p=1.0	–	–
Altoida	DNS @home3	✓	F=3.726<br>η^2=0.12 [0.01, 1.00]<br>p=0.031	0.15 [-0.14,0.43]<br>d=0.41 [-0.19, 1.00]<br>p=1.0	0.33 [0.03,0.62]<br>d=0.88 [0.24, 1.50]<br>p=1.0	–	0.18 [-0.16,0.52]<br>d=0.41 [-0.31, 1.12]<br>p=1.0	–	–
Altoida	FineMotorSkills	×	F=0.214<br>η^2=0.00 [0.00, 1.00]<br>p=0.808	–	–	–	–	–	–
Altoida	Flexibility	✓	F=0.137<br>η^2=0.00 [0.00, 1.00]<br>p=0.872	–	–	–	–	–	–
Altoida	Gait	×	F=9.613<br>η^2=0.15 [0.05, 1.00]<br>p<0.001	5.50 [0.29,10.70]<br>d=0.56 [0.09, 1.01]<br>p=1.0	8.47 [3.75,13.19]<br>d=0.88 [0.44, 1.30]<br>p=0.009	–	2.97 [-2.64,8.59]<br>d=0.35 [-0.14, 0.84]<br>p=1.0	–	–
Altoida	Inhibition	✓	F=1.75<br>η^2=0.03 [0.00, 1.00]<br>p=0.179	–	–	–	–	–	–
Altoida	PerceptualMotorCoordination	×	F=9.571<br>η^2=0.15 [0.05, 1.00]<br>p<0.001	2.16 [-0.76,5.08]<br>d=0.39 [-0.07, 0.85]<br>p=1.0	4.88 [2.23,7.53]<br>d=0.85 [0.42, 1.27]<br>p=0.006	–	2.72 [-0.43,5.87]<br>d=0.53 [0.03, 1.03]<br>p=1.0	–	–
Altoida	Planning	×	F=19.431<br>η^2=0.26 [0.15, 1.00]<br>p<0.001	2.65 [-2.35,7.64]<br>d=0.26 [-0.19, 0.71]<br>p=1.0	11.72 [7.19,16.26]<br>d=1.16 [0.71, 1.60]<br>p<0.001	–	9.08 [3.69,14.47]<br>d=1.06 [0.54, 1.58]<br>p=0.026	–	–
Altoida	ProspectiveMemory	✓	F=2.95<br>η^2=0.05 [0.00, 1.00]<br>p=0.057	–	–	–	–	–	–
Altoida	SpatialMemory	×	F=13.036<br>η^2=0.19 [0.09, 1.00]<br>p<0.001	3.51 [-1.52,8.55]<br>d=0.36 [-0.10, 0.82]<br>p=1.0	9.81 [5.24,14.38]<br>d=1.04 [0.60, 1.47]<br>p<0.001	–	6.30 [0.86,11.73]<br>d=0.73 [0.22, 1.23]<br>p=1.0	–	–
Altoida	VisualPerception	×	F=14.419<br>η^2=0.21 [0.10, 1.00]<br>p<0.001	1.97 [-3.57,7.50]<br>d=0.18 [-0.27, 0.63]<br>p=1.0	11.09 [6.07,16.12]<br>d=1.02 [0.58, 1.45]<br>p<0.001	–	9.12 [3.15,15.10]<br>d=0.96 [0.44, 1.47]<br>p=0.094	–	–
Axivity	MVPA.Weekday.avg	✓	F=1.417<br>η^2=0.02 [0.00, 1.00]<br>p=0.239	–	–	–	–	–	–
Axivity	MVPA.Weekend.avg	✓	F=2.67<br>η^2=0.04 [0.00, 1.00]<br>p=0.049	0.39 [-3.23,4.00]<br>d=0.06 [-0.37, 0.49]<br>p=1.0	1.98 [-1.09,5.04]<br>d=0.29 [-0.08, 0.65]<br>p=1.0	3.28 [0.00,6.56]<br>d=0.46 [0.07, 0.86]<br>p=1.0	1.59 [-2.07,5.25]<br>d=0.22 [-0.21, 0.66]<br>p=1.0	2.89 [-0.94,6.73]<br>d=0.39 [-0.07, 0.85]<br>p=1.0	1.31 [-2.02,4.63]<br>d=0.17 [-0.23, 0.56]<br>p=1.0
Axivity	MVPA.overall.avg	✓	F=2.455<br>η^2=0.04 [0.00, 1.00]<br>p=0.065	–	–	–	–	–	–

Continued on next page

RMT	Feature	Log	Ancova	HC vs. PreAD	HC vs. ProAD	HC vs. MildAD	PreAD vs. ProAD	PreAD vs. MildAD	ProAD vs. MildAD
Axivity	MVPA.overall.hour	✓	F=1.719<br>η^2=0.03 [0.00, 1.00]<br>p=0.165	–	–	–	–	–	–
Axivity	MVPA.overall.sd	×	F=1.256<br>η^2=0.02 [0.00, 1.00]<br>p=0.291	–	–	–	–	–	–
Axivity	acc.Weekday.avg	✓	F=0.828<br>η^2=0.01 [0.00, 1.00]<br>p=0.48	–	–	–	–	–	–
Axivity	acc.Weekend.avg	×	F=3.823<br>η^2=0.06 [0.01, 1.00]<br>p=0.011	3.89 [0.15,7.64]<br>d=0.55 [0.12, 0.99]<br>p=1.0	2.90 [-0.28,6.08]<br>d=0.41 [0.04, 0.78]<br>p=1.0	3.65 [0.26,7.05]<br>d=0.46 [0.07, 0.85]<br>p=0.947	-0.99 [-4.78,2.80]<br>d=-0.16 [-0.60, 0.27]<br>p=1.0	-0.24 [-4.21,3.74]<br>d=-0.03 [-0.49, 0.42]<br>p=1.0	0.75 [-2.69,4.20]<br>d=0.10 [-0.29, 0.50]<br>p=1.0
Axivity	acc.overall.avg	✓	F=5.308<br>η^2=0.08 [0.02, 1.00]<br>p=0.002	0.17 [0.02,0.33]<br>d=0.66 [0.22, 1.10]<br>p=1.0	0.15 [0.01,0.28]<br>d=0.50 [0.13, 0.87]<br>p=1.0	0.19 [0.05,0.33]<br>d=0.59 [0.19, 0.99]<br>p=0.162	-0.02 [-0.18,0.13]<br>d=-0.08 [-0.51, 0.35]<br>p=1.0	0.02 [-0.15,0.18]<br>d=0.06 [-0.40, 0.51]<br>p=1.0	0.04 [-0.10,0.19]<br>d=0.12 [-0.27, 0.52]<br>p=1.0
Axivity	acc.overall.sd	✓	F=5.506<br>η^2=0.09 [0.02, 1.00]<br>p=0.001	0.21 [0.02,0.40]<br>d=0.67 [0.23, 1.11]<br>p=1.0	0.20 [0.04,0.36]<br>d=0.56 [0.19, 0.93]<br>p=0.536	0.23 [0.05,0.40]<br>d=0.58 [0.18, 0.97]<br>p=0.211	-0.01 [-0.20,0.19]<br>d=-0.02 [-0.46, 0.41]<br>p=1.0	0.02 [-0.19,0.22]<br>d=0.04 [-0.41, 0.50]<br>p=1.0	0.03 [-0.15,0.20]<br>d=0.06 [-0.33, 0.46]<br>p=1.0
Axivity	light.Weekday.avg	✓	F=2.907<br>η^2=0.05 [0.00, 1.00]<br>p=0.036	0.82 [-2.30,3.94]<br>d=0.21 [-0.22, 0.64]<br>p=1.0	2.85 [0.20,5.50]<br>d=0.50 [0.13, 0.87]<br>p=1.0	2.02 [-0.81,4.85]<br>d=0.39 [0.00, 0.78]<br>p=1.0	2.03 [-1.13,5.19]<br>d=0.32 [-0.12, 0.75]<br>p=1.0	1.20 [-2.11,4.51]<br>d=0.20 [-0.26, 0.66]<br>p=1.0	-0.83 [-3.70,2.04]<br>d=-0.12 [-0.51, 0.28]<br>p=1.0
Axivity	light.Weekend.avg	×	F=5.847<br>η^2=0.09 [0.03, 1.00]<br>p<0.001	0.04 [0.00,0.09]<br>d=0.53 [0.09, 0.96]<br>p=1.0	0.04 [0.00,0.07]<br>d=0.43 [0.06, 0.80]<br>p=1.0	0.06 [0.02,0.10]<br>d=0.72 [0.32, 1.12]<br>p=0.022	-0.01 [-0.05,0.04]<br>d=-0.07 [-0.50, 0.36]<br>p=1.0	0.02 [-0.02,0.07]<br>d=0.28 [-0.17, 0.74]<br>p=1.0	0.03 [-0.01,0.07]<br>d=0.33 [-0.07, 0.73]<br>p=1.0
Axivity	light.overall.avg	×	F=6.466<br>η^2=0.10 [0.03, 1.00]<br>p<0.001	0.04 [0.00,0.08]<br>d=0.63 [0.19, 1.07]<br>p=1.0	0.04 [0.00,0.07]<br>d=0.53 [0.16, 0.90]<br>p=1.0	0.06 [0.02,0.10]<br>d=0.75 [0.34, 1.15]<br>p=0.015	0.00 [-0.04,0.04]<br>d=-0.03 [-0.46, 0.40]<br>p=1.0	0.02 [-0.02,0.06]<br>d=0.23 [-0.22, 0.69]<br>p=1.0	0.02 [-0.02,0.06]<br>d=0.25 [-0.15, 0.64]<br>p=1.0
Axivity	light.overall.hour	×	F=1.397<br>η^2=0.02 [0.00, 1.00]<br>p=0.245	–	–	–	–	–	–
Axivity	light.overall.sd	✓	F=7.841<br>η^2=0.12 [0.05, 1.00]<br>p<0.001	0.05 [-0.03,0.13]<br>d=0.49 [0.05, 0.92]<br>p=1.0	0.07 [0.00,0.14]<br>d=0.51 [0.13, 0.87]<br>p=1.0	0.13 [0.06,0.20]<br>d=0.82 [0.42, 1.23]<br>p=0.001	0.02 [-0.06,0.10]<br>d=0.14 [-0.30, 0.57]<br>p=1.0	0.08 [0.00,0.16]<br>d=0.47 [0.00, 0.93]<br>p=1.0	0.06 [-0.01,0.13]<br>d=0.33 [-0.07, 0.72]<br>p=1.0
Axivity	nonWearTime.overall.days.	✓	F=2.585<br>η^2=0.04 [0.00, 1.00]<br>p=0.055	–	–	–	–	–	–
Axivity	sedentary.Weekday.avg	×	F=0.849<br>η^2=0.01 [0.00, 1.00]<br>p=0.469	–	–	–	–	–	–
Axivity	sedentary.Weekend.avg	×	F=3.916<br>η^2=0.06 [0.01, 1.00]<br>p=0.01	-0.05 [-0.09,-0.01]<br>d=-0.64 [-1.07, -0.20]<br>p=1.0	-0.04 [-0.07,0.00]<br>d=-0.44 [-0.81, -0.07]<br>p=1.0	-0.04 [-0.08,0.00]<br>d=-0.46 [-0.86, -0.07]<br>p=1.0	0.01 [-0.03,0.06]<br>d=0.17 [-0.26, 0.61]<br>p=1.0	0.01 [-0.04,0.06]<br>d=0.11 [-0.34, 0.57]<br>p=1.0	0.00 [-0.04,0.04]<br>d=-0.04 [-0.44, 0.35]<br>p=1.0
Axivity	sedentary.overall.avg	×	F=3.953<br>η^2=0.06 [0.01, 1.00]<br>p=0.009	-0.04 [-0.08,0.00]<br>d=-0.60 [-1.04, -0.16]<br>p=1.0	-0.04 [-0.07,0.00]<br>d=-0.47 [-0.84, -0.10]<br>p=1.0	-0.04 [-0.08,0.00]<br>d=-0.47 [-0.86, -0.08]<br>p=1.0	0.01 [-0.04,0.05]<br>d=0.09 [-0.34, 0.52]<br>p=1.0	0.00 [-0.04,0.05]<br>d=0.05 [-0.41, 0.51]<br>p=1.0	0.00 [-0.04,0.04]<br>d=-0.03 [-0.43, 0.36]<br>p=1.0
Axivity	sedentary.overall.hour	✓	F=0.99<br>η^2=0.02 [0.00, 1.00]<br>p=0.399	–	–	–	–	–	–
Axivity	sedentary.overall.sd	✓	F=3.03<br>η^2=0.05 [0.00, 1.00]<br>p=0.031	-0.04 [-0.07,0.00]<br>d=-0.60 [-1.03, -0.16]<br>p=1.0	-0.02 [-0.05,0.01]<br>d=-0.29 [-0.65, 0.08]<br>p=1.0	-0.01 [-0.04,0.02]<br>d=-0.16 [-0.55, 0.23]<br>p=1.0	0.02 [-0.02,0.05]<br>d=0.29 [-0.15, 0.73]<br>p=1.0	0.03 [-0.01,0.06]<br>d=0.48 [0.02, 0.94]<br>p=1.0	0.01 [-0.02,0.04]<br>d=0.14 [-0.26, 0.53]<br>p=1.0
Axivity	sleep.Weekday.avg	×	F=1.258<br>η^2=0.02 [0.00, 1.00]<br>p=0.29	–	–	–	–	–	–
Axivity	sleep.Weekend.avg	✓	F=3.259<br>η^2=0.05 [0.00, 1.00]<br>p=0.023	0.00 [-0.08,0.08]<br>d=0.00 [-0.43, 0.42]<br>p=1.0	-0.02 [-0.09,0.05]<br>d=-0.15 [-0.52, 0.21]<br>p=1.0	-0.08 [-0.15,-0.01]<br>d=-0.53 [-0.92, -0.13]<br>p=0.85	-0.02 [-0.10,0.06]<br>d=-0.16 [-0.59, 0.28]<br>p=1.0	-0.08 [-0.16,0.00]<br>d=-0.53 [-0.99, -0.07]<br>p=1.0	-0.06 [-0.13,0.01]<br>d=-0.40 [-0.80, 0.00]<br>p=1.0

Continued on next page

RMT	Feature	Log	Ancova	HC vs. PreAD	HC vs. ProAD	HC vs. MildAD	PreAD vs. ProAD	PreAD vs. MildAD	ProAD vs. MildAD
Axivity	sleep.overall.avg	✓	F=2.385<br>η^2=0.04 [0.00, 1.00]<br>p=0.071	–	–	–	–	–	–
Axivity	sleep.overall.hour	✓	F=1.296<br>η^2=0.02 [0.00, 1.00]<br>p=0.278	–	–	–	–	–	–
Axivity	sleep.overall.sd	✓	F=0.186<br>η^2=0.00 [0.00, 1.00]<br>p=0.906	–	–	–	–	–	–
Axivity	wear.Weekday.avg	✓	F=0.723<br>η^2=0.01 [0.00, 1.00]<br>p=0.54	–	–	–	–	–	–
Axivity	wear.Weekend.avg	✓	F=0.591<br>η^2=0.01 [0.00, 1.00]<br>p=0.622	–	–	–	–	–	–
Axivity	wearTime.overall.days.	✓	F=1.4<br>η^2=0.02 [0.00, 1.00]<br>p=0.245	–	–	–	–	–	–
Banking	AVG Amount Duration msec	✓	F=17.196<br>η^2=0.22 [0.13, 1.00]<br>p<0.001	0.07 [-0.27,0.41]<br>d=0.12 [-0.30, 0.54]<br>p=1.0	-0.32 [-0.61,-0.03]<br>d=-0.55 [-0.91, -0.18]<br>p=1.0	-0.73 [-1.03,-0.43]<br>d=-1.06 [-1.46, -0.66]<br>p<0.001	-0.39 [-0.73,-0.05]<br>d=-0.66 [-1.09, -0.22]<br>p=1.0	-0.80 [-1.15,-0.45]<br>d=-1.10 [-1.56, -0.63]<br>p<0.001	-0.41 [-0.72,-0.10]<br>d=-0.58 [-0.97, -0.19]<br>p=0.248
Banking	AVG Correct Amount Duration msec	✓	F=16.925<br>η^2=0.22 [0.13, 1.00]<br>p<0.001	0.06 [-0.27,0.40]<br>d=0.11 [-0.31, 0.53]<br>p=1.0	-0.31 [-0.60,-0.02]<br>d=-0.55 [-0.91, -0.18]<br>p=1.0	-0.73 [-1.04,-0.43]<br>d=-1.05 [-1.45, -0.64]<br>p<0.001	-0.37 [-0.71,-0.03]<br>d=-0.65 [-1.09, -0.21]<br>p=1.0	-0.80 [-1.15,-0.44]<br>d=-1.07 [-1.54, -0.60]<br>p<0.001	-0.43 [-0.73,-0.12]<br>d=-0.61 [-1.00, -0.21]<br>p=0.175
Banking	AVG Correct PIN Duration msec	✓	F=7.249<br>η^2=0.11 [0.04, 1.00]<br>p<0.001	0.19 [-0.17,0.55]<br>d=0.28 [-0.14, 0.71]<br>p=1.0	-0.14 [-0.46,0.17]<br>d=-0.20 [-0.57, 0.18]<br>p=1.0	-0.44 [-0.76,-0.12]<br>d=-0.69 [-1.08, -0.30]<br>p=0.148	-0.34 [-0.70,0.03]<br>d=-0.42 [-0.85, 0.02]<br>p=1.0	-0.63 [-1.00,-0.25]<br>d=-0.92 [-1.38, -0.45]<br>p=0.008	-0.29 [-0.62,0.04]<br>d=-0.39 [-0.78, 0.01]<br>p=1.0
Banking	AVG Duration Attempts BankApp msec	✓	F=17.029<br>η^2=0.22 [0.13, 1.00]<br>p<0.001	0.09 [-0.18,0.36]<br>d=0.18 [-0.25, 0.60]<br>p=1.0	-0.26 [-0.49,-0.03]<br>d=-0.48 [-0.85, -0.12]<br>p=1.0	-0.56 [-0.80,-0.32]<br>d=-1.10 [-1.50, -0.69]<br>p<0.001	-0.35 [-0.63,-0.07]<br>d=-0.59 [-1.03, -0.15]<br>p=0.415	-0.65 [-0.94,-0.37]<br>d=-1.17 [-1.64, -0.69]<br>p<0.001	-0.30 [-0.55,-0.06]<br>d=-0.51 [-0.90, -0.13]<br>p=0.527
Banking	AVG Duration Correct Attempts BankApp msec	✓	F=15.411<br>η^2=0.21 [0.12, 1.00]<br>p<0.001	0.08 [-0.19,0.36]<br>d=0.16 [-0.26, 0.59]<br>p=1.0	-0.26 [-0.50,-0.02]<br>d=-0.47 [-0.85, -0.09]<br>p=1.0	-0.56 [-0.81,-0.31]<br>d=-1.08 [-1.49, -0.67]<br>p<0.001	-0.34 [-0.62,-0.06]<br>d=-0.57 [-1.01, -0.12]<br>p=0.688	-0.64 [-0.93,-0.35]<br>d=-1.14 [-1.62, -0.65]<br>p<0.001	-0.30 [-0.55,-0.04]<br>d=-0.50 [-0.90, -0.09]<br>p=0.909
Banking	AVG Duration Correct Confirm msec	✓	F=4.261<br>η^2=0.07 [0.01, 1.00]<br>p=0.006	-0.04 [-0.38,0.29]<br>d=-0.09 [-0.51, 0.34]<br>p=1.0	-0.32 [-0.61,-0.02]<br>d=-0.53 [-0.90, -0.15]<br>p=1.0	-0.34 [-0.64,-0.03]<br>d=-0.51 [-0.90, -0.12]<br>p=0.872	-0.27 [-0.62,0.08]<br>d=-0.41 [-0.85, 0.02]<br>p=1.0	-0.29 [-0.65,0.06]<br>d=-0.40 [-0.85, 0.04]<br>p=1.0	-0.02 [-0.34,0.29]<br>d=-0.03 [-0.42, 0.37]<br>p=1.0
Banking	AVG PIN Duration msec	✓	F=7.684<br>η^2=0.11 [0.04, 1.00]<br>p<0.001	0.15 [-0.21,0.51]<br>d=0.22 [-0.21, 0.64]<br>p=1.0	-0.14 [-0.45,0.17]<br>d=-0.19 [-0.55, 0.17]<br>p=1.0	-0.48 [-0.80,-0.16]<br>d=-0.74 [-1.12, -0.35]<br>p=0.043	-0.29 [-0.65,0.08]<br>d=-0.36 [-0.79, 0.07]<br>p=1.0	-0.63 [-1.00,-0.25]<br>d=-0.92 [-1.38, -0.46]<br>p=0.009	-0.34 [-0.66,-0.01]<br>d=-0.45 [-0.84, -0.07]<br>p=1.0
Banking	Correct Amount Attempts count	✓	F=0.621<br>η^2=0.01 [0.00, 1.00]<br>p=0.602	–	–	–	–	–	–
Banking	Correct Attempts BankApp count	✓	F=1.383<br>η^2=0.02 [0.00, 1.00]<br>p=0.25	–	–	–	–	–	–
Banking	Correct PIN Attempts count	✓	F=0.979<br>η^2=0.02 [0.00, 1.00]<br>p=0.404	–	–	–	–	–	–
Banking	SUM Amount Duration msec	✓	F=14.233<br>η^2=0.19 [0.10, 1.00]<br>p<0.001	0.06 [-0.30,0.43]<br>d=0.10 [-0.32, 0.52]<br>p=1.0	-0.32 [-0.63,-0.01]<br>d=-0.48 [-0.85, -0.12]<br>p=1.0	-0.72 [-1.05,-0.40]<br>d=-0.95 [-1.34, -0.56]<br>p<0.001	-0.38 [-0.76,-0.01]<br>d=-0.60 [-1.03, -0.17]<br>p=1.0	-0.79 [-1.17,-0.41]<br>d=-1.01 [-1.47, -0.55]<br>p<0.001	-0.40 [-0.74,-0.07]<br>d=-0.53 [-0.92, -0.15]<br>p=0.666
Banking	SUM Correct Amount Duration msec	✓	F=15.532<br>η^2=0.20 [0.12, 1.00]<br>p<0.001	0.04 [-0.30,0.39]<br>d=0.07 [-0.35, 0.49]<br>p=1.0	-0.31 [-0.61,-0.01]<br>d=-0.54 [-0.90, -0.17]<br>p=1.0	-0.73 [-1.03,-0.42]<br>d=-1.02 [-1.42, -0.62]<br>p<0.001	-0.35 [-0.70,-0.01]<br>d=-0.59 [-1.03, -0.16]<br>p=1.0	-0.77 [-1.13,-0.41]<br>d=-1.00 [-1.47, -0.53]<br>p<0.001	-0.41 [-0.73,-0.10]<br>d=-0.58 [-0.98, -0.19]<br>p=0.298
Banking	SUM Correct PIN Duration msec	✓	F=6.933<br>η^2=0.11 [0.04, 1.00]<br>p<0.001	0.21 [-0.16,0.57]<br>d=0.29 [-0.14, 0.72]<br>p=1.0	-0.15 [-0.47,0.17]<br>d=-0.20 [-0.57, 0.17]<br>p=1.0	-0.43 [-0.75,-0.10]<br>d=-0.66 [-1.05, -0.27]<br>p=0.232	-0.35 [-0.73,0.02]<br>d=-0.44 [-0.88, 0.00]<br>p=1.0	-0.63 [-1.01,-0.25]<br>d=-0.91 [-1.37, -0.44]<br>p=0.011	-0.28 [-0.61,0.06]<br>d=-0.37 [-0.77, 0.02]<br>p=1.0

Continued on next page

RMT	Feature	Log	Ancova	HC vs. PreAD	HC vs. ProAD	HC vs. MildAD	PreAD vs. ProAD	PreAD vs. MildAD	ProAD vs. MildAD
Banking	SUM Duration Attempts BankApp msec	✓	F=14.291<br>η^2=0.19 [0.10, 1.00]<br>p<0.001	0.08 [-0.21,0.38]<br>d=0.15 [-0.27, 0.58]<br>p=1.0	-0.25 [-0.49,0.00]<br>d=-0.43 [-0.79, -0.06]<br>p=1.0	-0.56 [-0.81,-0.30]<br>d=-0.98 [-1.37, -0.58]<br>p<0.001	-0.33 [-0.63,-0.04]<br>d=-0.55 [-0.98, -0.11]<br>p=1.0	-0.64 [-0.94,-0.34]<br>d=-1.07 [-1.54, -0.60]<br>p<0.001	-0.31 [-0.57,-0.05]<br>d=-0.51 [-0.89, -0.12]<br>p=0.79
Banking	SUM Duration Correct Attempts BankApp msec	✓	F=12.775<br>η^2=0.18 [0.10, 1.00]<br>p<0.001	0.07 [-0.22,0.36]<br>d=0.13 [-0.29, 0.56]<br>p=1.0	-0.25 [-0.51,0.00]<br>d=-0.44 [-0.81, -0.06]<br>p=1.0	-0.54 [-0.81,-0.28]<br>d=-0.97 [-1.37, -0.56]<br>p<0.001	-0.32 [-0.62,-0.03]<br>d=-0.53 [-0.97, -0.08]<br>p=1.0	-0.61 [-0.92,-0.31]<br>d=-1.02 [-1.49, -0.54]<br>p<0.001	-0.29 [-0.56,-0.02]<br>d=-0.47 [-0.87, -0.06]<br>p=1.0
Banking	SUM Duration Correct Confirm msec	✓	F=5.216<br>η^2=0.08 [0.02, 1.00]<br>p=0.002	-0.09 [-0.43,0.25]<br>d=-0.18 [-0.60, 0.24]<br>p=1.0	-0.37 [-0.67,-0.07]<br>d=-0.61 [-0.99, -0.23]<br>p=0.549	-0.38 [-0.69,-0.07]<br>d=-0.58 [-0.97, -0.19]<br>p=0.384	-0.28 [-0.63,0.07]<br>d=-0.41 [-0.84, 0.03]<br>p=1.0	-0.29 [-0.64,0.07]<br>d=-0.39 [-0.83, 0.06]<br>p=1.0	-0.01 [-0.33,0.31]<br>d=-0.01 [-0.41, 0.38]<br>p=1.0
Banking	SUM PIN Duration msec	✓	F=7.045<br>η^2=0.10 [0.04, 1.00]<br>p<0.001	0.14 [-0.23,0.51]<br>d=0.20 [-0.22, 0.62]<br>p=1.0	-0.14 [-0.45,0.18]<br>d=-0.18 [-0.54, 0.18]<br>p=1.0	-0.47 [-0.80,-0.14]<br>d=-0.70 [-1.08, -0.32]<br>p=0.074	-0.28 [-0.65,0.10]<br>d=-0.35 [-0.78, 0.08]<br>p=1.0	-0.61 [-1.00,-0.23]<br>d=-0.89 [-1.34, -0.42]<br>p=0.018	-0.33 [-0.67,0.00]<br>d=-0.45 [-0.83, -0.06]<br>p=1.0
Banking	Total Attempts Amount count	✓	F=0.023<br>η^2=0.00 [0.00, 1.00]<br>p=0.995	–	–	–	–	–	–
Banking	Total Attempts BankApp count	✓	F=0.024<br>η^2=0.00 [0.00, 1.00]<br>p=0.995	–	–	–	–	–	–
Banking	Total Attempts PIN count	✓	F=0.023<br>η^2=0.00 [0.00, 1.00]<br>p=0.995	–	–	–	–	–	–
Banking	Total Cancel attempts count	✓	F=0.028<br>η^2=0.00 [0.00, 1.00]<br>p=0.994	–	–	–	–	–	–
Banking	Total Confirm attempts count	✓	F=0.112<br>η^2=0.00 [0.00, 1.00]<br>p=0.953	–	–	–	–	–	–
Banking	Total Correct Confirm attempts count	✓	F=1.482<br>η^2=0.02 [0.00, 1.00]<br>p=0.221	–	–	–	–	–	–
Banking	Total Wrong Confirm attempts count	✓	F=1.17<br>η^2=0.02 [0.00, 1.00]<br>p=0.322	–	–	–	–	–	–
Banking	Wrong Amount Attempts count	✓	F=0.568<br>η^2=0.01 [0.00, 1.00]<br>p=0.637	–	–	–	–	–	–
Banking	Wrong Attempts BankApp count	✓	F=1.17<br>η^2=0.02 [0.00, 1.00]<br>p=0.322	–	–	–	–	–	–
Banking	Wrong PIN Attempts count	✓	F=0.319<br>η^2=0.01 [0.00, 1.00]<br>p=0.812	–	–	–	–	–	–
Fitbit	NREM pct	✓	F=6.199<br>η^2=0.10 [0.03, 1.00]<br>p<0.001	0.00 [-0.03,0.03]<br>d=0.00 [-0.41, 0.40]<br>p=1.0	-0.03 [-0.06,-0.01]<br>d=-0.60 [-0.97, -0.23]<br>p=0.227	-0.03 [-0.05,0.00]<br>d=-0.59 [-0.98, -0.20]<br>p=0.845	-0.03 [-0.06,0.00]<br>d=-0.57 [-1.00, -0.15]<br>p=0.914	-0.03 [-0.06,0.00]<br>d=-0.58 [-1.02, -0.14]<br>p=1.0	0.00 [-0.02,0.03]<br>d=0.09 [-0.30, 0.48]<br>p=1.0
Fitbit	REM pct	✓	F=5.784<br>η^2=0.09 [0.03, 1.00]<br>p<0.001	0.08 [-0.20,0.36]<br>d=0.32 [-0.09, 0.73]<br>p=1.0	0.39 [0.13,0.64]<br>d=0.59 [0.22, 0.96]<br>p=0.043	0.22 [-0.04,0.49]<br>d=0.76 [0.37, 1.15]<br>p=1.0	0.31 [0.02,0.60]<br>d=0.42 [-0.01, 0.84]<br>p=1.0	0.15 [-0.15,0.44]<br>d=0.45 [0.01, 0.89]<br>p=1.0	-0.16 [-0.44,0.11]<br>d=-0.22 [-0.62, 0.17]<br>p=1.0
Fitbit	awake pct	×	F=3.504<br>η^2=0.06 [0.01, 1.00]<br>p=0.017	-1.13 [-2.23,-0.03]<br>d=-0.57 [-0.98, -0.15]<br>p=1.0	-0.98 [-1.97,0.01]<br>d=-0.51 [-0.88, -0.14]<br>p=1.0	-0.92 [-1.94,0.10]<br>d=-0.51 [-0.90, -0.13]<br>p=1.0	0.15 [-0.99,1.29]<br>d=0.04 [-0.38, 0.45]<br>p=1.0	0.21 [-0.96,1.37]<br>d=0.10 [-0.33, 0.53]<br>p=1.0	0.06 [-1.01,1.13]<br>d=0.06 [-0.33, 0.45]<br>p=1.0
Fitbit	awakenings	×	F=3.666<br>η^2=0.06 [0.01, 1.00]<br>p=0.013	-0.40 [-0.83,0.03]<br>d=-0.54 [-0.95, -0.12]<br>p=1.0	-0.08 [-0.47,0.31]<br>d=-0.15 [-0.52, 0.21]<br>p=1.0	-0.42 [-0.82,-0.02]<br>d=-0.54 [-0.92, -0.15]<br>p=1.0	0.32 [-0.13,0.76]<br>d=0.31 [-0.11, 0.73]<br>p=1.0	-0.02 [-0.48,0.43]<br>d=-0.03 [-0.46, 0.40]<br>p=1.0	-0.34 [-0.76,0.07]<br>d=-0.33 [-0.72, 0.07]<br>p=1.0
Fitbit	dailyMaxHeartRate	✓	F=3.451<br>η^2=0.05 [0.01, 1.00]<br>p=0.018	0.00 [-0.04,0.04]<br>d=0.00 [-0.40, 0.40]<br>p=1.0	0.04 [0.00,0.08]<br>d=0.44 [0.08, 0.80]<br>p=1.0	0.03 [-0.01,0.07]<br>d=0.37 [0.00, 0.74]<br>p=1.0	0.04 [0.00,0.09]<br>d=0.41 [-0.01, 0.83]<br>p=1.0	0.03 [-0.02,0.08]<br>d=0.35 [-0.08, 0.78]<br>p=1.0	-0.01 [-0.05,0.03]<br>d=-0.09 [-0.47, 0.29]<br>p=1.0

Continued on next page

RMT	Feature	Log	Ancova	HC vs. PreAD	HC vs. ProAD	HC vs. MildAD	PreAD vs. ProAD	PreAD vs. MildAD	ProAD vs. MildAD
Fitbit	dailyMeanAsleepHours	✓	F=2.403<br>η^2=0.04 [0.00, 1.00]<br>p=0.069	–	–	–	–	–	–
Fitbit	dailyMeanAwakeHours	×	F=1.091<br>η^2=0.02 [0.00, 1.00]<br>p=0.354	–	–	–	–	–	–
Fitbit	dailyMeanBedtimeHours	✓	F=2.22<br>η^2=0.03 [0.00, 1.00]<br>p=0.087	–	–	–	–	–	–
Fitbit	dailyMeanHeartRate	×	F=1.725<br>η^2=0.03 [0.00, 1.00]<br>p=0.163	–	–	–	–	–	–
Fitbit	dailyMeanRemHours	×	F=9.467<br>η^2=0.13 [0.06, 1.00]<br>p<0.001	0.12 [-0.08,0.31]<br>d=0.31 [-0.10, 0.71]<br>p=1.0	0.31 [0.13,0.48]<br>d=0.81 [0.44, 1.18]<br>p=0.004	0.29 [0.12,0.47]<br>d=0.76 [0.38, 1.13]<br>p=0.01	0.19 [-0.01,0.39]<br>d=0.46 [0.03, 0.88]<br>p=1.0	0.18 [-0.03,0.38]<br>d=0.41 [-0.02, 0.84]<br>p=1.0	-0.01 [-0.20,0.17]<br>d=-0.03 [-0.41, 0.35]<br>p=1.0
Fitbit	dailyMeanSteps	✓	F=6.23<br>η^2=0.09 [0.03, 1.00]<br>p<0.001	-0.06 [-0.49,0.37]<br>d=-0.06 [-0.46, 0.33]<br>p=1.0	0.24 [-0.14,0.62]<br>d=0.22 [-0.13, 0.57]<br>p=1.0	0.57 [0.18,0.96]<br>d=0.45 [0.08, 0.81]<br>p=0.06	0.30 [-0.14,0.74]<br>d=0.33 [-0.08, 0.73]<br>p=1.0	0.63 [0.18,1.08]<br>d=0.51 [0.09, 0.93]<br>p=0.097	0.33 [-0.08,0.73]<br>d=0.28 [-0.09, 0.65]<br>p=1.0
Fitbit	dailyMinHeartRate	×	F=0.802<br>η^2=0.01 [0.00, 1.00]<br>p=0.494	–	–	–	–	–	–
Fitbit	deep pct	✓	F=4.265<br>η^2=0.07 [0.01, 1.00]<br>p=0.006	0.10 [-0.19,0.39]<br>d=0.28 [-0.13, 0.69]<br>p=1.0	0.34 [0.08,0.61]<br>d=0.54 [0.17, 0.91]<br>p=0.345	0.24 [-0.03,0.51]<br>d=0.68 [0.29, 1.07]<br>p=1.0	0.24 [-0.06,0.55]<br>d=0.32 [-0.10, 0.74]<br>p=1.0	0.14 [-0.17,0.45]<br>d=0.31 [-0.12, 0.74]<br>p=1.0	-0.10 [-0.38,0.18]<br>d=-0.13 [-0.53, 0.26]<br>p=1.0
Fitbit	hypersomnia	✓	F=1.921<br>η^2=0.03 [0.00, 1.00]<br>p=0.128	–	–	–	–	–	–
Fitbit	insomnia	✓	F=1.452<br>η^2=0.02 [0.00, 1.00]<br>p=0.229	–	–	–	–	–	–
Fitbit	light pct	✓	F=11.909<br>η^2=0.17 [0.08, 1.00]<br>p<0.001	-0.01 [-0.05,0.03]<br>d=-0.14 [-0.54, 0.27]<br>p=1.0	-0.08 [-0.12,-0.04]<br>d=-0.87 [-1.25, -0.49]<br>p<0.001	-0.07 [-0.11,-0.03]<br>d=-0.86 [-1.26, -0.47]<br>p=0.008	-0.07 [-0.11,-0.02]<br>d=-0.67 [-1.10, -0.24]<br>p=0.114	-0.06 [-0.10,-0.01]<br>d=-0.65 [-1.09, -0.20]<br>p=0.345	0.01 [-0.03,0.05]<br>d=0.08 [-0.31, 0.47]<br>p=1.0
Fitbit	rem latency	✓	F=3.284<br>η^2=0.05 [0.00, 1.00]<br>p=0.022	-0.08 [-0.22,0.05]<br>d=-0.32 [-0.73, 0.09]<br>p=1.0	-0.15 [-0.27,-0.02]<br>d=-0.58 [-0.95, -0.21]<br>p=0.878	-0.10 [-0.23,0.03]<br>d=-0.36 [-0.74, 0.02]<br>p=1.0	-0.06 [-0.21,0.08]<br>d=-0.24 [-0.66, 0.18]<br>p=1.0	-0.02 [-0.16,0.13]<br>d=-0.06 [-0.49, 0.37]<br>p=1.0	0.05 [-0.09,0.18]<br>d=0.17 [-0.22, 0.56]<br>p=1.0
Fitbit	sleep efficiency	×	F=3.504<br>η^2=0.06 [0.01, 1.00]<br>p=0.017	1.13 [0.03,2.23]<br>d=0.57 [0.15, 0.98]<br>p=1.0	0.98 [-0.01,1.97]<br>d=0.51 [0.14, 0.88]<br>p=1.0	0.92 [-0.10,1.94]<br>d=0.51 [0.13, 0.90]<br>p=1.0	-0.15 [-1.29,0.99]<br>d=-0.04 [-0.45, 0.38]<br>p=1.0	-0.21 [-1.37,0.96]<br>d=-0.10 [-0.53, 0.33]<br>p=1.0	-0.06 [-1.13,1.01]<br>d=-0.06 [-0.45, 0.33]<br>p=1.0
Fitbit	sleep offset	✓	F=0.166<br>η^2=0.00 [0.00, 1.00]<br>p=0.919	–	–	–	–	–	–
Fitbit	sleep onset	✓	F=0.775<br>η^2=0.01 [0.00, 1.00]<br>p=0.51	–	–	–	–	–	–
Fitbit	time in bed	×	F=0.483<br>η^2=0.01 [0.00, 1.00]<br>p=0.694	–	–	–	–	–	–
Fitbit	total sleep time	×	F=0.66<br>η^2=0.01 [0.00, 1.00]<br>p=0.578	–	–	–	–	–	–
Fitbit	wearTimeMinutes	✓	F=1.277<br>η^2=0.02 [0.00, 1.00]<br>p=0.283	–	–	–	–	–	–
Fitbit	wearTimePercentage	✓	F=1.277<br>η^2=0.02 [0.00, 1.00]<br>p=0.283	–	–	–	–	–	–

Continued on next page

RMT	Feature	Log	Ancova	HC vs. PreAD	HC vs. ProAD	HC vs. MildAD	PreAD vs. ProAD	PreAD vs. MildAD	ProAD vs. MildAD
Mezurio	audio articulation rate	✓	F=2.836<br>η^2=0.06 [0.00, 1.00]<br>p=0.04	0.00 [-0.15,0.15]<br>d=0.02 [-0.43, 0.47]<br>p=1.0	0.12 [-0.01,0.25]<br>d=0.44 [0.06, 0.83]<br>p=1.0	0.11 [-0.04,0.27]<br>d=0.61 [0.15, 1.07]<br>p=1.0	0.12 [-0.04,0.28]<br>d=0.36 [-0.12, 0.84]<br>p=1.0	0.11 [-0.07,0.30]<br>d=0.50 [-0.05, 1.05]<br>p=1.0	-0.01 [-0.17,0.16]<br>d=-0.02 [-0.50, 0.47]<br>p=1.0
Mezurio	audio average pause duration	✓	F=4.589<br>η^2=0.09 [0.02, 1.00]<br>p=0.004	0.04 [-0.23,0.31]<br>d=0.10 [-0.35, 0.55]<br>p=1.0	-0.23 [-0.47,0.00]<br>d=-0.50 [-0.89, -0.12]<br>p=1.0	-0.29 [-0.56,-0.01]<br>d=-0.63 [-1.09, -0.16]<br>p=1.0	-0.28 [-0.57,0.02]<br>d=-0.62 [-1.10, -0.12]<br>p=1.0	-0.33 [-0.66,0.00]<br>d=-0.76 [-1.32, -0.20]<br>p=1.0	-0.05 [-0.35,0.24]<br>d=-0.11 [-0.59, 0.38]<br>p=1.0
Mezurio	audio average syllable duration	✓	F=2.459<br>η^2=0.05 [0.00, 1.00]<br>p=0.065	–	–	–	–	–	–
Mezurio	audio file duration in second	✓	F=1.097<br>η^2=0.02 [0.00, 1.00]<br>p=0.352	–	–	–	–	–	–
Mezurio	audio hesitation ratio	×	F=3.838<br>η^2=0.07 [0.01, 1.00]<br>p=0.011	0.02 [-0.07,0.10]<br>d=0.13 [-0.32, 0.58]<br>p=1.0	-0.05 [-0.12,0.02]<br>d=-0.37 [-0.75, 0.02]<br>p=1.0	-0.09 [-0.17,0.00]<br>d=-0.60 [-1.06, -0.14]<br>p=1.0	-0.07 [-0.16,0.02]<br>d=-0.54 [-1.03, -0.05]<br>p=1.0	-0.11 [-0.21,-0.01]<br>d=-0.82 [-1.38, -0.25]<br>p=1.0	-0.04 [-0.13,0.06]<br>d=-0.24 [-0.72, 0.25]<br>p=1.0
Mezurio	audio number of pauses	✓	F=0.568<br>η^2=0.01 [0.00, 1.00]<br>p=0.637	–	–	–	–	–	–
Mezurio	audio number of syllables	✓	F=0.992<br>η^2=0.02 [0.00, 1.00]<br>p=0.398	–	–	–	–	–	–
Mezurio	audio rms energy	✓	F=0.339<br>η^2=0.01 [0.00, 1.00]<br>p=0.797	–	–	–	–	–	–
Mezurio	audio speaking rate	×	F=7.863<br>η^2=0.14 [0.05, 1.00]<br>p<0.001	-0.08 [-0.45,0.30]<br>d=-0.13 [-0.58, 0.32]<br>p=1.0	0.43 [0.12,0.75]<br>d=0.69 [0.29, 1.08]<br>p=0.2	0.49 [0.12,0.87]<br>d=0.74 [0.27, 1.21]<br>p=0.22	0.51 [0.11,0.91]<br>d=0.86 [0.36, 1.36]<br>p=0.41	0.57 [0.12,1.02]<br>d=0.88 [0.31, 1.44]<br>p=0.265	0.06 [-0.34,0.47]<br>d=0.09 [-0.40, 0.57]<br>p=1.0
Mezurio	audio total speech duration	✓	F=0.647<br>η^2=0.01 [0.00, 1.00]<br>p=0.586	–	–	–	–	–	–
Mezurio	ost F0semitoneFrom27 5Hz sma3nz amean	✓	F=1.913<br>η^2=0.04 [0.00, 1.00]<br>p=0.13	–	–	–	–	–	–
Mezurio	ost F0semitoneFrom27 5Hz sma3nz meanFallingSlope	✓	F=1.69<br>η^2=0.03 [0.00, 1.00]<br>p=0.172	–	–	–	–	–	–
Mezurio	ost F0semitoneFrom27 5Hz sma3nz meanRisingSlope	✓	F=1.425<br>η^2=0.03 [0.00, 1.00]<br>p=0.238	–	–	–	–	–	–
Mezurio	ost F0semitoneFrom27 5Hz sma3nz pctlrange0 2	✓	F=1.825<br>η^2=0.04 [0.00, 1.00]<br>p=0.145	–	–	–	–	–	–
Mezurio	ost F0semitoneFrom27 5Hz sma3nz percentile20 0	✓	F=1.915<br>η^2=0.04 [0.00, 1.00]<br>p=0.13	–	–	–	–	–	–
Mezurio	ost F0semitoneFrom27 5Hz sma3nz percentile50 0	✓	F=1.919<br>η^2=0.04 [0.00, 1.00]<br>p=0.129	–	–	–	–	–	–
Mezurio	ost F0semitoneFrom27 5Hz sma3nz percentile80 0	✓	F=1.907<br>η^2=0.04 [0.00, 1.00]<br>p=0.131	–	–	–	–	–	–
Mezurio	ost F0semitoneFrom27 5Hz sma3nz stddevFallingSlope	✓	F=2.086<br>η^2=0.04 [0.00, 1.00]<br>p=0.105	–	–	–	–	–	–
Mezurio	ost F0semitoneFrom27 5Hz sma3nz stddevNorm	✓	F=1.567<br>η^2=0.03 [0.00, 1.00]<br>p=0.2	–	–	–	–	–	–

Continued on next page

RMT	Feature	Log	Ancova	HC vs. PreAD	HC vs. ProAD	HC vs. MildAD	PreAD vs. ProAD	PreAD vs. MildAD	ProAD vs. MildAD
Mezurio	ost F0semitoneFrom27 5Hz sma3nz stddevRisingS-lope	✓	F=1.274<br>η^2=0.03 [0.00, 1.00]<br>p=0.286	–	–	–	–	–	–
Mezurio	ost F1amplitudeLogRelF0 sma3nz amean	×	F=6.89<br>η^2=0.12 [0.04, 1.00]<br>p<0.001	-8.29 [-19.76,3.18]<br>d=-0.44 [-0.89, 0.01]<br>p=1.0	8.37 [-1.40,18.14]<br>d=0.40 [0.01, 0.78]<br>p=1.0	12.63 [1.01,24.26]<br>d=0.61 [0.15, 1.07]<br>p=0.932	16.65 [4.38,28.93]<br>d=0.82 [0.32, 1.31]<br>p=0.216	20.92 [7.13,34.72]<br>d=1.09 [0.51, 1.66]<br>p=0.04	4.27 [-8.15,16.69]<br>d=0.19 [-0.30, 0.67]<br>p=1.0
Mezurio	ost F1amplitudeLogRelF0 sma3nz stddevNorm	×	F=6.297<br>η^2=0.12 [0.04, 1.00]<br>p<0.001	0.07 [-0.03,0.16]<br>d=0.43 [-0.02, 0.88]<br>p=1.0	-0.07 [-0.15,0.01]<br>d=-0.37 [-0.76, 0.01]<br>p=1.0	-0.10 [-0.20,-0.01]<br>d=-0.62 [-1.08, -0.16]<br>p=1.0	-0.13 [-0.23,-0.03]<br>d=-0.74 [-1.23, -0.24]<br>p=0.384	-0.17 [-0.28,-0.05]<br>d=-1.10 [-1.67, -0.51]<br>p=0.065	-0.04 [-0.14,0.07]<br>d=-0.18 [-0.67, 0.30]<br>p=1.0
Mezurio	ost F1bandwidth sma3nz amean	✓	F=1.798<br>η^2=0.04 [0.00, 1.00]<br>p=0.15	–	–	–	–	–	–
Mezurio	ost F1bandwidth sma3nz stddevNorm	✓	F=1.88<br>η^2=0.04 [0.00, 1.00]<br>p=0.136	–	–	–	–	–	–
Mezurio	ost F1frequency sma3nz amean	✓	F=1.857<br>η^2=0.04 [0.00, 1.00]<br>p=0.139	–	–	–	–	–	–
Mezurio	ost F1frequency sma3nz stddevNorm	✓	F=1.679<br>η^2=0.03 [0.00, 1.00]<br>p=0.174	–	–	–	–	–	–
Mezurio	ost F2amplitudeLogRelF0 sma3nz amean	×	F=6.898<br>η^2=0.12 [0.04, 1.00]<br>p<0.001	-8.72 [-19.78,2.33]<br>d=-0.48 [-0.93, -0.03]<br>p=1.0	8.02 [-1.40,17.44]<br>d=0.39 [0.00, 0.77]<br>p=1.0	11.32 [0.11,22.52]<br>d=0.56 [0.10, 1.02]<br>p=1.0	16.75 [4.92,28.58]<br>d=0.85 [0.34, 1.34]<br>p=0.134	20.04 [6.74,33.34]<br>d=1.07 [0.49, 1.65]<br>p=0.043	3.29 [-8.68,15.26]<br>d=0.15 [-0.34, 0.63]<br>p=1.0
Mezurio	ost F2amplitudeLogRelF0 sma3nz stddevNorm	×	F=6.361<br>η^2=0.12 [0.04, 1.00]<br>p<0.001	0.06 [-0.02,0.15]<br>d=0.48 [0.03, 0.93]<br>p=1.0	-0.06 [-0.13,0.01]<br>d=-0.38 [-0.76, 0.01]<br>p=1.0	-0.08 [-0.17,0.00]<br>d=-0.57 [-1.03, -0.11]<br>p=1.0	-0.12 [-0.21,-0.03]<br>d=-0.78 [-1.27, -0.28]<br>p=0.206	-0.15 [-0.25,-0.04]<br>d=-1.08 [-1.65, -0.50]<br>p=0.072	-0.02 [-0.11,0.07]<br>d=-0.13 [-0.62, 0.36]<br>p=1.0
Mezurio	ost F2bandwidth sma3nz amean	✓	F=1.787<br>η^2=0.04 [0.00, 1.00]<br>p=0.152	–	–	–	–	–	–
Mezurio	ost F2bandwidth sma3nz stddevNorm	✓	F=1.932<br>η^2=0.04 [0.00, 1.00]<br>p=0.127	–	–	–	–	–	–
Mezurio	ost F2frequency sma3nz amean	✓	F=1.831<br>η^2=0.04 [0.00, 1.00]<br>p=0.144	–	–	–	–	–	–
Mezurio	ost F2frequency sma3nz stddevNorm	✓	F=1.715<br>η^2=0.03 [0.00, 1.00]<br>p=0.167	–	–	–	–	–	–
Mezurio	ost F3amplitudeLogRelF0 sma3nz amean	×	F=7.022<br>η^2=0.13 [0.04, 1.00]<br>p<0.001	-8.16 [-18.84,2.52]<br>d=-0.46 [-0.92, -0.01]<br>p=1.0	8.17 [-0.93,17.27]<br>d=0.41 [0.02, 0.79]<br>p=1.0	11.10 [0.27,21.93]<br>d=0.57 [0.10, 1.02]<br>p=1.0	16.33 [4.90,27.76]<br>d=0.86 [0.36, 1.36]<br>p=0.12	19.26 [6.42,32.11]<br>d=1.06 [0.48, 1.63]<br>p=0.045	2.93 [-8.64,14.50]<br>d=0.14 [-0.35, 0.62]<br>p=1.0
Mezurio	ost F3amplitudeLogRelF0 sma3nz stddevNorm	×	F=6.598<br>η^2=0.12 [0.04, 1.00]<br>p<0.001	0.05 [-0.02,0.13]<br>d=0.45 [-0.01, 0.90]<br>p=1.0	-0.06 [-0.13,0.00]<br>d=-0.42 [-0.81, -0.04]<br>p=1.0	-0.08 [-0.16,0.00]<br>d=-0.58 [-1.04, -0.11]<br>p=1.0	-0.12 [-0.20,-0.03]<br>d=-0.80 [-1.29, -0.30]<br>p=0.164	-0.13 [-0.23,-0.04]<br>d=-1.05 [-1.62, -0.47]<br>p=0.084	-0.02 [-0.10,0.07]<br>d=-0.10 [-0.59, 0.38]<br>p=1.0
Mezurio	ost F3bandwidth sma3nz amean	✓	F=1.78<br>η^2=0.04 [0.00, 1.00]<br>p=0.154	–	–	–	–	–	–
Mezurio	ost F3bandwidth sma3nz stddevNorm	✓	F=1.849<br>η^2=0.04 [0.00, 1.00]<br>p=0.141	–	–	–	–	–	–
Mezurio	ost F3frequency sma3nz amean	✓	F=1.811<br>η^2=0.04 [0.00, 1.00]<br>p=0.148	–	–	–	–	–	–
Mezurio	ost F3frequency sma3nz stddevNorm	✓	F=1.654<br>η^2=0.03 [0.00, 1.00]<br>p=0.18	–	–	–	–	–	–

Continued on next page

RMT	Feature	Log	Ancova	HC vs. PreAD	HC vs. ProAD	HC vs. MildAD	PreAD vs. ProAD	PreAD vs. MildAD	ProAD vs. MildAD
Mezurio	ost HNRdBACF sma3nz amean	×	F=2.416 η^2=0.05 [0.00, 1.00] p=0.069	–	–	–	–	–	–
Mezurio	ost HNRdBACF sma3nz stddevNorm	✓	F=1.618 η^2=0.03 [0.00, 1.00] p=0.188	–	–	–	–	–	–
Mezurio	ost MeanUnvoicedSegmentLength	✓	F=3.087 η^2=0.06 [0.00, 1.00] p=0.029	0.09 [-0.40,0.58] d=0.13 [-0.31, 0.58] p=1.0	-0.32 [-0.73,0.10] d=-0.39 [-0.78, -0.01] p=1.0	-0.43 [-0.92,0.07] d=-0.58 [-1.04, -0.11] p=1.0	-0.41 [-0.93,0.12] d=-0.43 [-0.92, 0.05] p=1.0	-0.52 [-1.10,0.07] d=-0.59 [-1.13, -0.03] p=1.0	-0.11 [-0.64,0.42] d=-0.11 [-0.60, 0.37] p=1.0
Mezurio	ost MeanVoicedSegmentLengthSec	✓	F=1.895 η^2=0.04 [0.00, 1.00] p=0.133	–	–	–	–	–	–
Mezurio	ost StddevUnvoicedSegmentLength	✓	F=1.164 η^2=0.02 [0.00, 1.00] p=0.326	–	–	–	–	–	–
Mezurio	ost StddevVoicedSegmentLengthSec	✓	F=1.682 η^2=0.03 [0.00, 1.00] p=0.174	–	–	–	–	–	–
Mezurio	ost VoicedSegmentsPerSec	✓	F=2.45 η^2=0.05 [0.00, 1.00] p=0.066	–	–	–	–	–	–
Mezurio	ost hammarbergIndexUV sma3nz amean	✓	F=0.765 η^2=0.02 [0.00, 1.00] p=0.515	–	–	–	–	–	–
Mezurio	ost hammarbergIndexV sma3nz amean	✓	F=1.618 η^2=0.03 [0.00, 1.00] p=0.188	–	–	–	–	–	–
Mezurio	ost hammarbergIndexV sma3nz stddevNorm	✓	F=2.138 η^2=0.04 [0.00, 1.00] p=0.098	–	–	–	–	–	–
Mezurio	ost jitterLocal sma3nz amean	✓	F=1.443 η^2=0.03 [0.00, 1.00] p=0.233	–	–	–	–	–	–
Mezurio	ost jitterLocal sma3nz stddevNorm	✓	F=1.776 η^2=0.04 [0.00, 1.00] p=0.154	–	–	–	–	–	–
Mezurio	ost logRelF0 H1 A3 sma3nz amean	✓	F=1.597 η^2=0.03 [0.00, 1.00] p=0.193	–	–	–	–	–	–
Mezurio	ost logRelF0 H1 A3 sma3nz stddevNorm	✓	F=2.001 η^2=0.04 [0.00, 1.00] p=0.117	–	–	–	–	–	–
Mezurio	ost logRelF0 H1 H2 sma3nz amean	✓	F=1.596 η^2=0.03 [0.00, 1.00] p=0.193	–	–	–	–	–	–
Mezurio	ost logRelF0 H1 H2 sma3nz stddevNorm	✓	F=1.329 η^2=0.03 [0.00, 1.00] p=0.268	–	–	–	–	–	–
Mezurio	ost loudness sma3 amean	✓	F=1.547 η^2=0.03 [0.00, 1.00] p=0.205	–	–	–	–	–	–
Mezurio	ost loudness sma3 meanFallingSlope	✓	F=0.473 η^2=0.01 [0.00, 1.00] p=0.701	–	–	–	–	–	–
Mezurio	ost loudness sma3 meanRisingSlope	✓	F=0.374 η^2=0.01 [0.00, 1.00] p=0.772	–	–	–	–	–	–

Continued on next page

RMT	Feature	Log	Ancova	HC vs. PreAD	HC vs. ProAD	HC vs. MildAD	PreAD vs. ProAD	PreAD vs. MildAD	ProAD vs. MildAD
Mezurio	ost loudness sma3 pctlrange0 2	✓	F=2.902<br>η^2=0.06 [0.00, 1.00]<br>p=0.037	-0.18 [-0.69,0.32]<br>d=-0.26 [-0.71, 0.19]<br>p=1.0	0.30 [-0.13,0.72]<br>d=0.31 [-0.07, 0.69]<br>p=1.0	0.36 [-0.15,0.87]<br>d=0.46 [0.00, 0.92]<br>p=1.0	0.48 [-0.06,1.02]<br>d=0.45 [-0.04, 0.93]<br>p=1.0	0.54 [-0.06,1.14]<br>d=0.66 [0.10, 1.21]<br>p=1.0	0.06 [-0.48,0.60]<br>d=0.05 [-0.43, 0.54]<br>p=1.0
Mezurio	ost loudness sma3 percentile20 0	✓	F=0.329<br>η^2=0.01 [0.00, 1.00]<br>p=0.804	–	–	–	–	–	–
Mezurio	ost loudness sma3 percentile50 0	✓	F=3.136<br>η^2=0.06 [0.00, 1.00]<br>p=0.027	-0.26 [-0.63,0.11]<br>d=-0.41 [-0.86, 0.04]<br>p=1.0	0.18 [-0.13,0.50]<br>d=0.26 [-0.12, 0.64]<br>p=1.0	0.14 [-0.24,0.52]<br>d=0.19 [-0.26, 0.65]<br>p=1.0	0.45 [0.05,0.84]<br>d=0.63 [0.13, 1.11]<br>p=1.0	0.40 [-0.05,0.85]<br>d=0.54 [-0.01, 1.08]<br>p=1.0	-0.04 [-0.45,0.36]<br>d=-0.06 [-0.54, 0.43]<br>p=1.0
Mezurio	ost loudness sma3 percentile80 0	✓	F=2.296<br>η^2=0.05 [0.00, 1.00]<br>p=0.08	–	–	–	–	–	–
Mezurio	ost loudness sma3 stddevFallingSlope	✓	F=0.42<br>η^2=0.01 [0.00, 1.00]<br>p=0.739	–	–	–	–	–	–
Mezurio	ost loudness sma3 stddevNorm	✓	F=0.275<br>η^2=0.01 [0.00, 1.00]<br>p=0.843	–	–	–	–	–	–
Mezurio	ost loudness sma3 stddevRisingSlope	✓	F=0.253<br>η^2=0.01 [0.00, 1.00]<br>p=0.859	–	–	–	–	–	–
Mezurio	ost loudnessPeaksPerSec	×	F=6.902<br>η^2=0.12 [0.04, 1.00]<br>p<0.001	-0.14 [-0.46,0.17]<br>d=-0.30 [-0.75, 0.15]<br>p=1.0	0.29 [0.03,0.56]<br>d=0.57 [0.18, 0.96]<br>p=1.0	0.37 [0.05,0.69]<br>d=0.67 [0.20, 1.13]<br>p=0.631	0.44 [0.10,0.78]<br>d=0.84 [0.34, 1.34]<br>p=0.343	0.52 [0.14,0.90]<br>d=0.89 [0.32, 1.45]<br>p=0.126	0.08 [-0.26,0.42]<br>d=0.13 [-0.36, 0.61]<br>p=1.0
Mezurio	ost mfcc1 sma3 amean	✓	F=0.06<br>η^2=0.00 [0.00, 1.00]<br>p=0.981	–	–	–	–	–	–
Mezurio	ost mfcc1 sma3 stddevNorm	✓	F=0.644<br>η^2=0.01 [0.00, 1.00]<br>p=0.588	–	–	–	–	–	–
Mezurio	ost mfcc1V sma3nz amean	✓	F=1.605<br>η^2=0.03 [0.00, 1.00]<br>p=0.191	–	–	–	–	–	–
Mezurio	ost mfcc1V sma3nz stddevNorm	✓	F=2.241<br>η^2=0.04 [0.00, 1.00]<br>p=0.086	–	–	–	–	–	–
Mezurio	ost mfcc2 sma3 amean	✓	F=0.969<br>η^2=0.06 [0.00, 1.00]<br>p=0.415	–	–	–	–	–	–
Mezurio	ost mfcc2 sma3 stddevNorm	✓	F=1.462<br>η^2=0.08 [0.00, 1.00]<br>p=0.235	–	–	–	–	–	–
Mezurio	ost mfcc2V sma3nz amean	✓	F=1.385<br>η^2=0.05 [0.00, 1.00]<br>p=0.254	–	–	–	–	–	–
Mezurio	ost mfcc2V sma3nz stddevNorm	✓	F=1.131<br>η^2=0.04 [0.00, 1.00]<br>p=0.342	–	–	–	–	–	–
Mezurio	ost mfcc3 sma3 amean	×	F=0.568<br>η^2=0.01 [0.00, 1.00]<br>p=0.637	–	–	–	–	–	–
Mezurio	ost mfcc3 sma3 stddevNorm	✓	F=0.651<br>η^2=0.01 [0.00, 1.00]<br>p=0.583	–	–	–	–	–	–
Mezurio	ost mfcc3V sma3nz amean	×	F=0.56<br>η^2=0.01 [0.00, 1.00]<br>p=0.642	–	–	–	–	–	–

Continued on next page

RMT	Feature	Log	Ancova	HC vs. PreAD	HC vs. ProAD	HC vs. MildAD	PreAD vs. ProAD	PreAD vs. MildAD	ProAD vs. MildAD
Mezurio	ost mfcc3V sma3nz stddevNorm	✓	F=1.568<br>η^2=0.03 [0.00, 1.00]<br>p=0.2	–	–	–	–	–	–
Mezurio	ost mfcc4 sma3 amean	×	F=1.257<br>η^2=0.03 [0.00, 1.00]<br>p=0.292	–	–	–	–	–	–
Mezurio	ost mfcc4 sma3 stddevNorm	✓	F=0.405<br>η^2=0.03 [0.00, 1.00]<br>p=0.75	–	–	–	–	–	–
Mezurio	ost mfcc4V sma3nz amean	×	F=2.56<br>η^2=0.05 [0.00, 1.00]<br>p=0.057	–	–	–	–	–	–
Mezurio	ost mfcc4V sma3nz stddevNorm	✓	F=1.646<br>η^2=0.15 [0.00, 1.00]<br>p=0.2	–	–	–	–	–	–
Mezurio	ost shimmerLocaldB sma3nz amean	✓	F=1.722<br>η^2=0.03 [0.00, 1.00]<br>p=0.165	–	–	–	–	–	–
Mezurio	ost shimmerLocaldB sma3nz stddevNorm	✓	F=1.839<br>η^2=0.04 [0.00, 1.00]<br>p=0.143	–	–	–	–	–	–
Mezurio	ost slopeUV0 500 sma3nz amean	✓	F=0.857<br>η^2=0.02 [0.00, 1.00]<br>p=0.465	–	–	–	–	–	–
Mezurio	ost slopeUV500 1500 sma3nz amean	×	F=1.351<br>η^2=0.03 [0.00, 1.00]<br>p=0.26	–	–	–	–	–	–
Mezurio	ost slopeV0 500 sma3nz amean	×	F=2.127<br>η^2=0.04 [0.00, 1.00]<br>p=0.099	–	–	–	–	–	–
Mezurio	ost slopeV0 500 sma3nz stddevNorm	✓	F=1.538<br>η^2=0.03 [0.00, 1.00]<br>p=0.207	–	–	–	–	–	–
Mezurio	ost slopeV500 1500 sma3nz amean	×	F=1.198<br>η^2=0.02 [0.00, 1.00]<br>p=0.313	–	–	–	–	–	–
Mezurio	ost spectralFlux sma3 amean	✓	F=0.681<br>η^2=0.01 [0.00, 1.00]<br>p=0.565	–	–	–	–	–	–
Mezurio	ost spectralFlux sma3 stddevNorm	✓	F=3.733<br>η^2=0.07 [0.01, 1.00]<br>p=0.013	0.02 [-0.15,0.19]<br>d=0.08 [-0.37, 0.53]<br>p=1.0	-0.10 [-0.24,0.05]<br>d=-0.38 [-0.76, 0.01]<br>p=1.0	-0.19 [-0.36,-0.02]<br>d=-0.61 [-1.08, -0.15]<br>p=0.85	-0.12 [-0.30,0.06]<br>d=-0.50 [-0.98, -0.01]<br>p=1.0	-0.21 [-0.42,-0.01]<br>d=-0.65 [-1.20, -0.10]<br>p=1.0	-0.09 [-0.28,0.09]<br>d=-0.30 [-0.78, 0.19]<br>p=1.0
Mezurio	ost spectralFluxUV sma3nz amean	✓	F=0.394<br>η^2=0.01 [0.00, 1.00]<br>p=0.758	–	–	–	–	–	–
Mezurio	ost spectralFluxV sma3nz amean	✓	F=1.222<br>η^2=0.02 [0.00, 1.00]<br>p=0.304	–	–	–	–	–	–
Mezurio	ost spectralFluxV sma3nz stddevNorm	✓	F=1.871<br>η^2=0.04 [0.00, 1.00]<br>p=0.137	–	–	–	–	–	–
Physilog (DUAL)	avg DS	✓	F=7.846<br>η^2=0.19 [0.07, 1.00]<br>p<0.001	-0.66 [-1.62,0.30]<br>d=-0.42 [-0.96, 0.13]<br>p=1.0	-0.89 [-1.72,-0.06]<br>d=-0.62 [-1.10, -0.14]<br>p=1.0	-1.69 [-2.61,-0.77]<br>d=-1.04 [-1.58, -0.48]<br>p=0.002	-0.23 [-1.16,0.70]<br>d=-0.26 [-0.79, 0.27]<br>p=1.0	-1.03 [-2.05,-0.02]<br>d=-0.96 [-1.56, -0.35]<br>p=1.0	-0.80 [-1.70,0.09]<br>d=-0.79 [-1.31, -0.26]<br>p=1.0
Physilog (DUAL)	avg FFr	✓	F=3.28<br>η^2=0.07 [0.01, 1.00]<br>p=0.023	0.08 [-0.68,0.84]<br>d=0.06 [-0.41, 0.52]<br>p=1.0	-0.38 [-1.05,0.29]<br>d=-0.30 [-0.72, 0.11]<br>p=1.0	-0.74 [-1.46,-0.01]<br>d=-0.56 [-1.02, -0.11]<br>p=1.0	-0.46 [-1.21,0.29]<br>d=-0.37 [-0.84, 0.09]<br>p=1.0	-0.82 [-1.62,-0.01]<br>d=-0.63 [-1.14, -0.12]<br>p=1.0	-0.35 [-1.07,0.36]<br>d=-0.28 [-0.73, 0.16]<br>p=1.0

Continued on next page

RMT	Feature	Log	Ancova	HC vs. PreAD	HC vs. ProAD	HC vs. MildAD	PreAD vs. ProAD	PreAD vs. MildAD	ProAD vs. MildAD
Physilog (DUAL)	avg HSP	✓	F=0.487<br>η^2=0.03 [0.00, 1.00]<br>p=0.693	–	–	–	–	–	–
Physilog (DUAL)	avg LDr	✓	F=0.748<br>η^2=0.13 [0.00, 1.00]<br>p=0.54	–	–	–	–	–	–
Physilog (DUAL)	avg PUr	✓	F=2.902<br>η^2=0.29 [0.00, 1.00]<br>p=0.059	–	–	–	–	–	–
Physilog (DUAL)	avg PathLength	✓	F=3.599<br>η^2=0.11 [0.01, 1.00]<br>p=0.017	0.35 [-0.81,1.50]<br>d=0.29 [-0.29, 0.86]<br>p=1.0	-0.59 [-1.58,0.40]<br>d=-0.42 [-0.91, 0.08]<br>p=1.0	-0.97 [-1.98,0.04]<br>d=-0.57 [-1.08, -0.06]<br>p=1.0	-0.94 [-2.15,0.27]<br>d=-0.66 [-1.27, -0.04]<br>p=1.0	-1.32 [-2.55,-0.09]<br>d=-0.72 [-1.35, -0.09]<br>p=1.0	-0.38 [-1.46,0.69]<br>d=-0.20 [-0.74, 0.33]<br>p=1.0
Physilog (DUAL)	avg TOP	✓	F=0.908<br>η^2=0.07 [0.00, 1.00]<br>p=0.447	–	–	–	–	–	–
Physilog (DUAL)	avg cadence	✓	F=0.659<br>η^2=0.40 [0.00, 1.00]<br>p=0.63	–	–	–	–	–	–
Physilog (DUAL)	avg gct	✓	F=3.628<br>η^2=0.07 [0.01, 1.00]<br>p=0.015	-0.16 [-0.98,0.66]<br>d=-0.16 [-0.64, 0.32]<br>p=1.0	-0.45 [-1.15,0.24]<br>d=-0.38 [-0.79, 0.03]<br>p=1.0	-0.89 [-1.63,-0.16]<br>d=-0.60 [-1.04, -0.16]<br>p=0.446	-0.29 [-1.11,0.53]<br>d=-0.27 [-0.75, 0.21]<br>p=1.0	-0.73 [-1.59,0.12]<br>d=-0.50 [-1.00, 0.01]<br>p=1.0	-0.44 [-1.18,0.30]<br>d=-0.27 [-0.70, 0.17]<br>p=1.0
Physilog (DUAL)	avg peakswing	✓	F=0.648<br>η^2=0.14 [0.00, 1.00]<br>p=0.599	–	–	–	–	–	–
Physilog (DUAL)	avg slength	✓	F=1.187<br>η^2=0.08 [0.00, 1.00]<br>p=0.327	–	–	–	–	–	–
Physilog (DUAL)	avg speed	✓	F=1.938<br>η^2=0.42 [0.00, 1.00]<br>p=0.202	–	–	–	–	–	–
Physilog (DUAL)	avg stance	✓	F=10.015<br>η^2=0.23 [0.11, 1.00]<br>p<0.001	-0.27 [-1.09,0.55]<br>d=-0.21 [-0.74, 0.33]<br>p=1.0	-0.80 [-1.52,-0.09]<br>d=-0.71 [-1.19, -0.23]<br>p=1.0	-1.52 [-2.28,-0.75]<br>d=-1.13 [-1.67, -0.59]<br>p<0.001	-0.54 [-1.34,0.27]<br>d=-0.64 [-1.18, -0.10]<br>p=1.0	-1.25 [-2.10,-0.40]<br>d=-1.14 [-1.73, -0.53]<br>p=0.063	-0.71 [-1.46,0.04]<br>d=-0.73 [-1.23, -0.22]<br>p=1.0
Physilog (DUAL)	avg swidth	✓	F=3.737<br>η^2=0.15 [0.02, 1.00]<br>p=0.015	0.08 [-1.02,1.18]<br>d=0.06 [-0.51, 0.64]<br>p=1.0	0.46 [-0.68,1.60]<br>d=0.17 [-0.41, 0.76]<br>p=1.0	-1.12 [-2.28,0.05]<br>d=-0.77 [-1.39, -0.14]<br>p=1.0	0.38 [-0.88,1.63]<br>d=0.10 [-0.54, 0.75]<br>p=1.0	-1.20 [-2.47,0.08]<br>d=-0.70 [-1.38, 0.00]<br>p=1.0	-1.58 [-2.88,-0.27]<br>d=-0.70 [-1.39, 0.00]<br>p=0.749
Physilog (DUAL)	avg swing	✓	F=0.503<br>η^2=0.04 [0.00, 1.00]<br>p=0.683	–	–	–	–	–	–
Physilog (DUAL)	cv DS	✓	F=0.755<br>η^2=0.03 [0.00, 1.00]<br>p=0.524	–	–	–	–	–	–
Physilog (DUAL)	cv FFr	✓	F=0.304<br>η^2=0.02 [0.00, 1.00]<br>p=0.823	–	–	–	–	–	–
Physilog (DUAL)	cv HSP	✓	F=1.627<br>η^2=0.06 [0.00, 1.00]<br>p=0.191	–	–	–	–	–	–
Physilog (DUAL)	cv LDr	✓	F=1.652<br>η^2=0.06 [0.00, 1.00]<br>p=0.184	–	–	–	–	–	–
Physilog (DUAL)	cv PUr	✓	F=4.137<br>η^2=0.13 [0.02, 1.00]<br>p=0.009	0.26 [-1.02,1.53]<br>d=0.17 [-0.49, 0.83]<br>p=1.0	-0.45 [-1.47,0.56]<br>d=-0.22 [-0.74, 0.30]<br>p=1.0	-1.14 [-2.15,-0.13]<br>d=-0.77 [-1.32, -0.23]<br>p=0.825	-0.71 [-1.97,0.55]<br>d=-0.37 [-1.02, 0.29]<br>p=1.0	-1.40 [-2.66,-0.13]<br>d=-0.98 [-1.66, -0.28]<br>p=0.87	-0.69 [-1.68,0.31]<br>d=-0.46 [-0.98, 0.06]<br>p=1.0
Physilog (DUAL)	cv PathLength	✓	F=2.425<br>η^2=0.09 [0.00, 1.00]<br>p=0.073	–	–	–	–	–	–

Continued on next page

RMT	Feature	Log	Ancova	HC vs. PreAD	HC vs. ProAD	HC vs. MildAD	PreAD vs. ProAD	PreAD vs. MildAD	ProAD vs. MildAD
Physilog (DUAL)	cv TOP	✓	F=5.335<br>η^2=0.19 [0.05, 1.00]<br>p=0.002	1.00 [0.03,1.97]<br>d=1.08 [0.33, 1.82]<br>p=1.0	-0.16 [-0.92,0.59]<br>d=-0.15 [-0.69, 0.40]<br>p=1.0	-0.49 [-1.25,0.28]<br>d=-0.45 [-1.00, 0.11]<br>p=1.0	-1.16 [-2.14,-0.18]<br>d=-1.08 [-1.83, -0.32]<br>p=0.887	-1.48 [-2.47,-0.49]<br>d=-1.45 [-2.24, -0.65]<br>p=0.055	-0.32 [-1.10,0.45]<br>d=-0.27 [-0.84, 0.29]<br>p=1.0
Physilog (DUAL)	cv cadence	✓	F=4.884<br>η^2=0.12 [0.03, 1.00]<br>p=0.003	0.74 [-0.12,1.60]<br>d=0.65 [0.11, 1.19]<br>p=1.0	-0.01 [-0.77,0.74]<br>d=-0.02 [-0.48, 0.44]<br>p=1.0	-0.56 [-1.33,0.21]<br>d=-0.49 [-0.97, -0.01]<br>p=1.0	-0.75 [-1.63,0.12]<br>d=-0.52 [-1.06, 0.02]<br>p=1.0	-1.30 [-2.19,-0.41]<br>d=-0.97 [-1.54, -0.39]<br>p=0.063	-0.55 [-1.33,0.24]<br>d=-0.38 [-0.86, 0.10]<br>p=1.0
Physilog (DUAL)	cv gct	✓	F=1.518<br>η^2=0.04 [0.00, 1.00]<br>p=0.215	–	–	–	–	–	–
Physilog (DUAL)	cv peakswing	✓	F=4.1<br>η^2=0.12 [0.02, 1.00]<br>p=0.009	0.69 [-0.53,1.91]<br>d=0.51 [-0.13, 1.13]<br>p=1.0	0.04 [-0.90,0.99]<br>d=0.03 [-0.45, 0.51]<br>p=1.0	-0.88 [-1.86,0.10]<br>d=-0.72 [-1.23, -0.20]<br>p=1.0	-0.64 [-1.88,0.59]<br>d=-0.37 [-1.00, 0.26]<br>p=1.0	-1.56 [-2.82,-0.31]<br>d=-1.39 [-2.10, -0.68]<br>p=0.34	-0.92 [-1.91,0.07]<br>d=-0.61 [-1.12, -0.09]<br>p=1.0
Physilog (DUAL)	cv slength	✓	F=8.82<br>η^2=0.27 [0.12, 1.00]<br>p<0.001	1.12 [0.09,2.14]<br>d=1.09 [0.39, 1.78]<br>p=1.0	-0.24 [-1.09,0.62]<br>d=-0.20 [-0.74, 0.35]<br>p=1.0	-0.92 [-1.76,-0.09]<br>d=-0.77 [-1.32, -0.22]<br>p=0.872	-1.35 [-2.42,-0.28]<br>d=-1.14 [-1.86, -0.40]<br>p=0.496	-2.04 [-3.10,-0.98]<br>d=-1.70 [-2.47, -0.92]<br>p=0.001	-0.69 [-1.58,0.21]<br>d=-0.52 [-1.09, 0.06]<br>p=1.0
Physilog (DUAL)	cv speed	✓	F=5.186<br>η^2=0.15 [0.04, 1.00]<br>p=0.002	0.61 [-0.42,1.63]<br>d=0.49 [-0.16, 1.14]<br>p=1.0	-0.27 [-1.08,0.53]<br>d=-0.22 [-0.72, 0.28]<br>p=1.0	-0.87 [-1.67,-0.07]<br>d=-0.74 [-1.26, -0.22]<br>p=0.945	-0.88 [-1.94,0.17]<br>d=-0.61 [-1.27, 0.06]<br>p=1.0	-1.48 [-2.53,-0.42]<br>d=-1.09 [-1.79, -0.38]<br>p=0.104	-0.60 [-1.44,0.24]<br>d=-0.45 [-0.97, 0.08]<br>p=1.0
Physilog (DUAL)	cv stance	✓	F=3.89<br>η^2=0.13 [0.02, 1.00]<br>p=0.012	0.60 [-0.65,1.85]<br>d=0.47 [-0.20, 1.13]<br>p=1.0	-0.57 [-1.60,0.45]<br>d=-0.39 [-0.93, 0.16]<br>p=1.0	-0.87 [-1.87,0.14]<br>d=-0.69 [-1.24, -0.15]<br>p=1.0	-1.17 [-2.45,0.10]<br>d=-0.76 [-1.45, -0.06]<br>p=1.0	-1.47 [-2.72,-0.21]<br>d=-1.20 [-1.90, -0.48]<br>p=0.588	-0.30 [-1.33,0.74]<br>d=-0.20 [-0.75, 0.34]<br>p=1.0
Physilog (DUAL)	cv swidth	✓	F=0.296<br>η^2=0.02 [0.00, 1.00]<br>p=0.828	–	–	–	–	–	–
Physilog (DUAL)	cv swing	✓	F=4.105<br>η^2=0.13 [0.02, 1.00]<br>p=0.009	0.65 [-0.60,1.90]<br>d=0.49 [-0.14, 1.11]<br>p=1.0	-0.64 [-1.69,0.41]<br>d=-0.43 [-0.95, 0.10]<br>p=1.0	-0.84 [-1.87,0.18]<br>d=-0.56 [-1.07, -0.04]<br>p=1.0	-1.29 [-2.56,-0.01]<br>d=-0.86 [-1.51, -0.19]<br>p=1.0	-1.49 [-2.75,-0.24]<br>d=-0.97 [-1.62, -0.32]<br>p=0.49	-0.20 [-1.26,0.85]<br>d=-0.12 [-0.64, 0.40]<br>p=1.0
Physilog (TUG)	NGaitCycles	✓	F=5.535<br>η^2=0.10 [0.03, 1.00]<br>p=0.001	0.07 [-0.09,0.22]<br>d=0.23 [-0.22, 0.68]<br>p=1.0	-0.01 [-0.14,0.13]<br>d=-0.01 [-0.40, 0.39]<br>p=1.0	-0.17 [-0.31,-0.02]<br>d=-0.48 [-0.89, -0.06]<br>p=0.631	-0.07 [-0.23,0.09]<br>d=-0.21 [-0.66, 0.24]<br>p=1.0	-0.23 [-0.39,-0.07]<br>d=-0.63 [-1.11, -0.16]<br>p=0.072	-0.16 [-0.30,-0.02]<br>d=-0.43 [-0.85, -0.02]<br>p=1.0
Physilog (TUG)	cadence	✓	F=2.114<br>η^2=0.04 [0.00, 1.00]<br>p=0.101	–	–	–	–	–	–
Physilog (TUG)	gaitspeed	×	F=6.072<br>η^2=0.11 [0.03, 1.00]<br>p<0.001	0.00 [-0.11,0.11]<br>d=-0.01 [-0.46, 0.44]<br>p=1.0	0.01 [-0.08,0.11]<br>d=0.08 [-0.31, 0.48]<br>p=1.0	0.15 [0.04,0.25]<br>d=0.56 [0.14, 0.98]<br>p=0.079	0.02 [-0.10,0.13]<br>d=0.09 [-0.36, 0.54]<br>p=1.0	0.15 [0.03,0.26]<br>d=0.53 [0.05, 1.00]<br>p=0.245	0.13 [0.03,0.23]<br>d=0.45 [0.04, 0.87]<br>p=0.371
Physilog (TUG)	sist angle range	✓	F=1.866<br>η^2=0.04 [0.00, 1.00]<br>p=0.138	–	–	–	–	–	–
Physilog (TUG)	sist duration	✓	F=2.256<br>η^2=0.05 [0.00, 1.00]<br>p=0.084	–	–	–	–	–	–
Physilog (TUG)	total time	✓	F=0.577<br>η^2=0.01 [0.00, 1.00]<br>p=0.631	–	–	–	–	–	–
Physilog (TUG)	turn duration	✓	F=2.448<br>η^2=0.05 [0.00, 1.00]<br>p=0.066	–	–	–	–	–	–
Physilog (TUG)	turnsi duration	✓	F=0.991<br>η^2=0.02 [0.00, 1.00]<br>p=0.399	–	–	–	–	–	–

C.2 Additional Information: Combination of FDS and RMT data

Supplementary Figure C.1 offers more detailed results compared to Figure 3 by including data from models that incorporate both RMT and FDS. In some cases (HC vs. MildAD and PreAD vs. MildAD), the fusion of these measures led to better performance compared to the FDS-only model. However, the improvement was marginal and not present in all scenarios, suggesting that the simple combination is not beneficial. Supplementary Table C.2 shows the average AUROCs alongside its standard error for better comparability with other studies.

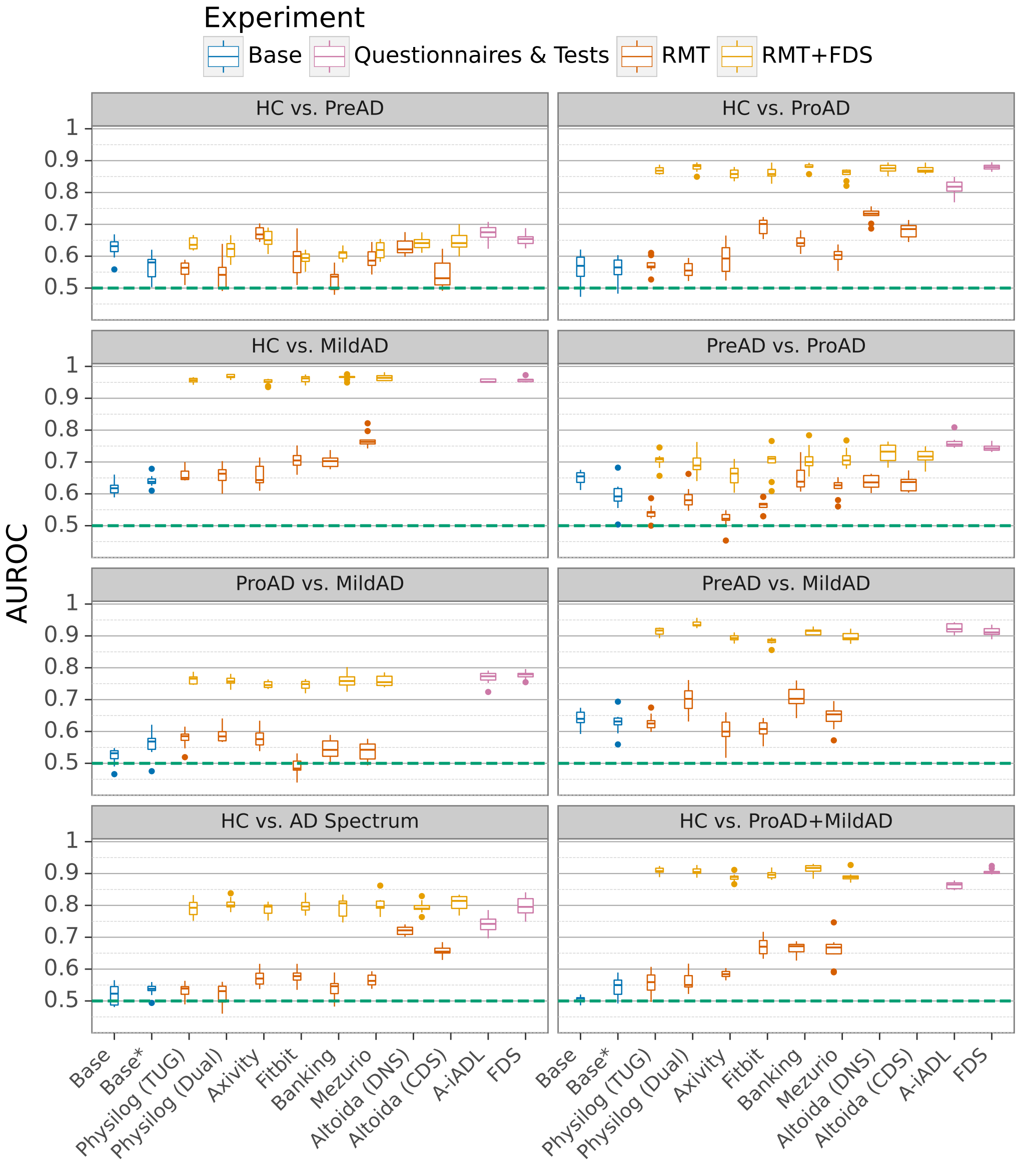

Supplementary Figure C.1: **Discriminative abilities of different RMTs and their performance comparison across different disease stages.** The figure depicts the Area Under the Receiver Operator Characteristic (AUROC). As we focused on optimal performance rather than specific classifiers to emphasize the highest discrimination ability, we show the AUROC only for the best-performing machine learning model in each experiment. The red and green boxes represent the base models, while the purple boxes illustrate the models trained solely on RMT data. The blue boxes depict the performance achieved when combined with questionnaire and test-based assessments (A-iADL and the composite score derived from multiple questionnaires).

Supplementary Table C.2: **Comparative Analysis of RMTs' Discriminative Abilities Across Various Disease Stages.** This table presents the Area Under the Receiver Operating Characteristic (AUROC). To highlight the peak discrimination ability, we focused on optimal performance rather than specific classifiers, displaying the AUROC only for the top-performing machine learning model in each experiment. The table includes the average AUROC across ten repetitions, along with the standard error.

Type	HC vs. PreAD	HC vs. ProAD	HC vs. MildAD	PreAD vs. ProAD	ProAD vs. MildAD	PreAD vs. MildAD	HC vs. AD Spectrum	HC vs. ProAD + MildAD
Base (Base)	62.7 (1.0)	56.2 (1.5)	61.8 (0.7)	65.1 (0.7)	52.1 (0.8)	63.9 (0.8)	52.0 (1.0)	50.4 (0.3)
Base* (Base*)	56.7 (1.2)	55.9 (1.2)	64.0 (0.6)	59.4 (1.5)	56.2 (1.3)	62.9 (1.1)	53.6 (0.6)	54.6 (1.0)
A-iADL (RMT)	67.2 (0.8)	81.6 (0.8)	95.5 (0.2)	76.1 (0.6)	76.9 (0.6)	92.4 (0.5)	74.0 (0.8)	86.3 (0.3)
Altoida (CDS) (RMT)	54.6 (1.5)	68.1 (0.7)	-	63.4 (0.8)	-	-	65.8 (0.5)	-
Altoida (DNS) (RMT)	62.9 (0.8)	73.0 (0.7)	-	63.7 (0.7)	-	-	72.1 (0.4)	-
Axivity (RMT)	67.1 (0.6)	59.3 (1.6)	65.7 (1.1)	51.9 (0.9)	58.0 (1.0)	60.0 (1.4)	57.4 (0.9)	58.5 (0.4)
Banking (RMT)	52.4 (1.1)	64.3 (0.7)	70.1 (0.6)	64.9 (1.2)	54.6 (1.0)	70.6 (1.1)	54.0 (0.9)	66.5 (0.6)
FDS (RMT)	65.4 (0.7)	87.9 (0.3)	95.7 (0.2)	74.4 (0.3)	77.7 (0.4)	91.3 (0.4)	79.8 (1.0)	90.5 (0.3)
Fitbit (RMT)	59.0 (1.7)	69.3 (0.8)	70.4 (0.9)	56.3 (0.7)	48.8 (1.0)	60.8 (0.9)	57.5 (0.7)	67.1 (0.9)
Mezurio (RMT)	59.2 (1.0)	60.0 (0.8)	76.9 (0.7)	62.0 (0.9)	53.8 (0.9)	64.6 (1.2)	56.5 (0.6)	66.0 (1.4)
Physilog (Dual) (RMT)	54.8 (1.7)	55.7 (0.8)	66.0 (1.0)	58.5 (1.1)	59.0 (0.8)	69.9 (1.4)	51.9 (1.1)	56.0 (1.0)
Physilog (TUG) (RMT)	55.9 (0.8)	57.1 (0.7)	65.9 (0.6)	54.0 (0.7)	57.8 (0.9)	62.8 (0.7)	53.4 (0.7)	55.8 (1.1)
A-iADL (RMT+FDS)	-	-	-	-	-	-	-	-
Altoida (CDS) (RMT+FDS)	64.8 (1.0)	87.2 (0.4)	-	71.7 (0.7)	-	-	80.8 (0.7)	-
Altoida (DNS) (RMT+FDS)	64.2 (0.7)	87.6 (0.4)	-	72.8 (0.9)	-	-	79.4 (0.6)	-
Axivity (RMT+FDS)	65.3 (0.9)	85.9 (0.5)	95.1 (0.3)	65.9 (1.0)	74.7 (0.3)	89.3 (0.4)	78.9 (0.6)	88.7 (0.4)
Banking (RMT+FDS)	60.6 (0.5)	88.1 (0.3)	96.6 (0.2)	70.6 (1.3)	75.9 (0.7)	91.3 (0.3)	79.4 (1.0)	91.4 (0.5)
FDS (RMT+FDS)	-	-	-	-	-	-	-	-
Fitbit (RMT+FDS)	59.3 (0.6)	86.1 (0.6)	96.0 (0.3)	69.8 (1.4)	74.5 (0.5)	88.3 (0.4)	79.8 (0.7)	89.7 (0.4)
Mezurio (RMT+FDS)	61.9 (0.8)	85.8 (0.5)	96.4 (0.3)	71.1 (0.9)	75.8 (0.5)	89.7 (0.4)	80.2 (0.8)	89.1 (0.5)
Physilog (Dual) (RMT+FDS)	61.8 (1.0)	87.9 (0.4)	96.8 (0.2)	69.5 (1.1)	75.8 (0.4)	93.8 (0.3)	80.4 (0.6)	90.6 (0.4)
Physilog (TUG) (RMT+FDS)	64.0 (0.6)	87.0 (0.4)	95.6 (0.2)	70.5 (0.7)	76.4 (0.5)	91.4 (0.4)	79.0 (0.9)	90.9 (0.3)

C.2.1 Feature Importance Analysis

Supplementary Figures C.2 to C.4 depict the most important features of those models which performed best in our ML-based analysis. While the main text features only healthy controls versus prodromal and mild AD, the following figures provide information on the remaining comparisons. However, we do not include any results for prodromal versus mild AD, as all models failed in that task.

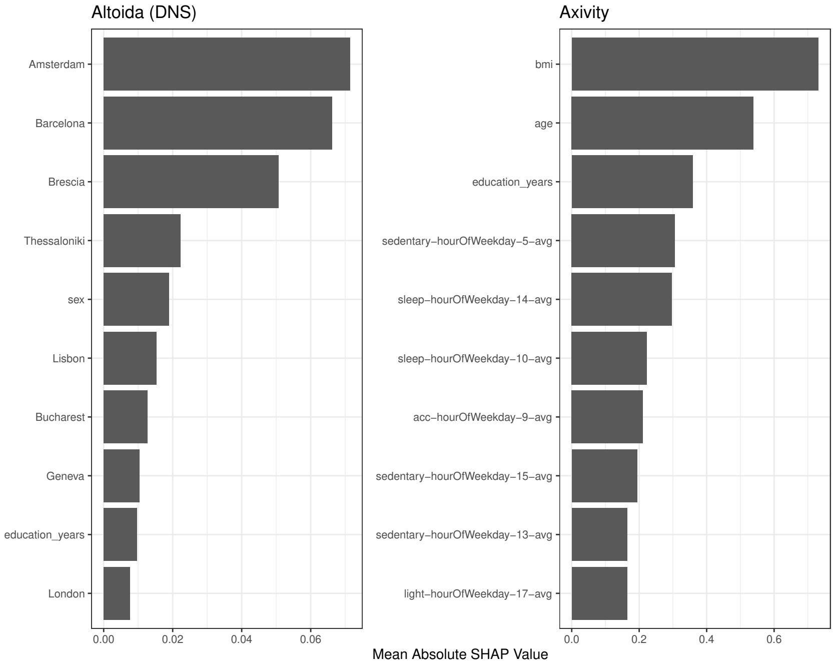

Supplementary Figure C.2: **Distinguishing Features for HC vs. PreAD:** Depicted are the mean absolute SHAP values of the ten most important features.

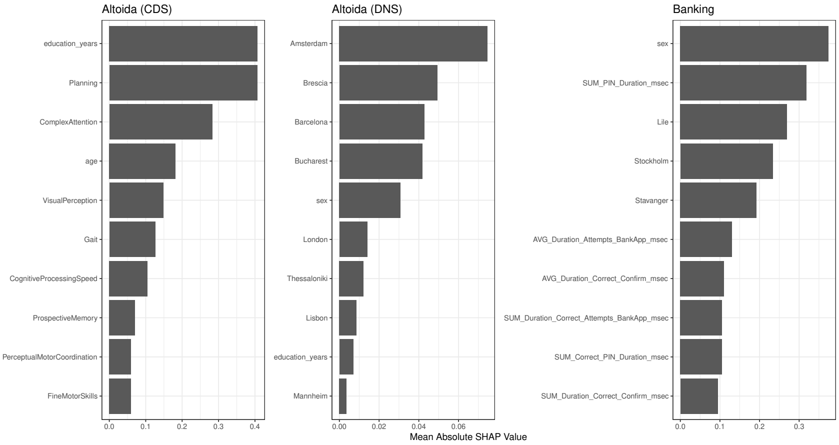

Supplementary Figure C.3: **Distinguishing Features for PreAD vs. ProAD:** Depicted are the mean absolute SHAP values of the ten most important features.

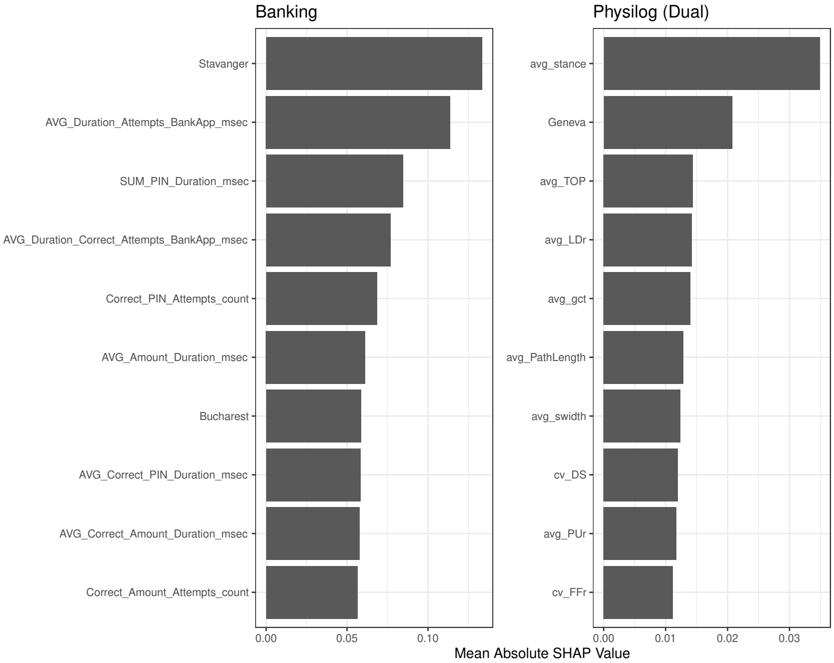

Supplementary Figure C.4: **Distinguishing Features for PreAD vs. MildAD:** Depicted are the mean absolute SHAP values of the ten most important features.

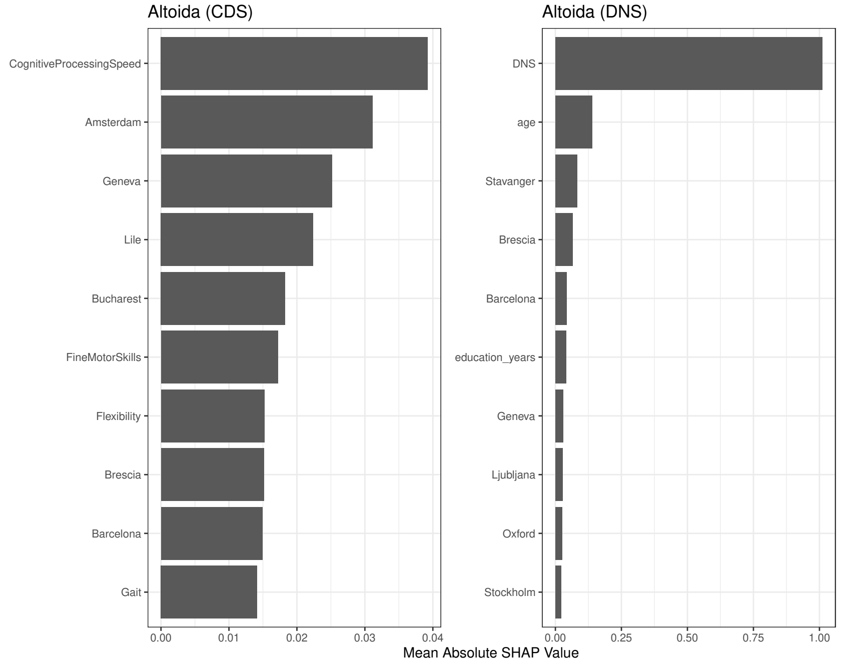

Supplementary Figure C.5: **Distinguishing Features for HC vs. AD Spectrum:** Depicted are the mean absolute SHAP values of the ten most important features.

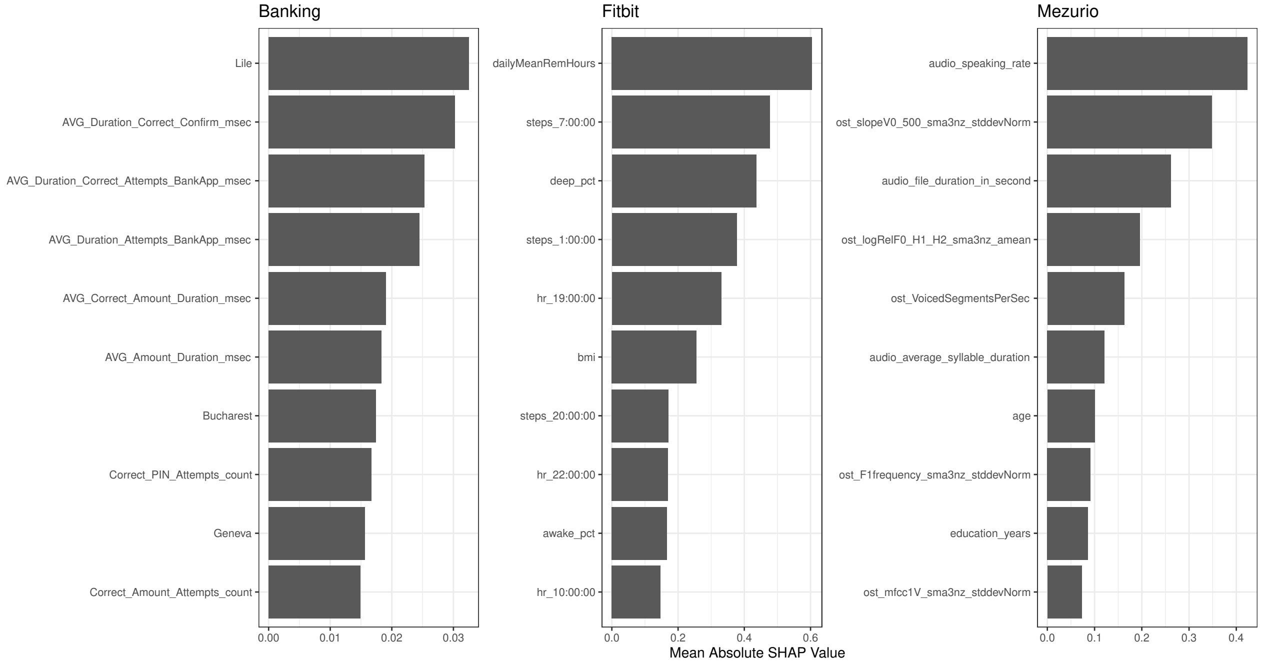

Supplementary Figure C.6: **Distinguishing Features for HC vs. ProAD+MildAD:** Depicted are the mean absolute SHAP values of the ten most important features.

C.3 Alternative metric: Area under the precision-recall curve

The results in Supplementary Figure C.7 present an alternative metric, the Area Under the Precision-Recall curve (AUPR). This metric is particularly informative when handling imbalanced data, offering more detailed insights compared to AUROC values. Specifically, in comparisons involving the slightly underrepresented preclinical AD group, AUPR provides valuable insights. Overall, these findings are consistent with the results from the AUROC-based analysis. Detailed observations are as follows:

HC vs. PreAD

A-iADL and FDS-based models outperform the Base models (Base= 55.2; Base*= 62.0), indicating their effectiveness in identifying preclinical AD participants(A-iADL= 66.7, FDS= 69.6). Slight improvements observed for Axivity (66.3) and the Altoida app (DNS) (62.6), although the effects are small.

HC vs. ProAD

Similar to the prior analysis, the A-iADL and FDS-based models exhibit excellent performance (A-iADL= 86.7, FDS= 91.2). The best RMTs are Altoida (CDS= 76.5, DNS= 79.9), Fitbit (76.6), and the Banking app (72.8), while other RMTs show performances close to the Base models.

HC vs. MildAD

A-iADL and FDS models achieve near-perfect AUPRs (A-iADL= 96.3, FDS= 96.8). The Mezurio-based model (81.7) shows significantly better results than the Base models. Additionally, the Banking app (75.1) and Fitbit-based models (75.2) also show a demonstrate effect, although smaller in magnitude.

PreAD vs. ProAD

Most models show only slight differences in this comparison. A-iADL (88.0) and FDS-based models (87.4) perform better, with a less pronounced effect for the Banking (83.6) and Altoida app-based models (CDS= 82.1, DNS= 82.4).

ProAD vs. MildAD

In this comparison, most models perform similarly to the Base models, except for the A-iADL (82.7) and FDS-based models (82.7), which show significantly better performance.

PreAD vs. MildAD

A-iADL and FDS-based models achieve near-perfect AUPRs (A-iADL= 96.0, FDS= 95.2), while the performance of the RMT-based models is close to the Base models.

HC vs. AD Spectrum

In this case, most models perform similarly to the Base model. Altoida (CDS+DNS= 87.8, 89.5) as well as A-iADL (89.8) and FDS (92.2) perform slightly better.

HC vs. ProAD+MildAD

As before, most models match the Base model performance. Mezurio (83.5), Banking (84.5), and Fitbit (84.6) offer slightly better performance, with A-iADL (93.6) and FDS (95.4) significantly outperforming the Base models.

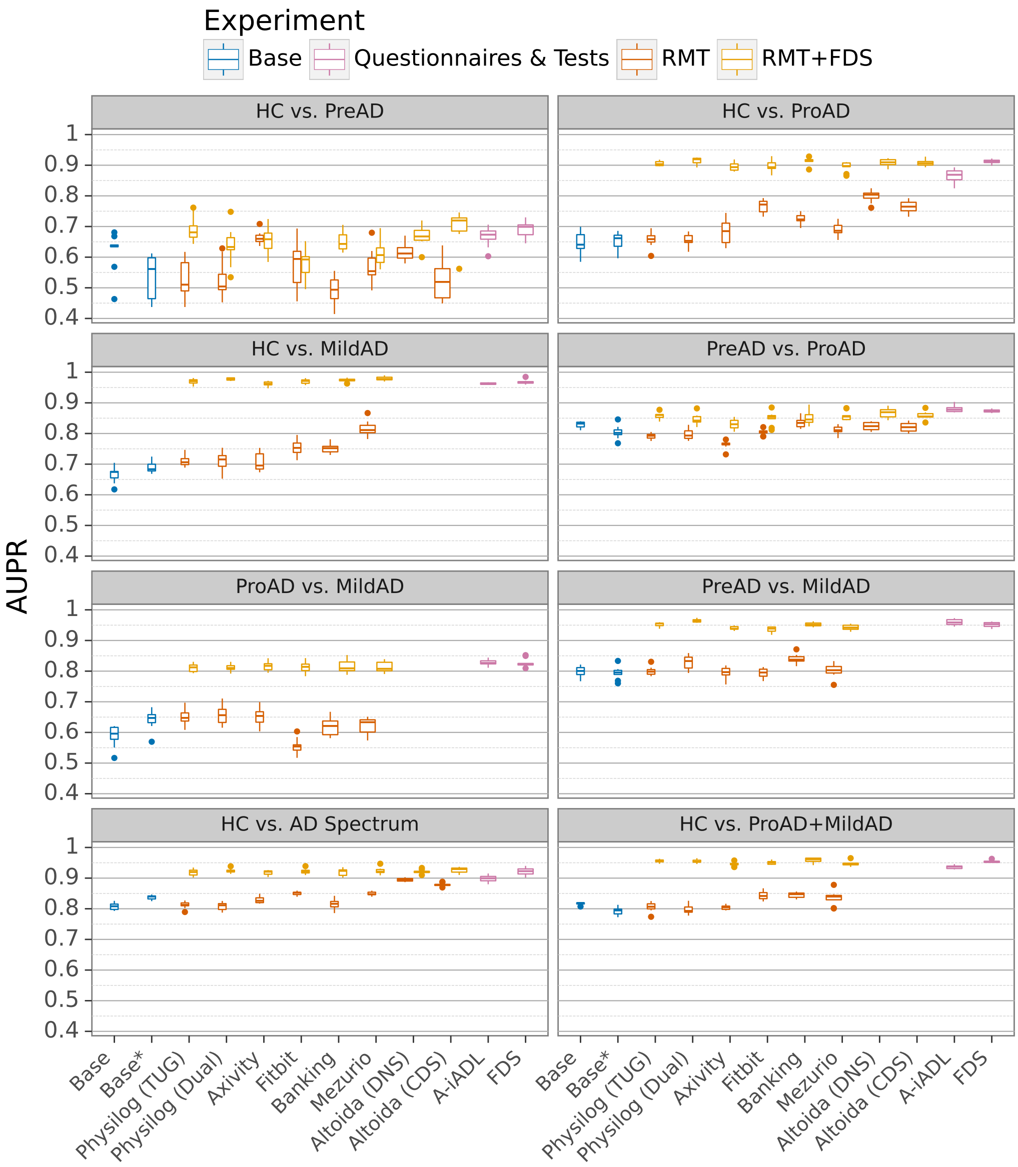

Supplementary Figure C.7: **Discriminative abilities of different RMTs and their performance comparison across different disease stages.** The figure presents the Area Under the Precision-Recall curve (AUPR) for the best-performing model in each experiment. Unlike the AUROC, the AUPR provides a more robust performance estimate in imbalanced scenarios. The red and green boxes represent the base models, while the purple boxes illustrate the models trained solely on RMT data. The blue boxes depict the performance achieved when combined with questionnaire and test-based assessments (A-iADL and the composite score derived from multiple questionnaires).

Supplementary Table C.3: **Comparative Analysis of RMTs' Discriminative Abilities Across Various Disease Stages.** This table presents the Area Under the Precision-Recall Curve. To highlight the peak discrimination ability, we focused on optimal performance rather than specific classifiers, displaying the AUPR only for the top-performing machine learning model in each experiment. The table includes the average AUROC across ten repetitions, along with the standard error.

Type	HC vs. PreAD	HC vs. ProAD	HC vs. MildAD	PreAD vs. ProAD	ProAD vs. MildAD	PreAD vs. MildAD	HC vs. AD Spectrum	HC vs. ProAD + MildAD
Base (Base)	62.0 (2.0)	64.6 (1.2)	66.6 (0.8)	82.9 (0.3)	66.1 (2.1)	79.8 (0.5)	84.9 (0.0)	81.7 (0.1)
Base* (Base*)	55.2 (1.6)	65.1 (1.0)	69.0 (0.6)	80.5 (0.5)	64.1 (1.0)	79.4 (0.6)	84.9 (0.0)	81.4 (0.1)
A-iADL (RMT)	66.7 (1.0)	86.7 (0.3)	96.3 (0.1)	88.0 (0.3)	82.7 (0.3)	96.0 (0.3)	89.8 (0.3)	93.6 (0.2)
Altoida (CDS) (RMT)	55.2 (1.3)	76.5 (0.6)	-	82.1 (0.5)	-	-	87.8 (0.2)	-
Altoida (DNS) (RMT)	62.6 (1.6)	79.9 (0.6)	-	82.4 (0.4)	-	-	89.5 (0.2)	-
Axivity (RMT)	66.3 (0.6)	68.4 (1.3)	70.7 (0.9)	79.8 (0.4)	65.2 (0.9)	79.5 (0.6)	84.5 (0.2)	80.4 (0.2)
Banking (RMT)	49.2 (2.2)	72.8 (0.8)	75.1 (0.5)	83.6 (0.5)	63.5 (2.1)	84.0 (0.5)	84.5 (0.1)	84.5 (0.3)
FDS (RMT)	69.6 (1.1)	91.2 (0.2)	96.8 (0.2)	87.4 (0.1)	82.7 (0.4)	95.2 (0.3)	92.2 (0.4)	95.4 (0.1)
Fitbit (RMT)	57.3 (2.4)	76.6 (0.7)	75.2 (0.8)	80.5 (0.3)	56.2 (1.1)	79.4 (0.5)	85.1 (0.1)	84.6 (0.3)
Mezurio (RMT)	57.0 (1.7)	68.8 (0.7)	81.7 (0.8)	81.2 (0.4)	62.2 (0.8)	80.2 (0.7)	84.9 (0.2)	83.5 (0.7)
Physilog (Dual) (RMT)	54.1 (2.0)	65.5 (0.7)	71.0 (0.9)	79.7 (0.5)	65.8 (1.0)	82.9 (0.7)	84.7 (0.1)	81.2 (0.3)
Physilog (TUG) (RMT)	56.8 (2.6)	65.8 (0.8)	71.0 (0.5)	79.1 (0.3)	65.0 (0.8)	80.0 (0.4)	84.8 (0.1)	80.8 (0.3)
A-iADL (RMT+FDS)	-	-	-	-	-	-	-	-
Altoida (CDS) (RMT+FDS)	69.8 (1.7)	90.8 (0.3)	-	85.8 (0.4)	-	-	92.6 (0.3)	-
Altoida (DNS) (RMT+FDS)	67.5 (1.2)	90.9 (0.4)	-	86.7 (0.5)	-	-	92.1 (0.2)	-
Axivity (RMT+FDS)	65.5 (1.3)	90.6 (0.5)	96.2 (0.2)	85.0 (0.6)	81.6 (0.5)	94.3 (0.6)	91.8 (0.2)	94.5 (0.2)
Banking (RMT+FDS)	65.1 (0.9)	91.4 (0.4)	97.4 (0.2)	85.1 (0.7)	81.6 (0.7)	95.2 (0.2)	92.0 (0.4)	95.9 (0.3)
FDS (RMT+FDS)	-	-	-	-	-	-	-	-
Fitbit (RMT+FDS)	58.1 (1.6)	91.9 (0.4)	97.0 (0.2)	85.0 (0.7)	81.2 (0.6)	93.7 (0.6)	92.2 (0.3)	95.1 (0.2)
Mezurio (RMT+FDS)	62.5 (1.8)	91.1 (0.4)	97.9 (0.2)	85.7 (0.5)	81.7 (0.7)	94.3 (0.3)	92.3 (0.3)	94.7 (0.2)
Physilog (Dual) (RMT+FDS)	63.6 (1.9)	91.5 (0.3)	97.7 (0.1)	85.5 (0.4)	81.1 (0.4)	96.7 (0.4)	92.4 (0.2)	95.5 (0.2)
Physilog (TUG) (RMT+FDS)	69.1 (1.2)	90.7 (0.5)	97.0 (0.3)	85.8 (0.3)	81.4 (0.6)	95.2 (0.2)	91.8 (0.4)	95.6 (0.2)

C.4 Model specific results for each RMT

Supplementary Figures C.8 to C.17 provide detailed results for each RMT or questionnaire. Unlike the Results section, which focuses on the best-performing model, these plots include outcomes for all trained models: LR, DT, RF, and XGBoost. These extended results are listed to allow readers to understand the exact results that were achieved per ML model.

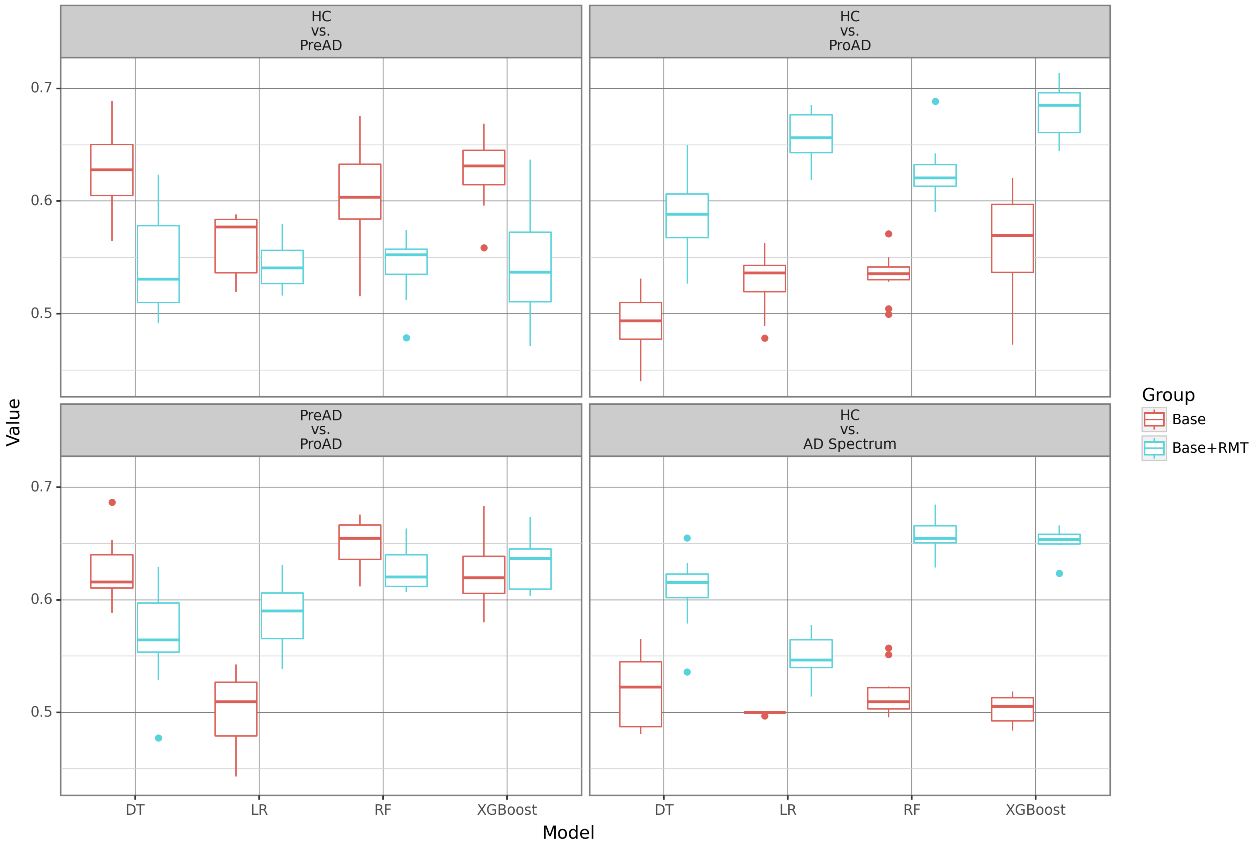

Supplementary Figure C.8: **Comparative Analysis of AUROCs for the Altoida (CDS) Data.** This figure displays boxplots of the Area Under the Receiver Operating Characteristic (AUROC) for Logistic Regression (LR), Decision Tree (DT), Random Forest (RF), and XGBoost models. Each model is assessed using two distinct feature sets: Base and RMT, as elaborated in the Methods section. The facet plots represent the binary classification between the different disease stages.

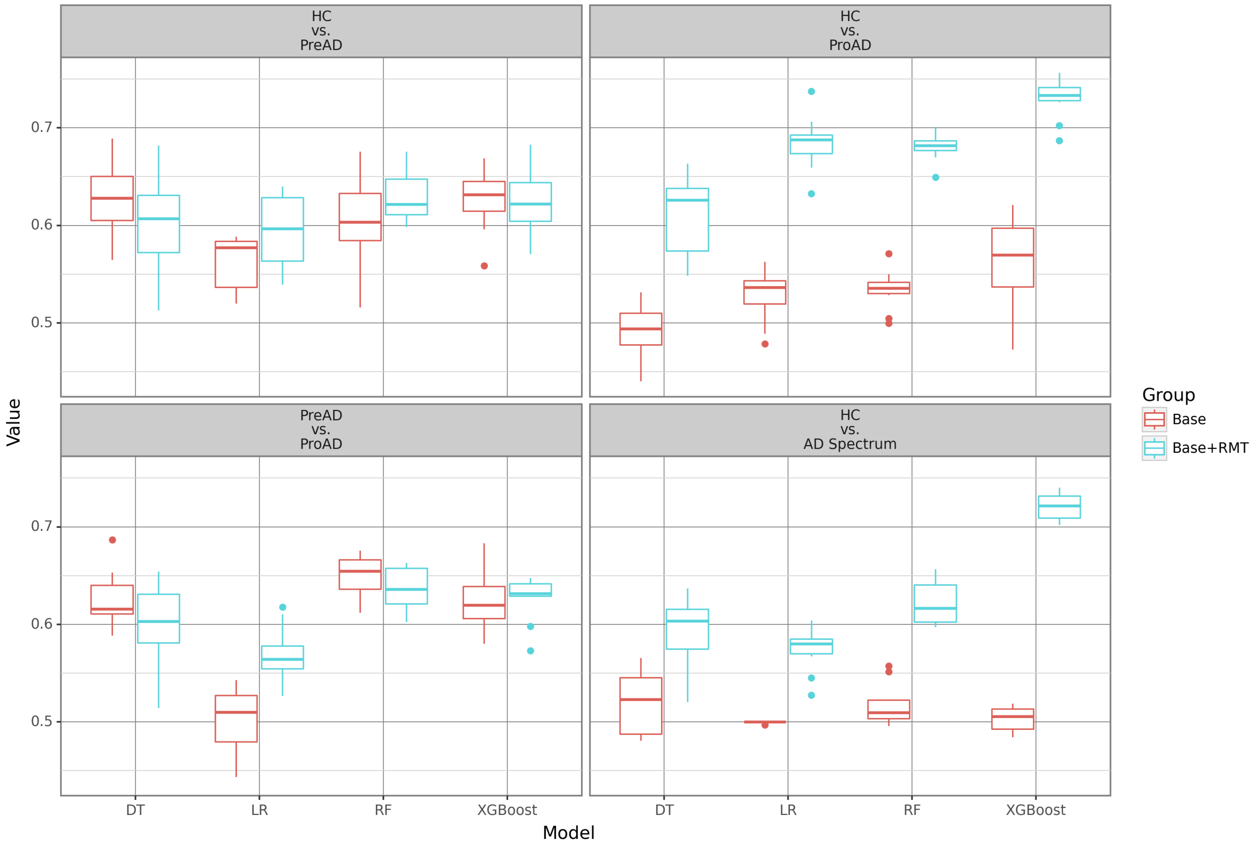

Supplementary Figure C.9: **Comparative Analysis of AUROCs for the Altoida (DNS) Data.** This figure displays boxplots of the Area Under the Receiver Operating Characteristic (AUROC) for Logistic Regression (LR), Decision Tree (DT), Random Forest (RF), and XGBoost models. Each model is assessed using two distinct feature sets: Base and RMT, as elaborated in the Methods section. The facet plots represent the binary classification between the different disease stages.

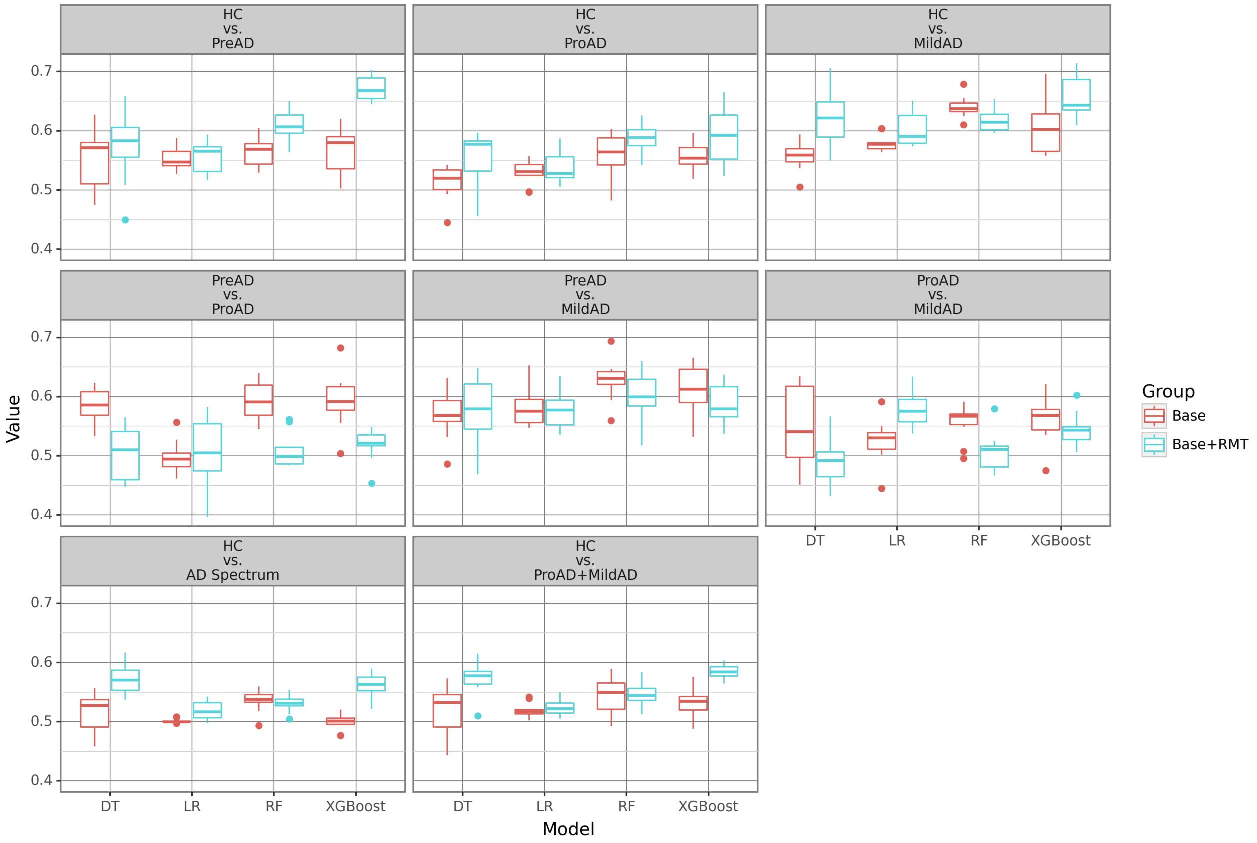

Supplementary Figure C.10: **Comparative Analysis of AUROCs for the Axivity Data.** This figure displays boxplots of the Area Under the Receiver Operating Characteristic (AUROC) for Logistic Regression (LR), Decision Tree (DT), Random Forest (RF), and XGBoost models. Each model is assessed using two distinct feature sets: Base and RMT, as elaborated in the Methods section. The facet plots represent the binary classification between the different disease stages.

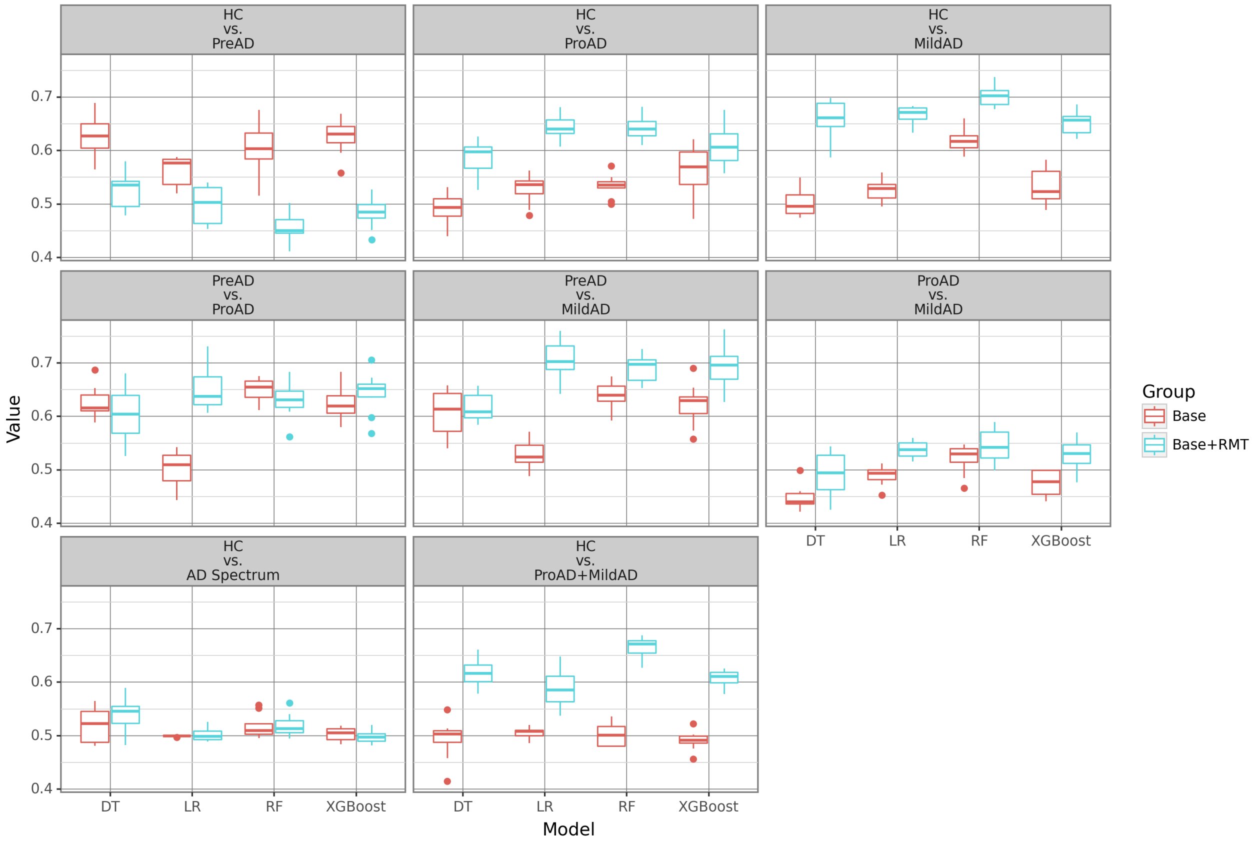

Supplementary Figure C.11: **Comparative Analysis of AUROCs for the Banking App Data.** This figure displays boxplots of the Area Under the Receiver Operating Characteristic (AUROC) for Logistic Regression (LR), Decision Tree (DT), Random Forest (RF), and XGBoost models. Each model is assessed using two distinct feature sets: Base and RMT, as elaborated in the Methods section. The facet plots represent the binary classification between the different disease stages.

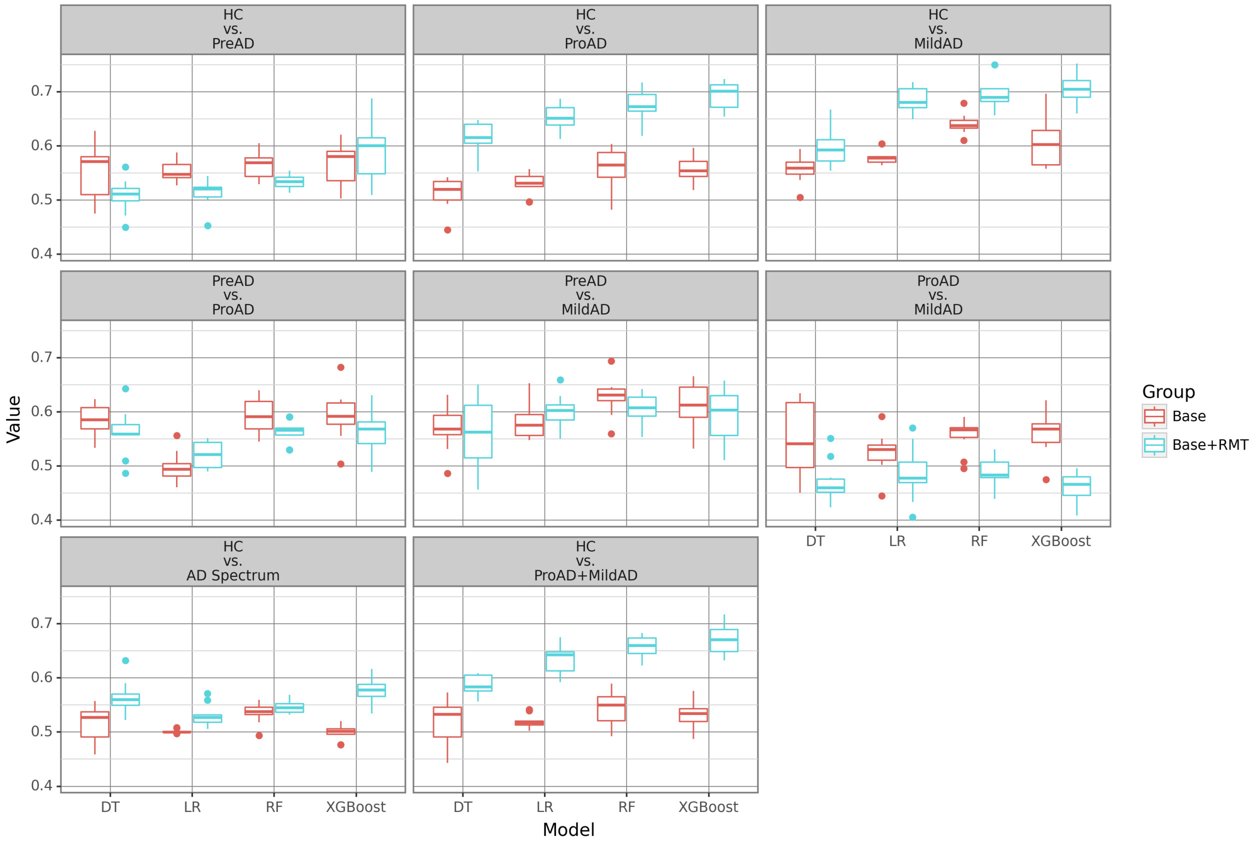

Supplementary Figure C.12: **Comparative Analysis of AUROCs for the Fitbit Data.** This figure displays boxplots of the Area Under the Receiver Operating Characteristic (AUROC) for Logistic Regression (LR), Decision Tree (DT), Random Forest (RF), and XGBoost models. Each model is assessed using two distinct feature sets: Base and RMT, as elaborated in the Methods section. The facet plots represent the binary classification between the different disease stages.

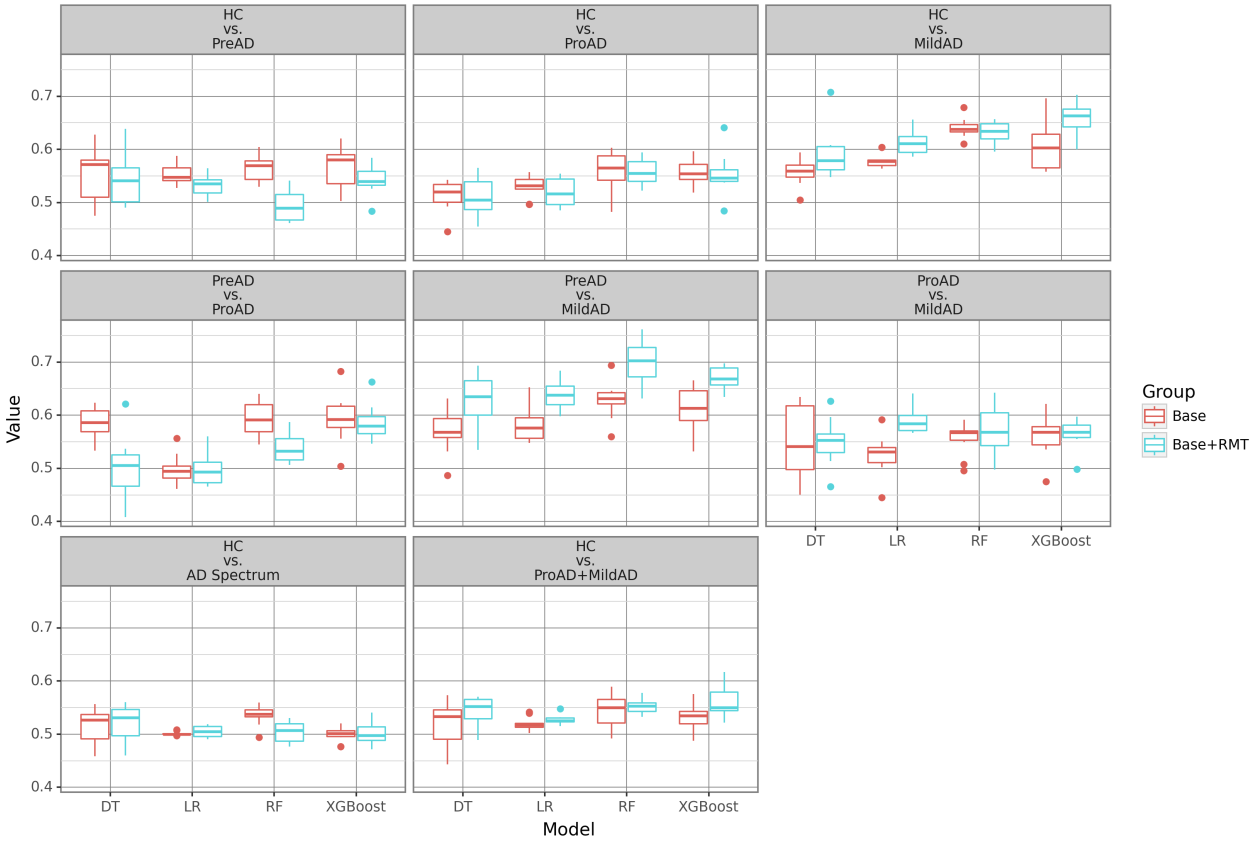

Supplementary Figure C.13: Comparative Analysis of AUROCs for the Physilog (Dual) Data. This figure displays boxplots of the Area Under the Receiver Operating Characteristic (AUROC) for Logistic Regression (LR), Decision Tree (DT), Random Forest (RF), and XGBoost models. Each model is assessed using two distinct feature sets: Base and RMT, as elaborated in the Methods section. The facet plots represent the binary classification between the different disease stages.

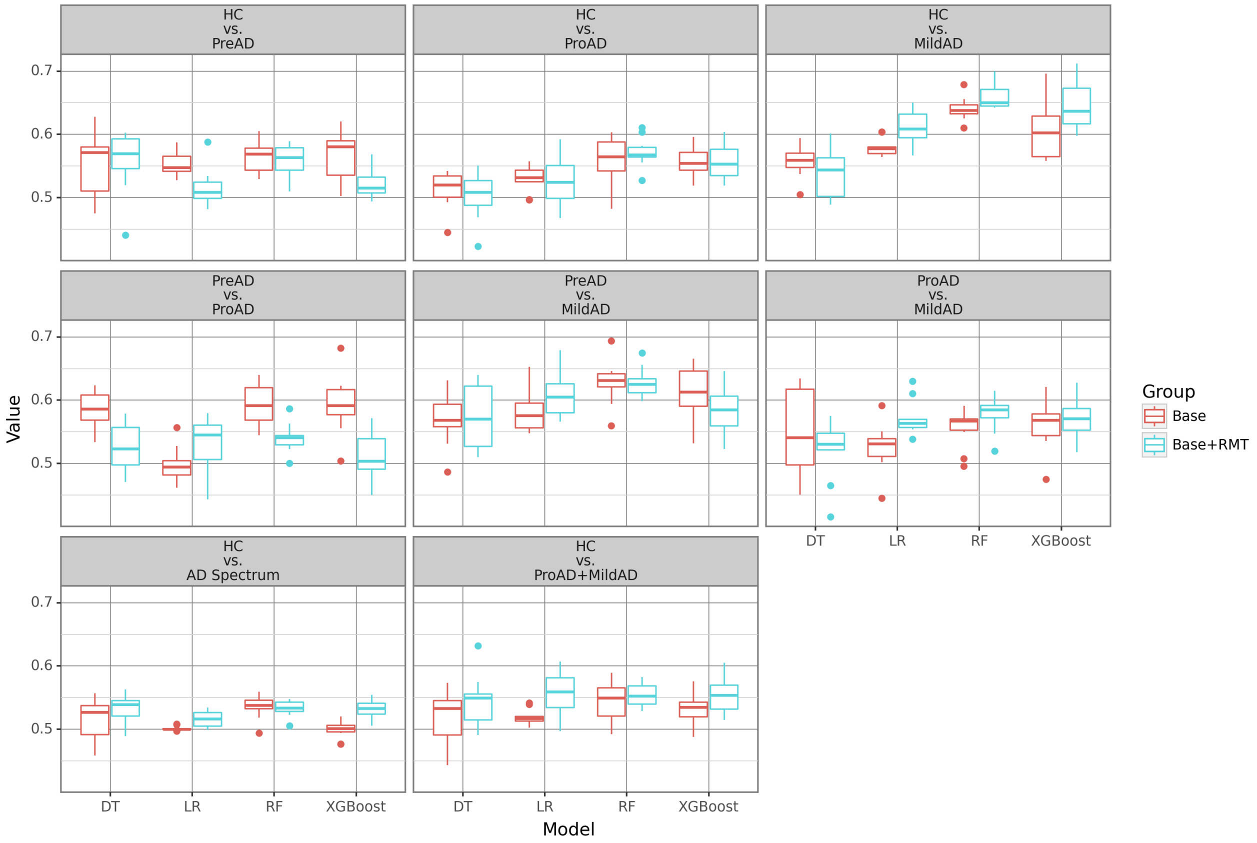

Supplementary Figure C.14: **Comparative Analysis of AUROCs for the Physilog (TUG) Data.** This figure displays boxplots of the Area Under the Receiver Operating Characteristic (AUROC) for Logistic Regression (LR), Decision Tree (DT), Random Forest (RF), and XGBoost models. Each model is assessed using two distinct feature sets: Base and RMT, as elaborated in the Methods section. The facet plots represent the binary classification between the different disease stages.

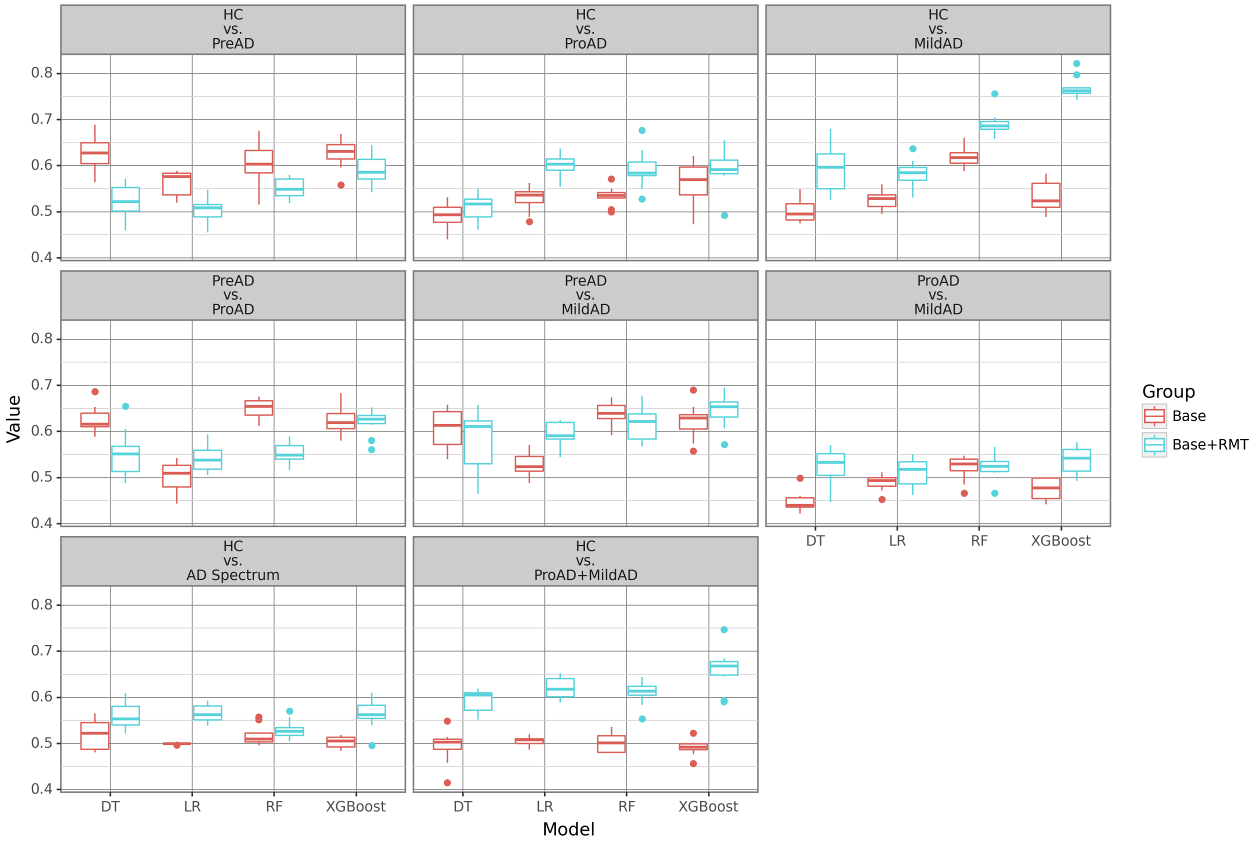

Supplementary Figure C.15: **Comparative Analysis of AUROCs for the Mezurio Data.** This figure displays boxplots of the Area Under the Receiver Operating Characteristic (AUROC) for Logistic Regression (LR), Decision Tree (DT), Random Forest (RF), and XGBoost models. Each model is assessed using two distinct feature sets: Base and RMT, as elaborated in the Methods section. The facet plots represent the binary classification between the different disease stages.

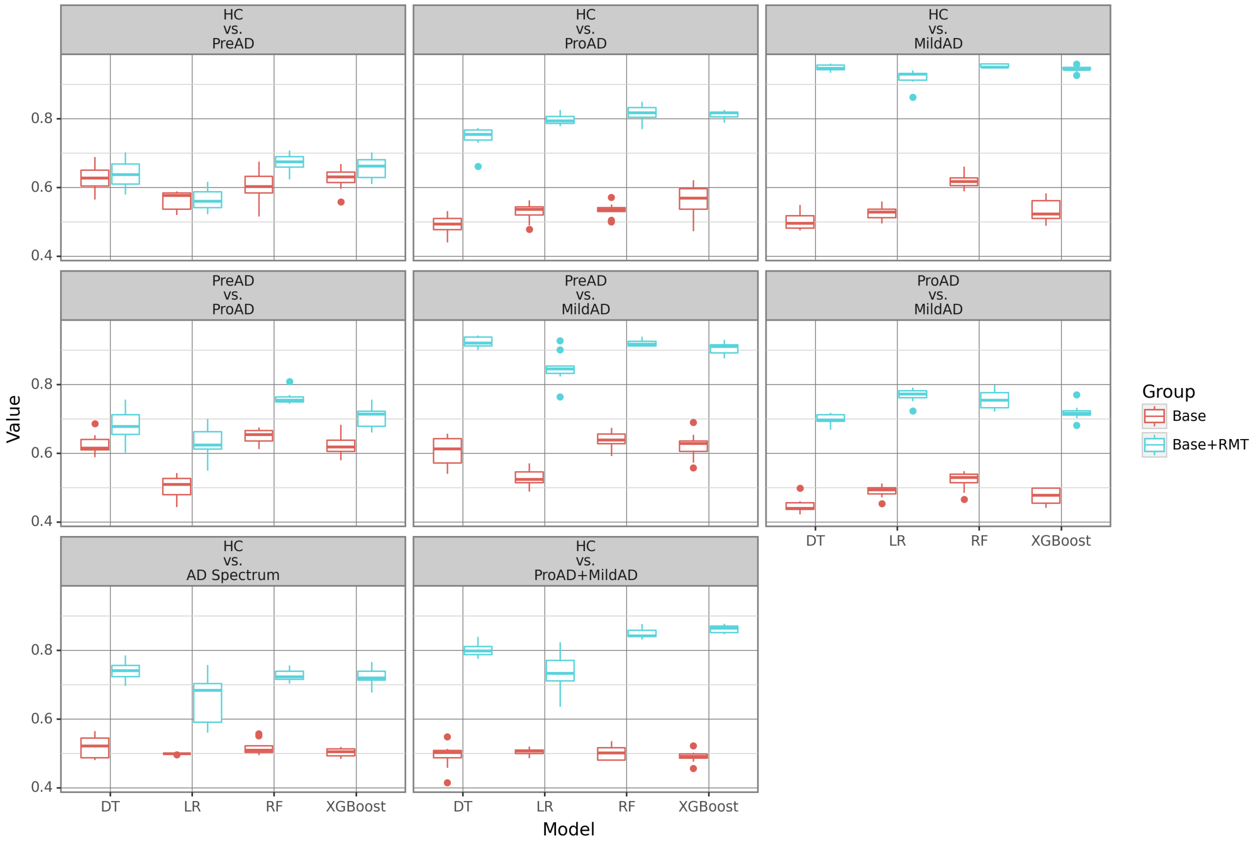

Supplementary Figure C.16: **Comparative Analysis of AUROCs for the A-iADL Data.** This figure displays boxplots of the Area Under the Receiver Operating Characteristic (AUROC) for Logistic Regression (LR), Decision Tree (DT), Random Forest (RF), and XGBoost models. Each model is assessed using two distinct feature sets: Base and RMT, as elaborated in the Methods section. The facet plots represent the binary classification between the different disease stages.

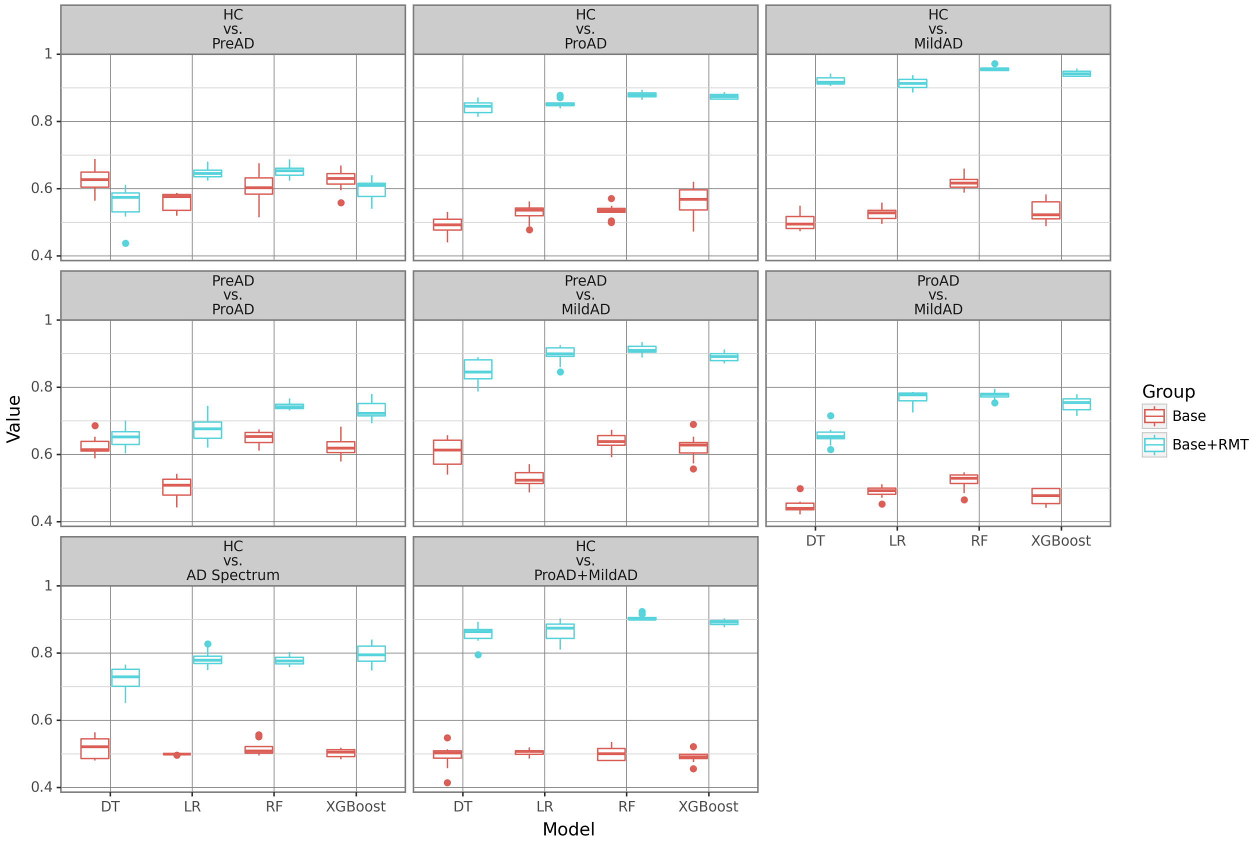

Supplementary Figure C.17: **Comparative Analysis of AUROCs for the Functional Domain Scores.** This figure displays boxplots of the Area Under the Receiver Operating Characteristic (AUROC) for Logistic Regression (LR), Decision Tree (DT), Random Forest (RF), and XGBoost models. Each model is assessed using two distinct feature sets: Base and RMT, as elaborated in the Methods section. The facet plots represent the binary classification between the different disease stages.

Appendix D Implementation details

In this work, we used several implementations provided by published libraries. Supplementary Table D.1 shows the respective libraries, their versions, and the context in which they have been used. Additionally, we make our code publicly available on https://github.com/SCAI-BIO/radar-ad-rmt-analysis.

Supplementary Table D.1: Implementation details

Study context	Library name	version
LR & RF model	scikit-learnPedregosa et al. (2011)	1.12
XGBoost model	xgboostChen and Guestrin (2016)	1.6.2
Hyperparameter optimization	Optuna	3.0.2
Metrics (AUC/AUPR)	scikit-learnPedregosa et al. (2011)	1.12
Speech processing	openSMILE Eyben et al. (2010)	2.4.1

References for Appendix

Pedregosa F, Varoquaux G, Gramfort A, Michel V, Thirion B, Grisel O, et al. Scikit-Learn: Machine Learning in Python. Journal of Machine Learning Research. 2011;12:2825–2830.

Galasko D, Bennett D, Sano M, Ernesto C, Thomas R, Grundman M, et al. An Inventory to Assess Activities of Daily Living for Clinical Trials in Alzheimer's Disease. The Alzheimer's Disease Cooperative Study. Alzheimer Disease and Associated Disorders. 1997;11 Suppl 2:S33–39.

Sikkes SAM, de Lange-de Klerk ESM, Pijnenburg YAL, Gillissen F, Romkes R, Knol DL, et al. A new informant-based questionnaire for instrumental activities of daily living in dementia. Alzheimer's & Dementia. 2012 Nov;8(6):536–543. https://doi.org/10.1016/j.jalz.2011.08.006.

Sikkes SAM, Knol DL, Pijnenburg YAL, de Lange-de Klerk ESM, Uitdehaag BMJ, Scheltens P. Validation of the Amsterdam IADL Questionnaire©, a new tool to measure instrumental activities of daily living in dementia. Neuroepidemiology. 2013;41(1):35–41. https://doi.org/10.1159/000346277.

Mack WJ, Freed DM, Williams BW, Henderson VW. Boston Naming Test: shortened versions for use in Alzheimer's disease. Journal of Gerontology. 1992 May;47(3):P154–158. https://doi.org/10.1093/geronj/47.3.p154.

Johns MW. A new method for measuring daytime sleepiness: the Epworth sleepiness scale. Sleep. 1991 Dec;14(6):540–545. https://doi.org/10.1093/sleep/14.6.540.

Hurst NP, Kind P, Ruta D, Hunter M, Stubbings A. Measuring health-related quality of life in rheumatoid arthritis: validity, responsiveness and reliability of EuroQol (EQ-5D). British Journal of Rheumatology. 1997 May;36(5):551–559. https://doi.org/10.1093/rheumatology/36.5.551.

Farias ST, Mungas D, Reed BR, Cahn-Weiner D, Jagust W, Baynes K, et al. The measurement of everyday cognition (ECog): scale development and psychometric properties. Neuropsychology. 2008 Jul;22(4):531–544. https://doi.org/10.1037/0894-4105.22.4.531.

Sheikh JI, Yesavage JA. Geriatric Depression Scale (GDS): Recent evidence and development of a shorter version. Clinical Gerontologist: The Journal of Aging and Mental Health. 1986;5(1-2):165–173. Place: US Publisher: Haworth Press. https://doi.org/10.1300/J018v05n01_09.

Kaufer DI, Cummings JL, Ketchel P, Smith V, MacMillan A, Shelley T, et al. Validation of the NPI-Q, a brief clinical form of the Neuropsychiatric Inventory. The Journal of Neuropsychiatry and Clinical Neurosciences. 2000;12(2):233–239. https://doi.org/10.1176/jnp.12.2.233.

Buysse DJ, Reynolds CF, Monk TH, Berman SR, Kupfer DJ. The Pittsburgh Sleep Quality Index: a new instrument for psychiatric practice and research. Psychiatry Research. 1989 May;28(2):193–213. https://doi.org/10.1016/0165-1781(89)90047-4.

Cherrier MM, Mendez MF, Dave M, Perryman KM. Performance on the Rey-Osterrieth Complex Figure Test in Alzheimer disease and vascular dementia. Neuropsychiatry, Neuropsychology, and Behavioral Neurology. 1999 Apr;12(2):95–101.

Birchwood M, Smith J, Cochrane R, Wetton S, Copestake S. The Social Functioning Scale. The development and validation of a new scale of social adjustment for use in family intervention programmes with schizophrenic patients. The British Journal of Psychiatry: The Journal of Mental Science. 1990 Dec;157:853–859. https://doi.org/10.1192/bjp.157.6.853.

Henry JD, Crawford JR, Phillips LH. Verbal fluency performance in dementia of the Alzheimer's type: a meta-analysis. Neuropsychologia. 2004;42(9):1212–1222. https://doi.org/10.1016/j.neuropsychologia.2004.02.001.

Heun R, Burkart M, Wolf C, Benkert O. Effect of presentation rate on word list learning in patients with dementia of the Alzheimer type. Dementia and Geriatric Cognitive Disorders. 1998;9(4):214–218. https://doi.org/10.1159/000017049.
